# LC3 forms functional nanoclusters on autophagosomes

**DOI:** 10.1038/s44319-026-00931-0

**Published:** 2026-09-25

**Authors:** Saman Fatihi, Deepak M Khushalani, Joydipta Kar, Sourav Ghosh, Aayushi Singh, Suvapriya Roy, Anirban Banerjee, Nitin Mohan, Lipi Thukral

**Affiliations:** 1 CSIR-Institute of Genomics and Integrative Biology, Mathura Road, New Delhi, 110025 India; 2 Academy of Scientific and Innovative Research (AcSIR), Ghaziabad, 201002 India; 3 Department of Biological Sciences and Bioengineering, Indian Institute of Technology Kanpur, Kalyanpur, Uttar Pradesh 208016 India; 4 Department of Biosciences and Bioengineering, Indian Institute of Technology Bombay, Mumbai, Maharashtra India

**Keywords:** Autophagy & Cell Death, Membranes & Trafficking, Microbiology, Virology & Host Pathogen Interaction

## Abstract

Autophagosome biogenesis depends on the coordinated action of proteins and lipids. However, how LC3B organizes at high density and contributes to autophagy-associated functions remains unclear. Using molecular dynamics simulations and super-resolution microscopy, we show that LC3B self-assembles into higher-order nanoclusters approximately 150 nm in size. These clusters form spatially distinct LC3B “islands” on the autophagosome membrane and show a preferential association with phosphatidylinositol-3-phosphate (PI3P) lipids. Molecular analysis of 296 structural clusters reveals a putative LC3B homo-clustering interface characterized by a distinct alternating hydrophobic–polar motif. To experimentally validate this interface, we generated four LC3B mutants targeting the clustering motif. STORM imaging of the LC3 mutants demonstrates a complete alteration in clustering dynamics, with diffuse, smaller LC3 clusters on the autophagosome. Further, motility analysis and interaction with the autophagic adaptor protein FYCO1 show abrogated autophagosome motility. Finally, loss of LC3B nanoclustering also significantly compromises autophagic entrapment of *Streptococcus pneumoniae* and results in elevated intracellular bacterial survival. Taken together, our results reveal LC3 clustering as a potential organizing principle that coordinates the spatial and temporal dynamics of autophagy.

## Synopsis

Super-resolution microscopy combined with molecular dynamics simulations reveals that LC3B self-assembles through a defined clustering interface. This organization is essential for autophagosome motility and the clearance of bacterial cargo.

- LC3 self-assembles into ~150 nm nanoclusters that preferentially localize to PI(3)P-enriched regions of the autophagosome membrane.
- Clustering is mediated by a specific interface involving loop 6 and helix α4, defined by an alternating hydrophobic–polar motif.
- Disrupting LC3 clustering impairs FYCO1-dependent autophagosome motility and bacterial cargo clearance, increasing intracellular bacterial survival.

## Abstract (web summary)

Super-resolution microscopy combined with molecular dynamics simulations reveals that LC3B self-assembles through a defined clustering interface. This organization is essential for autophagosome motility and the clearance of bacterial cargo.

## Graphical abstract

**Figure:**
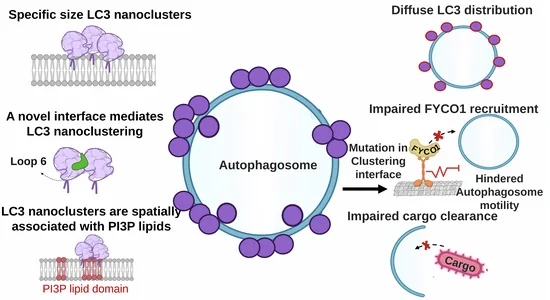

## Introduction

The autophagosome is a unique double-membrane vesicle formed entirely de novo during the process of autophagy (Suzuki et al, 2001; Nakatogawa et al, 2009). The biogenesis of autophagosomes is tightly regulated by a complex interplay of autophagy-related proteins, predominantly driven by their interactions with membrane lipids (Yang and Klionsky, 2010; Mizushima et al, 2011). Central to this process is the LC3B protein, which plays a crucial role at multiple stages of autophagy (Kabeya et al, 2000). Upon autophagic induction, LC3B translocates to the growing phagophore, where it facilitates phagophore elongation, autophagosome maturation, and ultimately facilitates fusion with lysosomes for cargo degradation (Nakatogawa et al, 2007; Xie et al, 2008). A key step in this process is the conjugation of cytoplasmic LC3B to phosphatidylethanolamine (PE), producing the membrane-bound form LC3B-II (Ichimura et al, 2004; Tanida et al, 2004; Kirisako et al, 2000). The lipidated form of LC3B specifically associates with autophagosomal membranes, serving as a key marker of autophagic activity. Its presence in a punctate, spot-like appearance reflects the abundance and spatial localization of autophagosomes within the cell (Tanida and Waguri, 2010). Fluorescent labeling distinguishes between the cytosolic and membrane-bound forms of LC3B, where a diffuse cytosolic pattern indicates low autophagic activity, while increased puncta number and size signify elevated autophagic flux (Kaizuka et al, 2016). While the interaction of LC3B with phosphatidylethanolamine (PE) and phosphatidylserine (PS) through lipid anchoring is well-established, the dynamics of LC3B and its interactions with other membrane lipids remain poorly understood (Sou et al, 2006). Thus, there exists a significant gap in our understanding of how LC3B proteins are organized on a diverse array of lipids on the autophagosome membrane. This gap arises partly from the challenges inherent in studying small membrane-associated proteins using structural biology techniques, as well as the lack of a molecular model that elucidates the organization of concentrated lipidated LC3B proteins on membranes. To bridge this gap, we employed a combination of molecular simulations and super-resolution imaging to explore the dynamic organization of LC3B tethered to membranes via a lipid anchor under crowded conditions. Despite established LC3B functions across these steps, it remains unknown how LC3B is spatially organized on the autophagosome membrane during different stages of autophagosome biogenesis and whether this organization influences autophagosome maturation and function.

The structural understanding of LC3B has significantly advanced in recent years, shedding light on its multifaceted roles in autophagy (Wirth et al, 2019; Ye et al, 2023; Rogov et al, 2023). Its structure consists of an N-terminal helical region and a ubiquitin-like domain that provides sites for interaction with autophagy adapters and membrane components. LC3 features two canonical hydrophobic binding pockets that enable LC3 Interacting Region (LIR)-mediated interactions with autophagy receptors, such as p62, NBR1, and optineurin, bridging LC3 with cargo destined for degradation. Human LC3 proteins consist of six isoforms: LC3A, LC3B, LC3C, GABARAP, GABARAPL1, and GATE16. They are closely related yet perform distinct functions (Weidberg et al, 2010). Our previous work on LC3B (hereafter referred to as LC3) has elaborated on its membrane-targeting motif (Zhang et al, 2023), its spontaneous binding process during lipidation (Thukral et al, 2015), and the role of LC3 human homologs in shaping diverse functional outcomes (Jatana et al, 2019).

Recent advances in super-resolution microscopy have surpassed the diffraction limit of conventional light microscopy, enabling visualization of cellular structures and molecular interactions at the nanometer scale (Huang et al, 2010). Single-molecule localization techniques such as stochastic optical reconstruction microscopy (STORM) and photo-activated localization microscopy (PALM) provide lateral resolution of ~20 nm, making them ideal for studying protein clusters (Rust et al, 2006), large molecular complexes (Wurm et al, 2011; Guizetti et al, 2011), and detailed visualization of cellular ultrastructure (Mennella et al, 2012; Lillemeier et al, 2010). Further, the nanometer resolution also allows precise quantification of stoichiometry, as well as relative positioning of membrane-bound proteins on cellular organelles (Owen et al, 2010). Recently, with structured illumination microscopy (SIM), with ~100 nm resolution, it was shown that autophagosome precursors emerge from recycling endosomes with complex morphologies, revealing finger-like structures that mature into spherical forms (Puri et al, 2023). However, studying the dynamic organization of proteins at the atomistic level on membranes remains a challenge. Molecular dynamics (MD) simulations have emerged as a powerful tool to complement these limitations, providing rich insights into biological interactions, particularly protein–lipid dynamics. Small peripheral membrane proteins, like LC3B, provide an excellent opportunity for investigation through these complementary approaches, offering insights into their dynamics, interactions, and functional roles with high precision.

In this study, we combined coarse-grained simulations with super-resolution imaging and functional analysis to explore how LC3 organizes on autophagosomal membranes under crowded conditions. Although LC3 is generally viewed as a diffusely distributed membrane marker, both approaches unexpectedly reveal unique patterns of organization that persisted across different stages of autophagy. To probe what might underlie this behavior, we examined a small set of LC3 mutants designed to perturb potential self-association features. This analysis pointed to a clustering interface on LC3 that may contribute to its assembly behavior. Complementary assays further showed that LC3 clustering modulates engagement of the motor protein and influences autophagosome motility. Notably, disrupting LC3B nanoclusters had a significant impact on impairment of bacterial cargo capture, leading to reduced autophagosomal engulfment and increased intracellular bacterial burden.

## Results

### Coarse-grain molecular simulations show clusters of lipidated LC3 on model membranes

**Figure 1:**
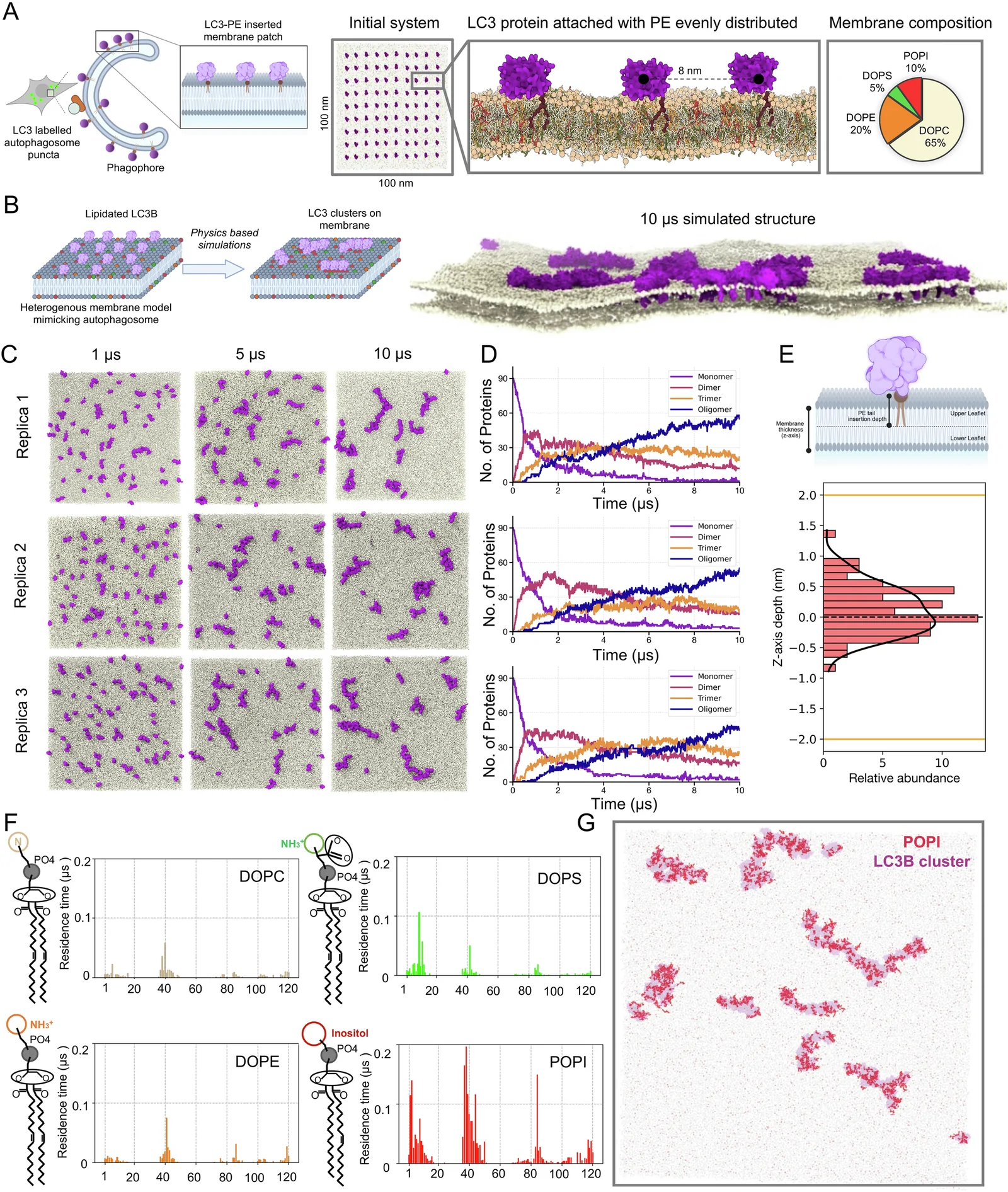
Lipidated LC3 self-assembles to form clusters on autophagosomes. (**A**) Snapshot of the initial simulation setup with 90 LC3B molecules covalently conjugated to PE and uniformly placed on the outer leaflet of the membrane at 8 nm spacing. The membrane is composed of heterogeneous ER lipids, including DOPC, DOPE, DOPS, and POPI. (**B**) Schematic of the simulation model and a representative snapshot illustrating the emergence of non-uniform LC3B nanoclusters (front view). (**C**) Representative snapshots from three coarse-grained simulations showing a consistent tendency of LC3B to self-assemble across all replicas, shown at different time points. The 1 μs snapshot of Replica 1 is reproduced in Fig. EV2A for comparison with the other LC3 homologs. (**D**) Over the course of the simulations, dimers and trimers initially formed, followed by the emergence of higher-order LC3 oligomers within 3 μs. (**E**) Density plot showing the distribution of PE-anchor insertion depths for individual LC3B molecules within the outer leaflet headgroup region. (**F**) The plot shows the residue-wise residence time for different lipid types. Residence times for each lipid species were calculated based on their proximity to LC3 residues. (**G**) Top view highlighting the accumulation of PI lipids in the vicinity of LC3 clusters. Source data are available online for this figure.

To understand LC3 organization on membrane in a crowded environment, we generated a physiologically relevant model with multiple lipidated LC3B proteins on heterogeneous membrane mimicking autophagosomal lipids (Fig. 1A). The LC3 proteins were uniformly distributed, with each protein spaced roughly 8 nm apart, covering 26% of the membrane surface. As shown in Appendix Fig. S1, the membrane orientation was consistent with our previously identified membrane interaction motif (Zhang et al, 2023). We observed that LC3 proteins formed higher-order clusters by the end of the initial simulation (Fig. 1B; Movie EV1). To validate this observation, two additional simulations, each 10 μs long, were performed. Across all replicas, a consistent tendency for all proteins to cluster was observed (Fig. 1C). Dimers initially formed within 500 ns, and by the end of the simulation, nearly all LC3 molecules were clustered (Appendix Fig. S2). The interactions were predominantly limited to two neighboring LC3 proteins, although depending on topology, interfaces involving three or more proteins were also observed (Fig. 1D; Appendix Fig. S2). All LC3 clusters were properly conjugated to the membrane, with the PE anchor consistently embedded within the headgroups of the upper leaflet for each LC3 protein throughout the simulation (Fig. 1E). Although the membrane exhibited pronounced global undulations, LC3 clusters did not preferentially localize to regions of high local curvature (Appendix Fig. S3). We found that local curvature in the vicinity of clusters remained comparable to that of distal membrane regions, suggesting that LC3 clustering is largely independent of these membrane fluctuations.

To identify whether LC3 is enriched on any particular lipid moieties, we performed protein–lipid interaction analysis. We calculated residue-wise lipid contacts for each protein and normalized these values by lipid concentration to enable direct comparisons across lipid types (Fig. 1F). Among the positively charged residues within the membrane-targeting module, the inositol groups displayed specific interactions with three key residues Arg37, Lys39, and Lys42 located at the start of loop 3. Together, these residues form a cleft that accommodates the PI headgroup. PI lipids form enriched lipid platforms, and probability density plots also revealed that these PI-rich regions colocalize with LC3 clusters (Appendix Fig. S4). Closer examination of these clusters showed that PI lipids are concentrated at the center and in the vicinity of the LC3 assemblies, far exceeding the levels of other phospholipid species (Fig. 1G). Our findings indicate that PE-conjugated LC3 has a strong tendency to self-assemble on the membrane, forming higher-order oligomers that are highly enriched in PI lipids.

### Super-resolution microscopy confirms formation of LC3 nanoclusters on autophagosomes

**Figure 2:**
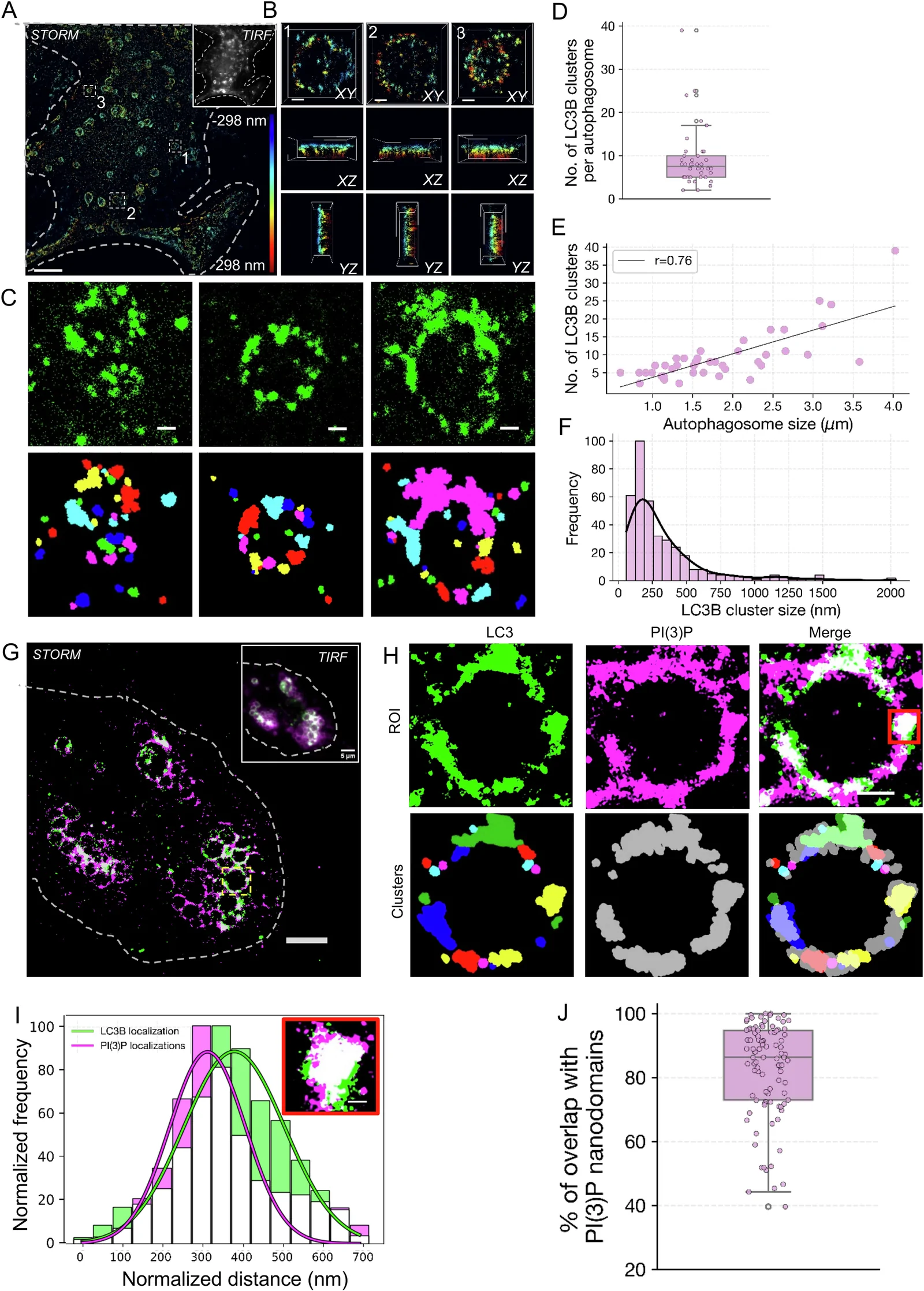
TIRF and STORM imaging of autophagosomes showing LC3 nanoclusters. (**A**) Representative super-resolution 3D-STORM imaging of a starved BS-C-1 cell immunostained for endogenous LC3B. The inset shows the diffraction-limited TIRF image of the same cell, highlighting LC3B-positive autophagosomes. (**B**) Representative regions of interest (ROIs) from (A), showing the spatial organization of LC3B on mature autophagosomes in XY, XZ, and YZ planes. (**C**) Representative 2D-STORM images of LC3B decorated on autophagosomes of different sizes, along with corresponding DBSCAN-segmented clusters. In the segmented image, different colors represent individual LC3B nanoclusters. (**D**–**F**) Quantification of endogenous LC3B nanoclusters on autophagosomes. (**D**) Box plot showing the number of LC3B nanoclusters per autophagosome. (**E**) Scatter plot showing correlation between the number of LC3B clusters and autophagosome size. (**F**) Histogram and Gaussian fitting analysis of STORM-resolved LC3 assemblies showing that the predominant LC3-positive membrane-associated assemblies are centered around ~150–300 nm, representing the dominant organizational state observed across autophagosomes. Only a small fraction of assemblies (~5.5%) correspond to larger supramolecular LC3 structures. Data were obtained from 8 cells across two independent experiments. (**G**) Sequential two-color 2D-STORM imaging of starved BS-C-1 cells expressing mCherry-2xFYVE, immunostained for endogenous LC3B and mCherry. The inset shows a diffraction-limited TIRF image of the LC3 (green) and mCherry-2xFYVE (magenta). (**H**) Yellow ROI from (**G**) with corresponding DBSCAN segmentation analysis, demonstrating the spatial organization of LC3B nanoclusters on PI(3) P-enriched autophagosomes. (**I**) Corresponding localization histogram of ROI (red) from (**H**). (**J**) Quantification of the % overlap between LC3 clusters and PI(3)P nanodomains. Data were obtained from four cells across two independent experiments. For all box plots, the center line denotes the median, box bounds denote the 25th and 75th percentiles (interquartile range, IQR), and whiskers extend to the most extreme data points within 1.5 × IQR of the box bounds; all individual data points are overlaid. Minima and maxima are represented by overlaid data points. Scale bars: 5 µm for (**A**, **G**); 1 µm for (**H**); 200 nm for (**C**, **I**). Source data are available online for this figure.

Observations from the simulations prompted us to experimentally resolve and validate the self-organization of LC3 on autophagosomes. Towards this, we performed super-resolution STORM imaging of LC3 in starved BS-C-1 cells (Fig. 2A,B). While conventional TIRF imaging of LC3 depicts diffraction-limited autophagosome puncta within the cell (Fig. 2A inset; Appendix Fig. S5), 3D-STORM imaging allows us to resolve the spatial distribution of LC3 on each autophagosome. At 20 nm spatial resolution, we visualize that membrane-bound LC3 forms spatially distinct clusters, spanning the autophagosomal membrane (Fig. 2C; Movies EV2–EV4). Quantitative analysis of LC3 clusters using DBSCAN, enumerates significant heterogeneity in the number of clusters, with autophagosomes containing 5–25 LC3 clusters, and an average of 9 LC3 clusters per autophagosome (Fig. 2D; Appendix Fig. S6). We hypothesized that the heterogeneity in LC3 cluster numbers is driven by the variability in autophagosome size, which ranges from a few nm to 3 μm in diameter within cells. To test this, we analyzed the relationship between LC3 cluster numbers and autophagosome size, observing a clear linear correlation (Fig. 2E). This confirms that larger autophagosomes can accommodate a greater number of LC3 clusters.

Next, when we analyse the size of the LC3 clusters, we observed a peak size distribution of LC3 clusters at 150 nm. Notably, the very large supramolecular cluster assemblies (~1000 nm) represented only a small fraction (~5%) of the total assemblies measured (Fig. 2F; Appendix Fig. S6). Interestingly, unlike cluster numbers, LC3 cluster size does not scale linearly with autophagosome size (Appendix Fig. S7). Instead, LC3 clusters maintain a consistent size range of 100–300 nm, regardless of autophagosome dimensions. To distinguish whether LC3 clusters emerge from progressive LC3 recruitment or alternatively due to larger LC3 assemblies dissipating into smaller, lower-density structures during autophagosome maturation, we performed additional quantitative analysis of LC3 nanocluster organization. We first quantified LC3 localization density by dividing the number of localizations within each cluster by its area and examined its relationship with cluster size. We found that LC3 localization density was largely independent of cluster size (r = −0.15), suggesting it is unlikely that initial high-density clusters dissipate over time into smaller, sparser ones. Instead, we found a positive linear correlation (r = 0.62) between the total number of LC3 localizations on each autophagosome and the autophagosome size, confirming that more LC3 accumulates as the autophagosome grows (Appendix Fig. S8). However, the oligomeric clusters observed in the molecular dynamics simulations are constrained by the finite system sizes and timescales accessible to simulation. In contrast, the larger membrane-associated structures detected by STORM likely represent higher-order assemblies composed of multiple LC3 oligomeric clusters. To distinguish these larger structures from the smaller oligomers observed in simulations, we hereafter refer to the STORM-resolved structures as LC3 nanocluster assemblies.

**Figure EV1:**
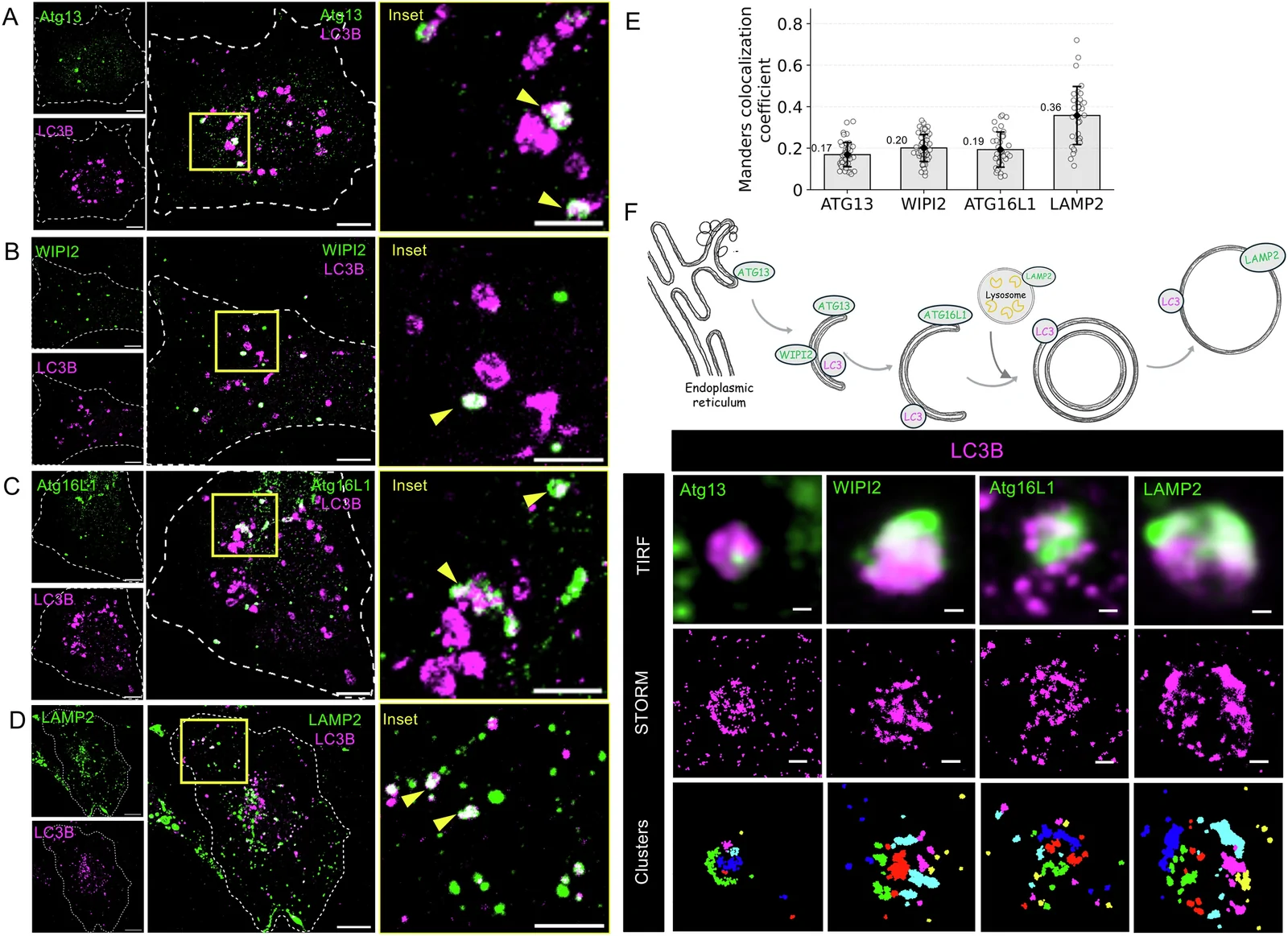
LC3 forms nanoclusters on Atg13-, WIPI2-, ATG16L1-, and LAMP2-positive vesicles. (**A**–**D**) Representative dual-color confocal images of LC3B (magenta) with ATG13, WIPI2, ATG16L1, or LAMP2 (green). Yellow boxes indicate enlarged regions highlighting double-positive vesicles. (**E**) Manders’ colocalization coefficient quantifying colocalization of LC3 with ATG13, WIPI2, ATG16L1, and LAMP2. Data are shown as mean ± SD from 50 cells (ATG13), 54 cells (WIPI2), 34 cells (ATG16L1), and 36 cells (LAMP2), pooled from two independent experiments. (**F**) Schematic representation of autophagy pathway along with dual-color TIRF images of LC3 (magenta) with ATG13, WIPI2, ATG16L1, or LAMP2 (green), with corresponding STORM images showing the spatial organization of LC3 nanoclusters on marker-positive vesicles. Scale bars: 10 µm (full images), 5 µm (zoomed insets, **A**–**D**), and 500 nm (**F**).

In the autophagic pathway, initiation, maturation, closure, and fusion with lysosomes represent sequential phases through which the phagophore evolves into a mature autophagosome. Dual-color TIRF and STORM imaging revealed that LC3 forms nanoscale membrane-associated assemblies on vesicles positive for ATG13, WIPI2, ATG16L1, and LAMP2, indicating that LC3 nanoclustering is maintained throughout the stages of autophagosome biogenesis, maturation and fusion with lysosomes. Notably, LC3 nanoclusters were detected on autophagic structures marked by ATG13, WIPI2, ATG16L1 and on LAMP2-positive autolysosomes, suggesting that higher-order LC3 organization is preserved throughout the autophagic pathway (Fig. EV1A–F).

**Figure EV2:**
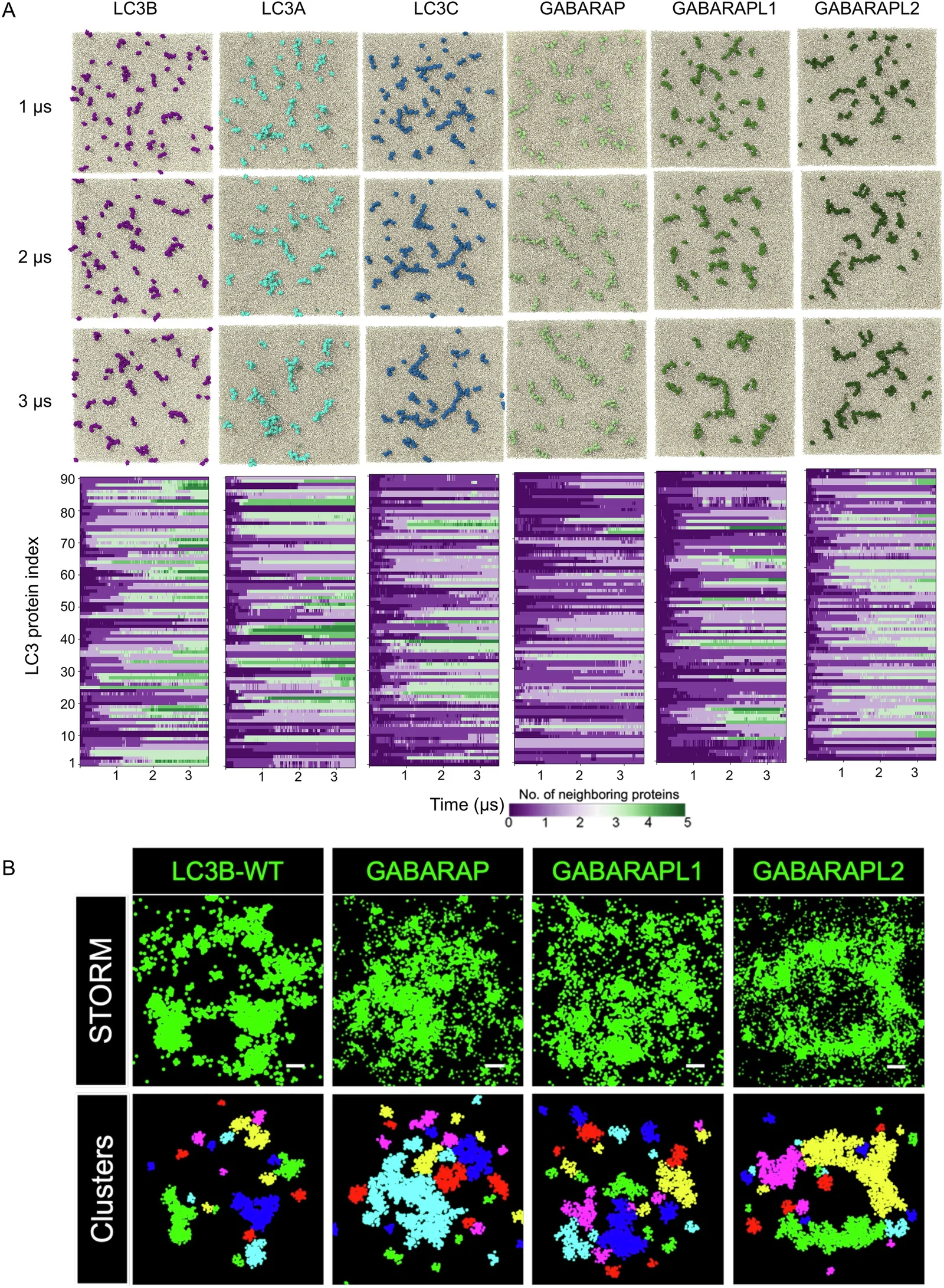
Simulations and STORM imaging reveal cluster formation of Atg8-family homologs. (**A**) Representative snapshots from coarse-grained molecular dynamics simulations of the human Atg8-family homologs LC3A, LC3B, LC3C, GABARAP, GABARAPL1, and GABARAPL2 (GATE16) at 1, 2, and 3 µs. The LC3B 1 μs snapshot is reproduced from Fig. 1C for comparison with the other LC3 homologs. All homologs exhibit spontaneous membrane-associated clustering qualitatively similar to LC3B, indicating that clustering is a conserved feature of the Atg8 family rather than a property unique to LC3B. The lower panel shows a comparative analysis of clustering organization across the six homologs. Heatmaps show the number of protein–protein interfaces formed by each monomer over time, revealing distinct clustering topologies despite the shared propensity to self-associate. GABARAP predominantly forms linear assemblies characterized by two interfaces per molecule, whereas LC3B displays more heterogeneous and interconnected interaction networks. (**B**) Representative STORM images of individual autophagosomes from starved BS-C-1 cells transiently expressing LC3B, GABARAP, GABARAPL1, and GABARAPL2, demonstrating that all homologs form discrete nanoscale clusters on autophagosomes in cells. Scale bar:200 nm.

Additionally, we asked if the membrane-associated nanoscale organization is restricted to LC3B or is it a broader feature across the human ATG8 family. Comparative coarse-grained simulations across all six human ATG8 homologs (LC3A, LC3B, LC3C, GABARAP, GABARAPL1, and GABARAPL2/GATE16) revealed that membrane-associated clustering is broadly conserved across the ATG8 family (Fig. EV2A). However, distinct assembly topologies emerged between homologs, with GABARAP-family proteins forming comparatively elongated and interconnected assemblies relative to the more heterogeneous LC3-family organizations. To further validate these observations, additional STORM imaging experiments were performed for GABARAP, GABARAPL1, and GABARAPL2, which similarly exhibited prominent membrane-associated nanoscale assemblies with spatial organization distinct from LC3B (Fig. EV2B). Furthermore, mixed 50:50 LC3B–GABARAP simulations demonstrated frequent co-clustering of LC3B and GABARAP molecules within shared membrane-associated assemblies, suggesting that heterotypic ATG8 assemblies may occur on membranes (Appendix Fig. S9). Given the extensive mechanistic complexity underlying homolog-specific clustering, atomistic interaction networks, and lipid-mediated organization, in the present study, we focused our detailed molecular and experimental analyses primarily on LC3B to dissect the fundamental interaction principles governing membrane-associated nanoscale assembly formation.

#### LC3B clusters preferentially associate with PI(3)P lipids

**Figure EV3:**
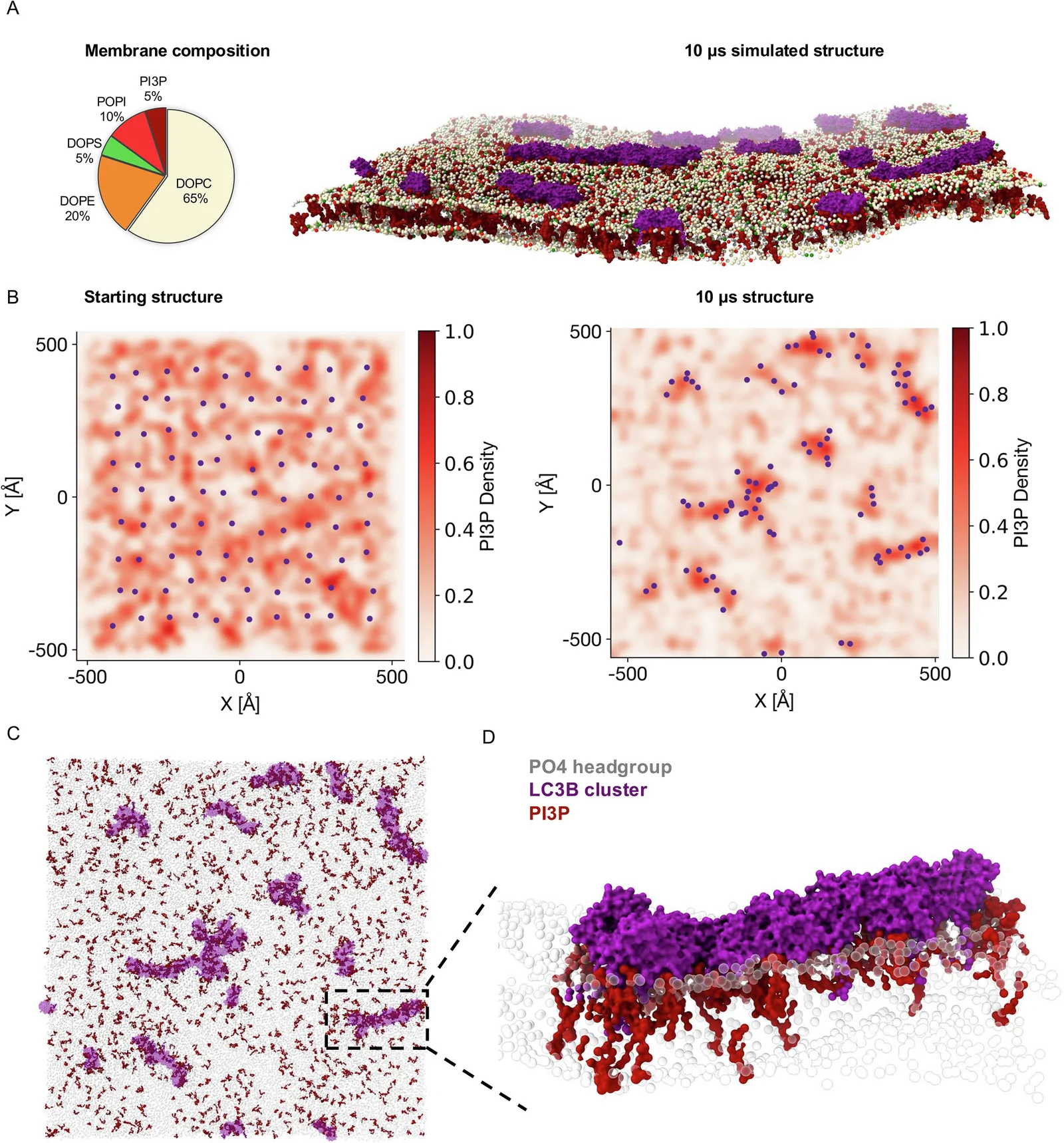
PI3P enrichment around LC3 clusters in coarse-grained simulations. (**A**) Simulation setup and representative membrane configuration after 10 μs showing LC3 clusters (purple) on a mixed lipid bilayer. (**B**) Probability density maps of PI3P projected onto the membrane plane. Purple dots indicate LC3 positions. Regions occupied by LC3 clusters coincide with areas of elevated PI3P density. (**C**) Top view highlighting the spatial colocalization of LC3 clusters (purple) and PI3P lipids (red). (**D**) Zoomed view showing accumulation of PI3P headgroups beneath membrane-bound LC3 assemblies.

Among all phosphoinositides, PI(3)P is well-established as a key player in autophagy initiation (Yu et al, 2017; Yim and Mizushima, 2020; Nascimbeni et al, 2017). Therefore, we transiently overexpressed a PI(3)P lipid sensor, mCherry-tagged tandem-FYVE domain of the HRS protein, which specifically binds to membranes decorated with PI(3)P lipids. Cells were starved for 2.5 h, followed by a sequential immunostaining and 2D-STORM imaging, as detailed in the methods. Strikingly, we observed that PI(3)P lipids also form spatially distinct nanodomains on the autophagosome surface. Furthermore, LC3 clusters were found to colocalize with PI(3)P nanodomains (Fig. 2G,H). DBSCAN-based image segmentation revealed a strong similarity in the morphology and spatial distribution of LC3 clusters and PI(3)P nanodomains, suggesting a coordinated nanoscale organization (Fig. 2H lower panel). To eliminate probe-dependent artifacts, we repeated the experiment with the p40phox-PX PI(3)P sensor and observed similar colocalization with LC3 (Appendix Fig. S10). We quantified the spatial overlap between LC3 and PI(3)P and found that, on average, ~65% of each LC3 cluster colocalized with PI(3)P (Fig. 2I–J). Consistent with the imaging data, we also performed additional simulations on PI(3)P-containing membranes as opposed to simpler phosphoinositide lipids. Trajectories revealed the emergence of similar PI(3)P-enriched membrane nanodomains in the vicinity of LC3 clusters (Fig. EV3). Together, the experimental and computational results support a close coupling between LC3 mesoscale assembly and local lipid organization, suggesting that protein self-association and membrane nanodomain formation are interconnected processes during autophagosome organization.

### LC3 self-assembly is driven by a unique clustering interface

**Figure 3:**
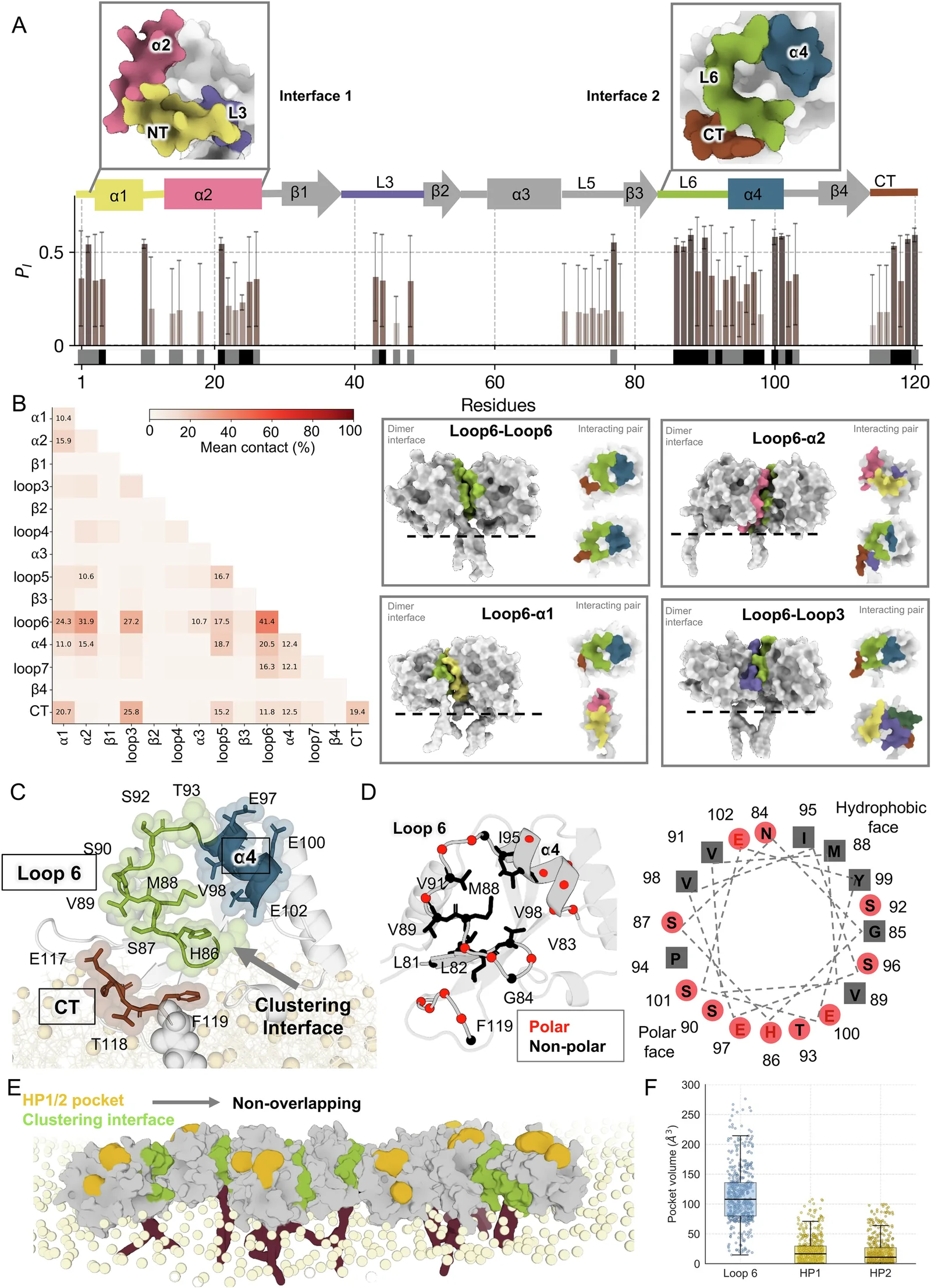
Identification of the membrane-bound LC3–PE clustering interface. (**A**) Residue-wise interaction probability plot showing the likelihood of each residue to participate in the clustering interface across all three simulations. Contacts were defined using a 10 Å distance cutoff and a persistence of >30% over the trajectory. The accompanying heatmap highlights residues in direct contact (black) and peripheral residues (gray). Snapshots illustrate the two most probable contiguous interfaces, with Interface 1 positioned on the front of the protein and Interface 2 on the rear. (**B**) Heatmap depicting domain-wise contributions to the LC3–LC3 interface, highlighting the most dominant binding mode. The color gradient corresponds to the average percentage of contacts aggregated from 296 dimer interfaces. From the dimer–dimer interface analysis, the four most prominent interface combinations are highlighted in boxes. For each, the complementary interface is shown alongside the corresponding monomer interfaces for clarity. (**C**) The rear side of the protein constitutes the most probable clustering interface, with the interacting residues shown in stick representation. (**D**) The novel clustering interface had a surface fingerprint that showed alternating polar and hydrophobic residues, a pattern found in many self-assembly proteins. Alongside, individual residues are shown to illustrate the polar/non-polar arrangement. (**E**) One of the higher-order clusters with concomitant marking of canonical hydrophobic receptor binding pockets and clustering interface at rear side that are non-overlapping. (**F**) The box plot shows the pocket volume of three pockets. Pocket volumes were calculated for all dimer interfaces (*n* = 262 dimer pairs), with measurements performed for both protomers in each dimer (*n* = 524 pocket volume measurements). Each dot represents one measurement. In each box plot, the center line indicates the median, the box bounds indicate the 25th and 75th percentiles (interquartile range, IQR), and the whiskers extend to the most extreme data points lying within 1.5 × IQR of the lower and upper box bounds. Loop 6 pockets are markedly larger (median, 107.9 Å³; IQR, 80.0–135.8 Å³) than HP1 (median, 16.6 Å³; IQR, 0.9–29.4 Å³) or HP2 (median, 10.9 Å³; IQR, 1.9–26.8 Å³). Source data are available online for this figure.

The simulations provided a unique opportunity to define the LC3 clustering interface region across a diverse set of protein clusters engaged with the membrane. Across three independent 10-µs long simulation trajectories, we extracted ~28 clusters, yielding a dataset of 296 distinct interacting surfaces. Analysis of these interfaces revealed 44 residues that consistently participate in clustering originating from α1, α2, loop 6, α4, and the C-terminal regions. Although dispersed in sequence, these residues organize into two structurally continuous interface motifs (Fig. 3A; Appendix Fig. S11). Among the 44 residues, a subset forms direct contacts (<5 Å), while others are neighboring residues positioned within 5–8 Å of the center. Together, they define an interaction surface consisting of both direct-binding and interface-supporting residues. The first interface region is located in the N-terminal regions of α1–2 and a few residues of loop 3, which is partially membrane-embedded. The second interface is formed by loop 6 at the core of the interface, flanked by peripheral residues from α4 and the C-terminal region (residues 86–98, 100–103, 114–119).

To investigate interface complementarity, we considered not just individual residues but entire residue pairs, allowing us to characterize the dominant binding modes. We quantified the relative contribution of each domain from the cumulative contact map of the three simulations. This analysis revealed heterogeneity among the interface contacts, with the four most dominant binding modes reflecting the structural variability of LC3 cluster interactions arising from interface 2 region (Fig. 3B). Structural analysis of representative clusters for each mode revealed that peripheral residues in the N-terminal and loop 3 regions engage in short-lived, transient contacts, whereas loop 6 consistently formed stable interactions with other loop 6 or α2 residues within 200 ns, serving as an early interaction hub (Appendix Fig. S12).

To understand what molecular features, make these contacts persistently “sticky,” we analyzed interface 2 in detail. We defined this region as the “rear-side clustering interface” (Fig. 3C). Structural inspection revealed a distinct pocket, formed by loop 6 and α4 around this region. The defined clustering interface has three notable features: In 43 experimentally solved LC3 complexes with peptides or proteins, this region is unoccupied, suggesting a potential site for self-interaction (Appendix Fig. S13). The pocket exhibits a pattern of alternating polar (S87, S90, S91, and T93) and hydrophobic residues (M88, V89, and V91), forming a sticky module (Fig. 3D). This pattern aligns with the “binary code” concept, where alternating polar and non-polar residues facilitate both specificity and adaptability in protein–protein interfaces (Kamtekar et al, 1993; Koide et al, 2007; Xu et al, 2001). These residues are highly conserved, corresponding to the sequence “SMVSVS” (Appendix Fig. S14).

We hypothesized that residues promoting clustering must be highly accessible to allow adjacent proteins to assemble efficiently. To test this, coarse-grained simulations were backmapped to atomistic resolution and minimized for 100 ns. We computed surface accessibility for both pre-clustering and post-clustering structures (Appendix Fig. S15). Nearly 50% of residues in clustering interfaces were accessible, supporting their role in facilitating self-assembly. Importantly, this rear binding pocket is distinct from the canonical HP1 and HP2 hydrophobic binding sites (Fig. 3E). Across simulations, residues involved in receptor interactions remain surface-accessible, preserving the potential for canonical receptor engagement (Appendix Fig. S16). In contrast, the rear binding pocket, with a volume of roughly 115 Å³, enables LC3 proteins to self-assemble on the membrane (Fig. 3F).

Interestingly, analysis of crystal packing interfaces across 69 ATG8-family structures revealed recurrent intermolecular contacts involving the Loop6–α4–C-terminal surface. Strikingly, SMVSVS motif residues (M88, V89, S90, V91, and S92) appeared in crystallographic contacts in up to 43% of all structures, the highest of any region in the protein. This interaction hotspot was consistently observed across all human ATG8 homologs and yeast Atg8, suggesting an intrinsic structural propensity for weak intermolecular association (Appendix Fig. S17). Together with the simulation and imaging analyses, these observations support a model in which the Loop6-associated surface contributes to membrane-associated nanoscale LC3 organization.

### Generating LC3 mutants from simulations to alter the clustering interface

**Figure 4:**
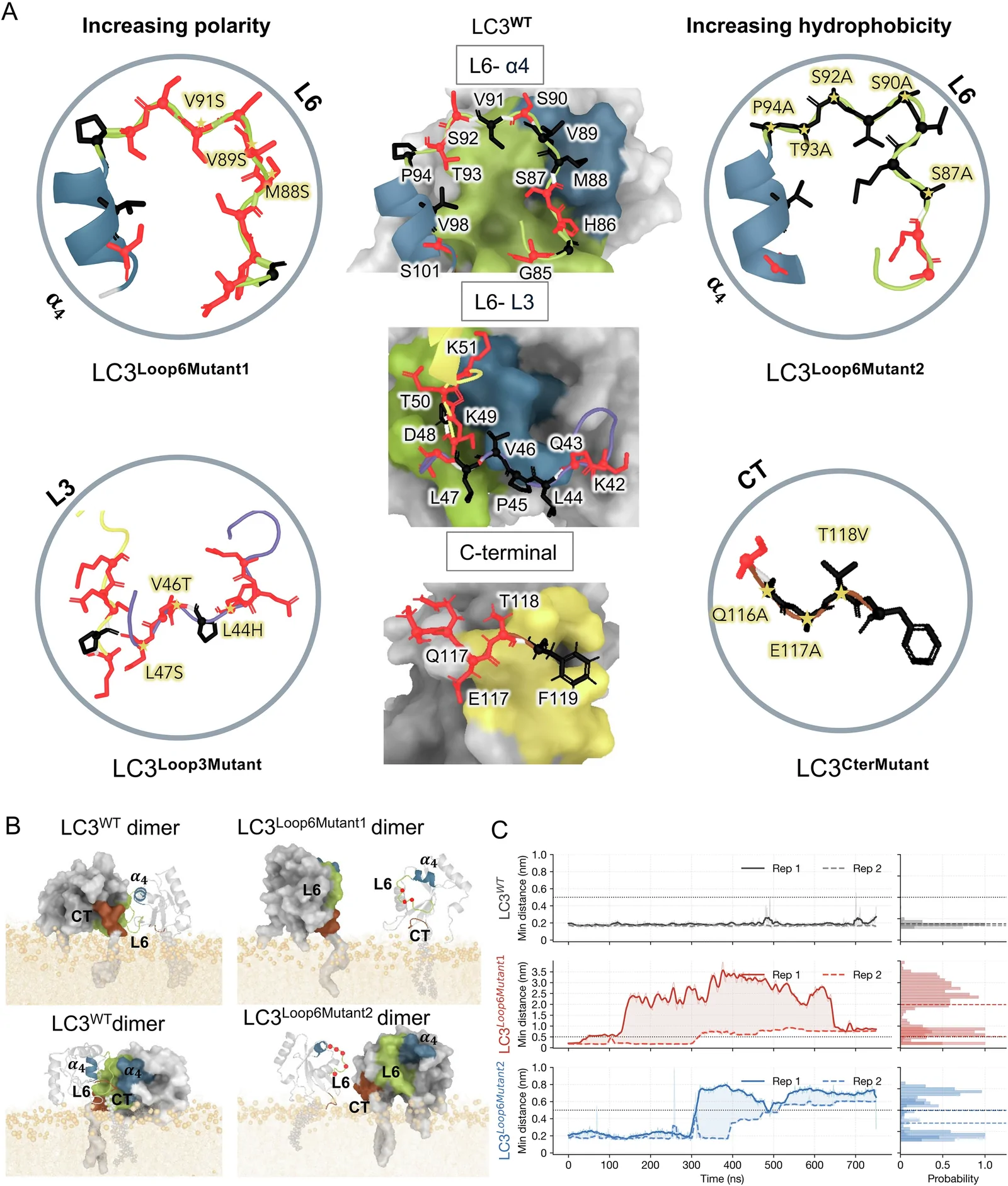
Designing mutants to abrogate LC3B clustering. (**A**) Structural representations of the wild-type LC3 interfaces are highlighted. Key residues at the interface were targeted for mutagenesis to disrupt clustering. Zoomed-in structural models display the side chains of the designed mutants, illustrating changes in properties, either increased polarity (LC3^Loop6Mutant1^: M88S, V89S, V91S; LC3^Loop3Mutant^: L44H, V46T, L47S) or increased hydrophobicity (LC3^Loop6Mutant2^: S87A, S90A, S92A, T93A, P94A; LC3^**CterMutant**^: Q116A, E117A, T118V), with the mutated residues marked by a star and labeled. (**B**) Representative membrane-bound structures from atomistic MD simulations of LC3^WT^, LC3^Loop6Mutant1^, and LC3^Loop6Mutant2^. While LC3^WT^ maintains a stable dimer interface, both Loop6 mutants sample dissociated configurations with loss of intermolecular contacts. (**C**) Time evolution of the minimum intermolecular distance between LC3B monomers during 750 ns atomistic MD simulations. Two independent replicas are shown for each condition. The dashed horizontal line (0.5 nm) indicates the dissociation threshold. Corresponding normalized histograms summarize the distance distributions sampled throughout each trajectory, with dashed vertical lines indicating the mean distance per replica. LC3B^WT^ remains stably associated throughout the simulation, whereas LC3B^Loop6Mutant1^ and LC3B^Loop6Mutant2^ exhibit increased intermonomer separation and frequent sampling of dissociated states, consistent with destabilization of the Loop 6-mediated clustering interface. Source data are available online for this figure.

To understand the specific contributions of polarity and hydrophobic interactions driving LC3 cluster formation, we systematically introduced mutations to alter the clustering binding pocket. A total of four mutants were generated (Fig. 4A). In LC3^Loop6Mutant1^, we increased the polarity of the Loop6 region by substituting the hydrophobic residues with serine; namely, three substitutions were made (M88S, V89S, and V91S). In LC3^Loop6Mutant2^, we focused on serine and altered the hydrophobicity within Loop6 (S87A, S90A, S92A, T93A, and P94A). Furthermore, we designed two negative control mutants (LC3^Loop3Mutant^ and LC3^C-terMutant^) that may interfere non-specifically to disrupt regions peripheral to the specific clustering sites, ensuring minimal disturbance to the clustering interface (Appendix Fig. S18). Further, to understand their impact, we performed four mutant dimer simulations in atomistic detail to fully capture sidechain dynamics that cannot be resolved in coarse-grained simulations. As opposed to the coarse-grain simulations, an atomistic scale was required for understanding residue-level sidechain packing and polarity changes at the interface. To assess the reproducibility of our findings, each simulation condition was performed in duplicate, yielding two independent replicas per Loop 6 mutant and for the wild-type dimer.

In all simulations, the minimum intermolecular distance between the two LC3B monomers was monitored as a direct readout of interface integrity (Fig. 4B,C). The wild-type dimer retained a stable, compact interface throughout both replicas, with the minimum intermolecular distance remaining consistently below 0.5 nm across the full 750 ns trajectory (mean ≈  0.17–0.19 nm; Fig. 4C). In contrast, both Loop 6 mutants exhibited rapid interface destabilization, with the intermolecular distance rising to values exceeding 1.0 nm, indicating complete loss of dimer contact. The reproducibility of the dissociation behavior across independent replicas in both mutants confirms that the observed instability reflects that the mutations abrogate the intermolecular interface and promote dimer dissociation. In contrast, the negative control mutants at Loop 3 and the C-terminus maintained their clustered conformation throughout the simulations, exhibiting only subtle changes in the relative orientation of the dimer interface (Appendix Fig. S18). Together, these findings demonstrate how residue-specific polarity and hydrophobic interactions within the Loop6 region govern the structural integrity and persistence of LC3 clusters.

### STORM imaging of LC3 mutants shows an altered clustering pattern and impaired motility

**Figure 5:**
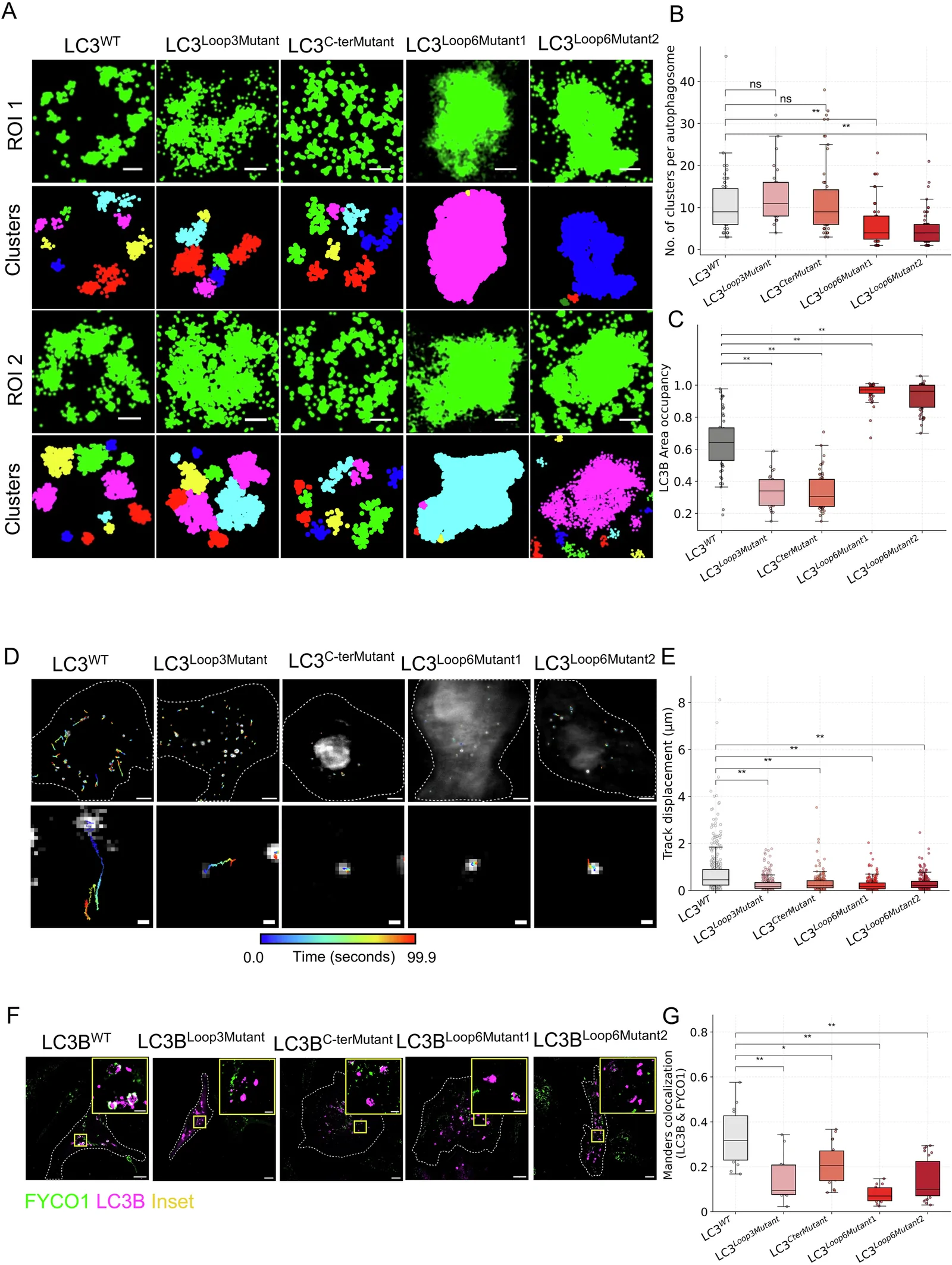
Super-resolution STORM images and cluster analysis of LC3B wild-type and mutants. (**A**) Representative STORM images of individual autophagosomes from starved BS-C-1 cells transiently expressing LC3B^WT^ or the indicated mutants. For each, two representative regions of interest (ROIs) and corresponding DBSCAN-based segmentation are shown. Segmented nanoclusters are pseudo-colored to distinguish individual clusters. (**B**,** C**) Quantification of LC3 nanocluster organization on autophagosome. (**B**) Box plot showing the number of LC3B clusters per autophagosome (LC3B^WT^ vs LC3B^Loop3Mutant^, *P* = 0.096; vs LC3B^C-terMutant^, *P* = 0.786; vs LC3B^Loop6Mutant1^, *P* = 9.05 × 10⁻⁶; vs LC3B^Loop6Mutant2^, *P* = 3.78 × 10⁻⁸). (**C**) Box plots showing the fraction of autophagosomal area occupied by LC3B clusters (LC3B^WT^ vs LC3B^Loop3Mutant^, *P* = 2.88 × 10^−^¹⁰; vs LC3B^C-terMutant^, *P* = 3.14 × 10^−^¹³; vs LC3B^Loop6Mutant1^, *P* = 1.01 × 10^−^¹⁵; vs LC3B^Loop6Mutant2^, *P* = 1.97 × 10^−^¹³). Data were obtained from 6–8 cells from two independent experiments. (**D**) Representative single-particle tracking trajectories (colored lines) of EGFP–LC3B–positive autophagosomes in BS-C-1 cells (20 fps). The lower panel represents a magnified view of a representative single autophagosome trajectory. Tracks are color-coded according to the time (T). (**E**) Box plot showing the track displacement for the wild-type and mutant LC3 (LC3B^WT^ vs LC3B^Loop3Mutant^, *P* = 1.01 × 10⁻²⁷; vs LC3B^C-terMutant^, *P* = 3.28 × 10⁻¹⁸; vs LC3B^Loop6Mutant1^, *P* = 2.14 × 10⁻²⁹; vs LC3B^Loop6Mutant2^, *P* = 5.78 × 10⁻¹⁹). Data were obtained from 11–13 cells from three independent experiments. (**F**) Representative dual-color confocal images of LC3B^WT and mutants (magenta) with FYCO1 (green). Yellow boxes indicate magnified regions in the corresponding insets. (**G**) Quantification of Mander’s colocalization coefficient between LC3B and FYCO1 (LC3B^WT^ vs LC3B^Loop3Mutant^, *P* = 4.14 × 10⁻⁴; vs LC3B^C-terMutant^, *P* = 8.62 × 10⁻³; vs LC3B^Loop6Mutant1^, *P* = 1.53 × 10⁻⁶; vs LC3B^Loop6Mutant2^, *P* = 7.43 × 10⁻⁵). The data represents LC3B^WT^ *n* = 16 cells; LC3B^Loop3Mutant^, *n* = 13 cells; LC3B^C-terMutant^, *n* = 20 cells; LC3B^Loop6Mutant1^, *n* = 17 cells; LC3B^Loop6Mutant2^, *n* = 24 cells from two independent experiments. Scale bar: 200 nm for (**A**), 5 μm for (**D**) and 500 nm for the corresponding zoom, 10 µm for (**F**) and 2 µm for the zoomed inset in (**F**). For all box plots (**B**, **C**, **E**, **G**), the center line represents the median, box limits indicate the 25th and 75th percentiles (interquartile range, IQR), and whiskers extend to the most extreme data points within 1.5 × IQR of the box limits. All individual data points are overlaid; minimum and maximum values are represented by these overlaid points. The statistical significance was determined using a two-sided Mann–Whitney *U*-test. ns, *P* > 0.05; *P ≤ 0.05; **P ≤ 0.005. Source data are available online for this figure.

From the above molecular dynamics simulations, we obtained dynamic consequences of mutations introduced in the clustering interface. To experimentally validate these mutations and understand their clustering behavior on the autophagosome, four mutant constructs were generated. Western blotting of GFP–LC3B^WT^ and mutant constructs revealed both LC3-I and LC3-II bands, indicating that all mutants retained their ability to conjugate to the membrane (Appendix Fig. S19). Each LC3 mutant variant was tagged with EGFP at the N-terminal to distinguish the mutant populations from the endogenous population when expressed in the cells. To specifically understand the impact of mutations on the formation of nanoclusters, we transiently overexpressed LC3 wild-type and mutant variants tagged with EGFP in BS-C-1 cells, starved them, immunostained for EGFP and performed STORM imaging followed by DBSCAN-based cluster analysis (Fig. 5A). Importantly, expression of GFP-LC3B variants did not substantially alter endogenous LC3B or GABARAP protein levels, with only a modest reduction in endogenous LC3 observed for the LC3^**Loop3Mutant**^ (Appendix Fig. S19). Our positive control is wild-type EGFP-LC3 that showed similar cluster parameters as that of the endogenous population, while the two loop 6 mutants shows no proper clustering (LC3^Loop6Mutant1^ and LC3^Loop6Mutant2^), where LC3 appears to be evenly distributed on the autophagosome (Fig. 5B,C). Hence, loop 6 is one of the crucial regions for regulating the LC3 cluster property. Interestingly, the typical spatially uncoupled LC3 cluster pattern observed in wild-type LC3 are notably distinct in the LC3 loop 6 variants. We quantified the percentage of autophagosome area occupied by LC3, to observe that unlike the wild-type LC3, which forms nanoclusters concentrated on the autophagosome with 42% occupancy, the loop 6 mutants exhibit a single, blob-like assembly on the membrane, covering ~80% of the autophagosomal surface. Furthermore, the two negative controls showed relatively less alteration in the clustering pattern with a shift in occupancy. The area of the autophagosome occupied by LC3 clusters decreased by half in loop 3 and c-terminal mutant compared to the wild-type (Fig. 5C), suggesting that the clusters formed are of smaller size. To examine whether altered LC3 clustering affects lipid organization, we performed sequential two-color STORM imaging of LC3^Loop6Mutant2^ and PI(3)P (Appendix Fig. S20). Unlike LC3B^WT^, where LC3 nanoclusters substantially overlap with PI(3)P-enriched nanodomains, LC3^Loop6Mutant2^ assemblies showed markedly reduced spatial co-clustering with PI(3)P. These observations raise the possibility that protein–lipid co-organization contributes to the functional integrity of the LC3 assembly platform.

Newly formed and matured autophagosomes, guided by motor proteins, travel along microtubules to eventually fuse with lysosomes, forming autolysosomes (Yu et al, 2017; Yim and Mizushima, 2020). Several studies have demonstrated that LC3 regulates the adapter proteins, FYCO1 and JIP1, which are necessary for recruiting and activating kinesin and dynein motors, respectively, on autophagosomes, thereby controlling the bidirectional long-range transport of autophagosomes within the cells (Pankiv et al, 2010; Fu et al, 2014). To determine if LC3 oligomerization on the autophagosome membrane is essential for LC3 function, we investigated the motility of autophagosomes with varying levels of LC3 clustering induced by LC3 mutants. To this end, we performed live-cell imaging of stareved BS-C-1 cells transfected with wild-type and mutant EGFP-LC3 variants using TIRF microscopy (Fig. 5D). Single-particle trajectory analysis of individual autophagosomes from live-cell imaging reveals that LC3 mutations lead to confined motion of the autophagosome, unlike the long-range directed transport observed with wild-type LC3 (Fig. 5D lower panel, Movies EV5–EV9). The track displacement analysis shows that autophagosomes decorated with wild-type LC3 exhibited long-distance movement in a typical directed manner, whereas those with mutant LC3 displayed hindered motility as seen by a decrease in net displacement (Fig. 5E). Additionally, we utilize Neuro2A cells, which develop long neurite-like projections in culture and thus provide extended tracks for motility analyses. Using TIRF imaging and kymograph analysis, we observed that autophagosomes labeled with LC3^WT^ constructs exhibited long-range movement. In contrast, the mutant LC3 constructs displayed impaired motility even in cells with extended projections (Movies EV10–EV15). We performed semi-automated single-particle tracking analysis to segment trajectories into directed and confined motion states (see methods), and quantified the instantaneous run length, speed and pause duration of the tracks. This analysis revealed a significant reduction in instantaneous run length and an increase in pause duration in mutant tracks, while the speed remained unchanged (Appendix Fig. S21; Movie EV16). These findings suggest that the motility defects we report are robust and not limited by cell type. Furthermore, immunofluorescence analysis of FYCO1 with LC3^WT^ and mutants revealed a significant reduction in FYCO1–LC3 colocalization for all mutants as compared to WT. These results suggest that decreased FYCO1 recruitment potentially contributes to the reduced motility of autophagosomes observed with the impaired LC3 variants. Our results demonstrate that the recruitment of the kinesin adapter FYCO1 to autophagosomes is reduced in cells expressing mutant LC3 compared to cells expressing wild-type LC3 (Fig. 5G). Consistent with the structural separation of the clustering interface and LIR-binding pocket, LC3 clustering mutants retained FYCO1 binding with only minor changes in association (Appendix Fig. S22). These findings suggest that the reduced motility phenotype is unlikely to result primarily from impaired FYCO1–LC3 interactions. Instead, the clustering-interface mutants appear to affect higher-order LC3 organization and membrane-associated assembly properties rather than directly perturbing LIR-mediated FYCO1 recognition.

Since autophagosome motility was compromised for the LC3^Loop6Mutant1^ and LC3^Loop6Mutant2^, which does not form LC3 clusters, as well as for the LC3^CterMutant^ and LC3^Loop3Mutant^, which forms smaller LC3 clusters, we conclude that an optimal LC3 cluster size and distribution are necessary for functional autophagosome transport. Interestingly, we also observe a remarkable decrease in autophagosome size with the mutant LC3 expression (Appendix Fig. S22). These results suggest a functional role of LC3 clustering in autophagosome formation.

#### Disruption of LC3 nanoclusters hampers intracellular bacterial clearance

**Figure 6:**
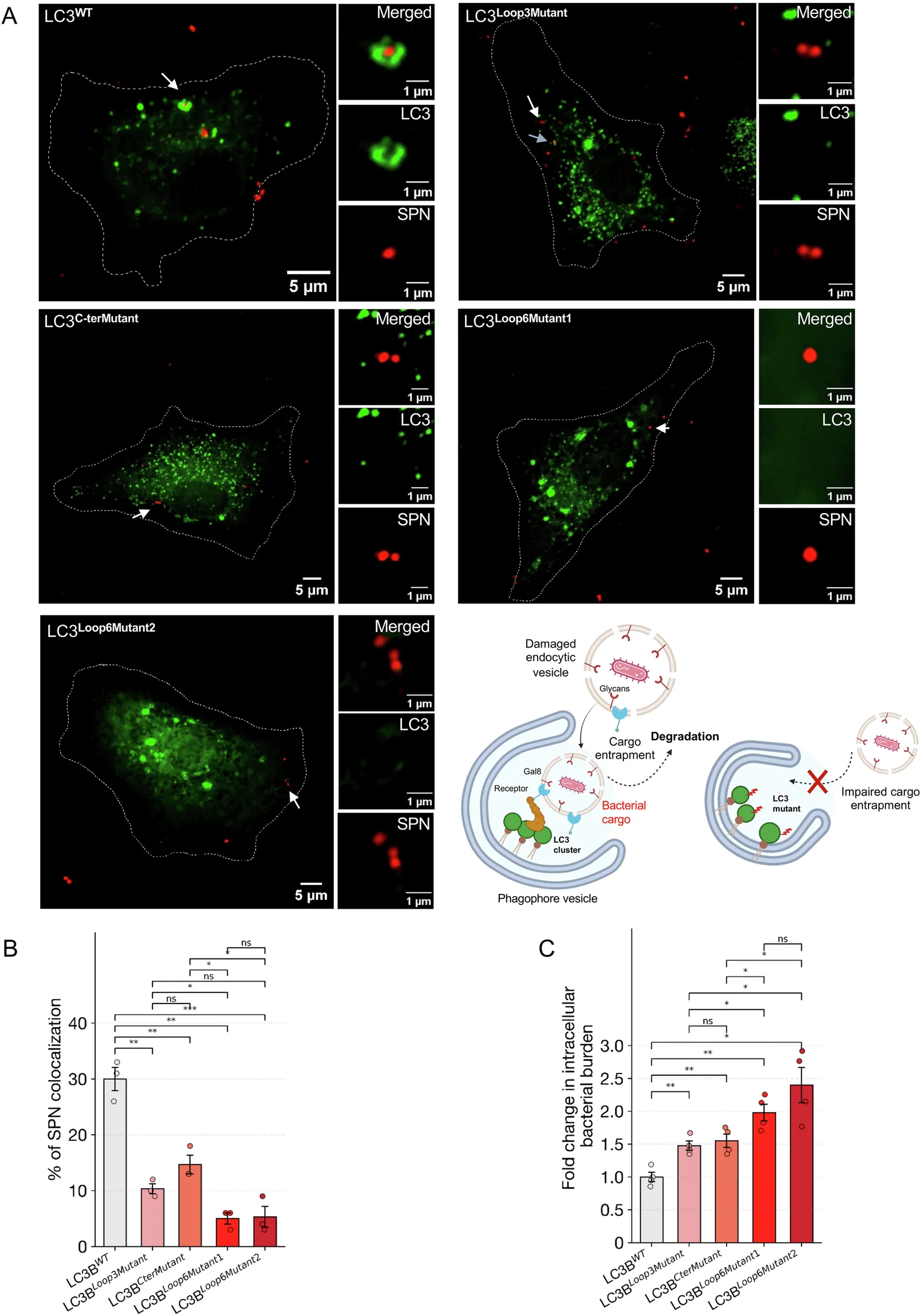
LC3 nanoclusters are required for efficient intracellular bacterial clearance. (**A**) Confocal microscopy demonstrating the entrapment of SPN (red) by an autophagosome marked by GFP-LC3 (WT) (green), compared to GFP-LC3 mutants, which had compromised SPN colocalization in host A549 cells. White arrows mark the regions of interest (ROIs) shown in the corresponding magnified insets. The accompanying schematic illustrates efficient bacterial cargo encapsulation in wild-type LC3, whereas mutant LC3 fails to form proper nanoclusters, resulting in incomplete cargo entrapment. (**B**) Graph depicting the percentage of colocalization of SPN with LC3. 100 bacteria per coverslip. Data were represented as mean ± SD from 3 independent biological replicates (LC3B^WT^ vs LC3B^Loop3Mutant^, *P* = 4.78 × 10⁻³; vs LC3B^C-terMutant^, *P* = 5.23 × 10⁻³; vs LC3B^Loop6Mutant1^, *P* = 2.03 × 10⁻³; vs LC3B^Loop6Mutant2^, *P* = 9.57 × 10⁻⁴). (**C**) Fold change intracellular bacterial burden of SPN in cells expressing LC3 mutants compared to host cells expressing wild-type LC3B^WT^. Data were represented as mean ± SD from 4 independent biological replicates (LC3B^WT^ vs LC3B^Loop3Mutant^, *P* = 2.87 × 10⁻³; vs LC3B^C-terMutant^, *P* = 5.11 × 10⁻³; vs LC3B^Loop6Mutant1^, *P* = 1.47 × 10⁻³; vs LC3B^Loop6Mutant2^, *P* = 0.011). Statistical significance for (**B**, **C**) was determined using a two-sided Welch’s *t*-test (parametric). ns, *P* > 0.05; *P ≤ 0.05; **P ≤ 0.005;***P ≤ 0.001. Source data are available online for this figure.

*Streptococcus pneumoniae* (SPN), a gram-positive bacterium, transiently invades the host cells to either establish an alternative niche or disseminate into the bloodstream, leading to sepsis (Weiser et al, 2018; Ercoli et al, 2018). As a countermeasure, host cells employ autophagy as a key degradative mechanism to eliminate the threat (Ogawa et al, 2018; Surve et al, 2018). Upon entry into host cells, SPN is initially enclosed in an endosome, which it ruptures using its cholesterol-dependent pore-forming toxin, pneumolysin, thus impairing its fusion with the lysosomes (Surve et al, 2018; Shewell et al, 2014). Consequently, these damaged endosomes are recognized by the autophagy machinery either through galectin-8 or ubiquitin-mediated sensing, leading to the recruitment of autophagic receptors, allowing the capture of bacteria-containing vacuoles (BCVs) by the autophagosomes, eventually fusing with the lysosomes for degradation (Surve et al, 2018; Santra et al, 2024; Thurston et al, 2012). We wondered if the nanoclustering of LC3B is required for proper capture and engulfment of these BCVs. Using A549 host cells expressing four LC3B mutants, confocal microscopy revealed that GFP–LC3^WT^ efficiently entrapped SPN within autophagosomes, whereas the mutants showed significantly reduced SPN entrapment (Fig. 6A). Specifically, a 2–3-fold reduction in SPN association was observed for the LC3^Loop3Mutant^ and LC3^CterMutant^ mutants. Notably, the loop 6 mutants (LC3^Loop6Mutant1^ and LC3^Loop6Mutant2^) exhibited a more pronounced ~6-fold decrease in bacterial association compared to LC3^WT^ (Fig. 6B). To assess the consequence of this impaired association, we quantified the intracellular bacterial burden following SPN infection in these mutant-expressing cells. We found loop 3 and c-terminal mutants showed a modest elevation in intracellular SPN burden, while both the loop 6 mutants displayed a significant ~2–2.4-fold increase in bacterial load (Fig. 6C). Further, all LC3 clustering mutants exhibited p62 accumulation in response to Bafilomycin A1 treatment, indicating ongoing autophagic flux. Although LC3^Loop6Mutant1^ and LC3^Loop6Mutant2^ showed modestly increased p62 accumulation relative to LC3^WT^, the overall differences were small, suggesting that disruption of LC3 nanoclustering does not substantially impair bulk autophagosome turnover (Appendix Fig. S23). To understand if the cargo-capturing defect observed in LC3B mutants is indeed due to impaired nanocluster formation and not a result of disrupted interaction with core autophagy machinery, we performed immunoprecipitation to assess binding of mutant to ATG4, ATG3 and ATG7, which are involved in LC3B activation and lipidation (Ye et al, 2023). Compared with wild-type LC3B, the Loop 6 and Loop 3 mutants exhibited largely unchanged interactions with key autophagy factors (Appendix Fig. S24). In contrast, the C-terminal mutant displayed altered interactions, which is consistent with its proximity to the LC3 lipidation and conjugation interface. These observations suggest that, with the exception of the C-terminal mutant, the observed functional defects are not primarily driven by impaired conjugation or loss of canonical binding interactions. Instead, they are more directly associated with the disruption of LC3 nanoscale organization and self-assembly on autophagosomal membranes. Together, these findings indicate that the ability of LC3B to form nanocluster assemblies, particularly through interactions mediated by the Loop 6 region, is important for efficient recognition and clearance of SPN from the host cytosol.

## Discussion

The dynamic assembly of autophagosomes relies on reversible and transient interactions involving lipid-anchored LC3, densely packed on these double-membraned vesicles. The present understanding of LC3 has transitioned from a molecular switch oscillating between cytosolic LC3-I and membrane-associated LC3-II to being recognized as a key facilitator in the autophagic process. Moreover, highly crowded environments can give rise to numerous transient and non-specific interactions, making it difficult to deconvolute specific association mechanisms from background intermolecular contacts (Rivas and Minton, 2016). The previous report has indicated that ATG8 homolog forms high molecular weight complexes upon conjugation with phosphatidylethanolamine (PE) (Nakatogawa et al, 2007) and prompted us to look into molecular self-assembly in greater detail.

In this work, we show that LC3B forms functional nanoclusters on autophagosome membranes, packing optimally in spatially isolated islands. Using a combined computational and experimental approach, we demonstrate that LC3 proteins assemble into nanometer-sized clusters optimally distributed on autophagosomes. Simulations also provided us with the cue that there exists specific PI lipid enrichment on these clusters. STORM imaging of PI(3)P revealed a clear overlap with LC3 clusters, confirming the association between them. Molecular analysis revealed a novel clustering interface on LC3 proteins, characterized by an alternating polar and hydrophobic residue, which drives clustering. To disrupt this pattern, we generated four mutants: the first two targeting the Loop6 region (LC3^Loop6Mutant1^ and LC3^Loop6Mutant2^), and the other two designed to minimally interfere with clustering (LC3^Loop3Mutant^, LC3^CterMutant^). Extensive experimental and in silico mutant characterization showed that disrupting the polarity and hydrophobicity of this pocket destabilizes the clusters, reducing their stickiness and impairing their stability. The heterogeneous LC3B interfaces resemble the degenerate ubiquitin–ubiquitin interactions reported to drive FAM134B nanoclustering, suggesting that multi-modal distributed contacts may be a common feature of autophagy-related assemblies (González et al, 2023).

By elucidating the molecular determinants of LC3 oligomerization on the membrane, our findings shed light on the role of LC3 in the spatiotemporal regulation of autophagy. Several biological scenarios can be speculated, where LC3-enriched islands may serve as critical signaling platforms. The most plausible scenario is where LC3 clusters might be involved in the regulation of membrane dynamics, controlling the size and shape of autophagosomes. STORM imaging of all four mutants revealed smaller autophagosomes with disrupted cluster formation. While LC3^Loop6Mutant1^ and LC3^Loop6Mutant2^ drastically altered the appearance of LC3 proteins, causing them to diffuse throughout the vesicle, the other two mutants showed only smaller clusters. It is possible that the heterogeneous clustering interface observed in our loop 6 interface 2, even when present in low abundance, is sensitive to control the size of the autophagosome. While our simulation–experimental framework provides molecular-level insights into LC3 self-association, the clusters observed in simulations and experiments represent different physical size scales. For the simulated membrane patch (~100 × 100 nm²), with an initial configuration containing ~90 LC3 molecules, corresponding to a local surface density of ~9 × 10⁻³ molecules/nm². The extrapolation of this local simulation density is therefore not directly comparable to larger membrane-associated nanocluster assemblies observed experimentally. Importantly, the coarse-grained simulations were not intended to directly reproduce clusters at the supramolecular scales observed experimentally but were specifically designed to probe the underlying molecular interactions and weak multivalent interfaces that drive local LC3 self-association on membranes. In complementary experiments, STORM imaging revealed membrane-associated LC3 assemblies predominantly in the size range of ~150–300 nm, with the number of such assemblies increasing during autophagosome maturation. However, extracting precise molecular stoichiometries from STORM-resolved assemblies remains technically challenging. Further, in terms of quantitative protein stoichiometry, previous estimates in yeast suggest that autophagosomes contain on the order of a few hundred Atg8 molecules, although local membrane densities can vary substantially during phagophore expansion and maturation (Geng et al, 2008; Xie et al, 2009). Previous super-resolution studies have similarly shown that autophagy initiation proteins form nanoscale assemblies with defined oligomeric states during autophagosome biogenesis. For example, quantitative PALM imaging of ULK1 demonstrated starvation-induced higher-order nanoscopic assemblies associated with Atg13, LC3B, and other autophagy regulators during early autophagosome formation (Banerjee et al, 2023). Together with our observations, these findings support the broader concept that nanoscale organization is an important feature of autophagy machinery assembly.

Findings from different experiments also show that LC3 plays an essential role in membrane dynamics (Xie et al, 2008; Weidberg et al, 2011), interacting with other autophagy-related proteins to aid in the elongation and closure of the phagophore (Galluzzi et al, 2017). Wild-type LC3-positive autophagosomes showed a robust bidirectional movement, while all LC3 mutants displayed impaired motility, evident from a decrease in net displacement of autophagosomes. We indeed observe the dominant-negative behavior of LC3B clustering mutants. Since LC3-mediated self-assembly appears to be important for generating locally concentrated interaction platforms, the incorporation of clustering-defective mutants into these assemblies likely disrupts the spatial organization and collective behavior of endogenous LC3 molecules. Consequently, these mutants might interfere with the formation and higher-order organization of LC3 wild-type nanoclusters on autophagosomal membranes. Additionally, we saw a significant decrease in FYCO1 recruitment on LC3 mutants as compared to LC3^WT^. Since FYCO1 interacts with LC3 through its LIR motif, and the canonical HP1 and HP2 pocket sites on LC3 remain intact in our mutants, we speculate that the impaired FYCO1 recruitment arises from defects in LC3 nanoclustering rather than disruption of the LIR–LC3 interaction itself.

The overlap between LC3B assemblies and PI(3)P-enriched membrane regions is particularly interesting in the broader context of lipid-mediated organization during autophagosome biogenesis. Multiple autophagy regulators, including WIPI proteins, Atg13, ATG17, ATG2, and ATG9-associated vesicles, are known to directly sense, bind, remodel, or transport specific membrane lipids during phagophore initiation and expansion (Nishimura and Tooze, 2020; Ragusa et al, 2012; Sawa-Makarska et al, 2020; Proikas-Cezanne et al, 2015). In particular, PI(3)P-enriched membrane domains act as key signaling platforms for recruitment of WIPI proteins and downstream autophagy machinery through PI(3)P-recognition modules such as FYVE domains (Dooley et al, 2014). Our observations suggest that LC3B nanoclusters may similarly emerge preferentially within phosphoinositide-enriched membrane environments, thereby coupling local membrane composition with higher-order LC3 organization. Interestingly, the altered spatial organization observed for clustering-defective LC3 mutants raises the possibility that protein–lipid co-clustering itself may represent an important organizational principle during autophagosome maturation, where local enrichment of both LC3 assemblies and specific phosphoinositide species cooperatively stabilizes membrane-associated reaction hubs. Such coordinated protein–lipid nanoscale organizations could locally concentrate cargo receptors, membrane remodeling proteins, and autophagy factors to spatially regulate cargo sequestration and membrane expansion. More broadly, these findings support a model in which autophagosome biogenesis is regulated not only through protein–protein interactions, but also through dynamic protein–lipid co-organization on specialized membrane domains.

Also, key studies have highlighted how nanometer-scale assemblies of peripheral membrane proteins like lipid-anchored small GTPase Ras proteins serve as platforms for membrane curvature, vesicle formation, and trafficking (Parton, 2004). Such membrane-bound proteins form transient signaling platforms that self-organize, often driven by lipid-lipid interactions (Sharma et al, 2004), protein–lipid affinity (Zhou et al, 2017), or external stimuli (Zhou and Hancock, 2023), with recent structural work resolving the specific molecular contacts that stabilize such membrane protein clusters (Mandala et al, 2025). However, LC3 nanoclusters may also spatially and temporally control cargo degradation and extend the role of peripheral proteins that are residing on plasma membranes. These observations prompted us to further examine the role of LC3B nanoclustering in selective antibacterial autophagy. Strikingly, disruption of LC3B nanoclustering markedly impaired autophagic sequestration of *Streptococcus pneumoniae*, with clustering-defective mutants exhibiting substantially reduced bacterial capture. Consequently, cells expressing these mutants displayed significantly higher intracellular bacterial loads, indicating that LC3B self-assembly is important for efficient recognition and clearance of invasive bacterial cargo. These defects are also consistent with the impaired autophagosome size and altered membrane organization observed in cells expressing clustering-defective LC3 mutants.

However, notable differences were observed among the individual mutants, indicating that distinct interfaces contribute differently to LC3 assembly and function. Our structural and clustering analyses suggested that the Loop 6 region forms the principal LC3 self-association interface, whereas the Loop 3 and C-terminal regions contribute more peripheral contacts within the higher-order assembly network. Consistent with this model, approximately 60% of the identified clusters involved Loop 6-mediated interactions, whereas only ~8% involved the C-terminal interface. Accordingly, mutations in the C-terminal region produced only a partial reduction in clustering and retained the ability to form smaller membrane-associated assemblies, while disruption of Loop 6 largely abolished LC3 self-association and resulted in the diffuse membrane distribution observed by super-resolution imaging. The functional consequences mirrored this hierarchy of assembly defects, i.e., the LC3^CterMutant^ retains ~15% SPN colocalization compared to ~30% for LC3B^WT^, whereas the Loop 6 mutants show only ~5–6% colocalization. Notably, LC3^Loop6Mutant2^ also exhibited substantially reduced co-clustering with PI(3)P-enriched membrane domains compared with LC3^WT^, suggesting that disruption of the primary self-association interface additionally perturbs lipid-associated organization.

Our study also has certain limitations. First, although our simulations identify the Loop6 interface, the precise stoichiometry and kinetics of nanocluster assembly remain to be established, as these depend on the topology of the clusters on the inner and outer membranes of the autophagosome. Second, we cannot fully exclude the contribution of endogenous LC3/GABARAP-family members to the observed phenotypes. Finally, while we observe a strong association between LC3 nanoclusters and PI3P-enriched membrane domains, the broader involvement of the autophagic machinery in organizing these PI3P–LC3 clusters, and whether other factors spatially co-cluster within these domains, remains to be examined.

Together, these findings provide important mechanistic insight into how higher-order LC3 organizations contribute to autophagy. Our results suggest that LC3 nanocluster assemblies are not merely passive organizational byproducts of membrane recruitment, but active membrane-associated structures that facilitate autophagosome maturation, intracellular transport, and selective cargo clearance. More broadly, this work establishes LC3 self-assembly as an important layer of autophagosomal organization and highlights the role of mesoscale protein assembly in regulating autophagy function.

## Methods

**Table:** Reagents and tools table

Reagent/resource	Reference or source	Identifier or catalog number
**Experimental models**		
BS-C-1 cells (African green monkey, *Cercopithecus aethiops*, kidney epithelial)	A Kind gift from Prof. Melike Lakadamyali, University of Pennsylvania, US.	
Neuro-2a cells (mouse neuroblastoma)	A Kind gift from Prof. Dharmaraja Allimuthu, Department of Chemistry, IIT Kanpur, India.	
A549 cells (human lung alveolar carcinoma, type II pneumocyte)	American Type Culture Collection (ATCC)	CCL-185
*Streptococcus pneumoniae* strain R6 (serotype 2, unencapsulated)	American Type Culture Collection (ATCC)	BAA-255
**Recombinant DNA**		
pcDNA3.1-GFP-LC3B-WT	This study	Custom made
pcDNA3.1-GFP-LC3B-Loop3Mutant (L44H, V46T, L47S)	This study	Custom made
pcDNA3.1-GFP-LC3B-CterMutant (Q116A, E117A, T118V)	This study	Custom made
pcDNA3.1-GFP-LC3B-Loop6Mutant1 (M88S, V89S, V91S)	This study	Custom made
pcDNA3.1-GFP-LC3B-Loop6Mutant2 (S87A, S90A, S92A, T93A, P94A)	This study	Custom made
pmCherry-2xFYVE	Addgene	#140050
p40PX-EGFP	Addgene	#19010
**Antibodies**		
Rabbit anti-LC3B (immunofluorescence)	Cell Signaling Technology (CST)	#3868
Rabbit anti-LC3B, clone E5Q2K (immunofluorescence)	Cell Signaling Technology (CST)	#83506
Chicken anti-mCherry	Novus Biologicals	NBP2-25158
ChromoTek GFP-Booster Alexa Fluor 647	ChromoTek	gb2AF647
Goat anti-rabbit IgG-Alexa Fluor 647 (secondary)	Jackson ImmunoResearch	711-005-152
Goat anti-chicken IgG-Alexa Fluor 647 (secondary)	Jackson ImmunoResearch	703-005-155
Anti-WIPI2	Abcam	AB105459
Anti-LAMP2	Santa Cruz Biotechnology	sc-18822
Anti-FYCO1	Abnova	H00079443-A01
FYCO1 antibody	Invitrogen	PA5-45805
Anti-ATG16L1	Cell Signaling Technology (CST)	#8089
Anti-ATG13	Cell Signaling Technology (CST)	#13468
Anti-Enolase	A Kind gift from S. Hammerschmidt, University of Greifswald, Germany	
Anti-ATG3 (immunoprecipitation/western blot)	Cell Signaling Technology (CST)	3415S
Anti-ATG4B (immunoprecipitation/western blot)	Cell Signaling Technology (CST)	5299S
Anti-ATG7 (immunoprecipitation/western blot)	Cell Signaling Technology (CST)	8558S
Anti-LC3B (immunoprecipitation)	Sigma-Aldrich	L7453
Anti-GFP (western blot primary antibody)	Abcam	ab13970
HRP-conjugated secondary antibody (western blot)	Invitrogen	A16054
**Oligonucleotides and other sequence-based reagents**		
**Chemicals, enzymes and other reagents**		
Fetal bovine serum (FBS)	Hyclone	SH30071.03
Fetal bovine serum (FBS)	Gibco	16000-044
Penicillin-streptomycin	Invitrogen	15140122
Antibiotic-antimycotic	Gibco	15240062
DMEM, high glucose	Gibco	11965092
DMEM	HiMedia	AT007
XtremeGene HP DNA Transfection Reagent	Roche	6366236001
Neon NxT Electroporation System (transfection kit)	Invitrogen	NEON18SK
VectaShield mounting medium (without DAPI)	Vector Laboratories	H-1000-10
Alexa Fluor 647 dye	Invitrogen	A20006
Alexa Fluor 405 (activator dye)	Invitrogen	A30000
TetraSpeck microspheres (fiducial markers)	Invitrogen	T7279
Protease inhibitor cocktail	Promega	G6521
Phosphatase inhibitor cocktail	GBioSciences	#786-870
PVDF membrane, Immobilon-FL, 0.45 µm	Millipore	IPVH08100
Protein G Agarose beads	HiMedia	DS0818
Clarity Max Western ECL Substrate	Bio-Rad	#1705062
Bafilomycin A1	Sigma-Aldrich	B1793
Todd-Hewitt broth (THB)	HiMedia	M313
Yeast extract	HiMedia	RM027
Sodium borohydride	Invitrogen	013432.57
**Software**		
GROMACS, version 2020	GROMACS development team (open source)	www.gromacs.org
MARTINI force field, version 2.2	Marrink lab (open source)	cgmartini.nl
CHARMM36m force field (2019 version)	MacKerell lab (open source)	mackerell.umaryland.edu
PyMOL	Schrödinger	
ChimeraX	UCSF Resource for Biocomputing, Visualization, and Informatics	
MDAnalysis	MDAnalysis (open source)	www.mdanalysis.org
NumPy	NumPy (open source)	numpy.org
LiPyphilic	Smith & Lorenz (open source)	github.com/p-j-smith/lipyphilic
insane.py (membrane-building script)	MARTINI website	cgmartini.nl
martinize script (modified in-house for the GLP residue)	MARTINI website, modified in this study	cgmartini.nl
backward.py (coarse-grained to atomistic backmapping script)	MARTINI website modified in this study	cgmartini.nl
POVME 3.0	Amaro lab (open source)	
Fiji (ImageJ)	Fiji (open source)	fiji.sc
TrackMate (Fiji plugin)	Fiji (open source)	fiji.sc
Zen Lite, version 5.0	Zeiss	
Image Lab software	Bio-Rad	
NEO/NEO_analysis software	Abbelight	
Origin 2022b	OriginLab	
MATLAB (custom script for active/passive transport classification)	MathWorks	
Insight3	Kind gift from B. Huang, University of California, San Francisco, San Francisco, CA	
**Other**		
Custom-built 2D-STORM microscope	This study (custom-built)	
SAFeRedSTORM module (3D dSTORM)	Abbelight	
Evident/Olympus IX3 microscope	Evident/Olympus	
ORCA-Fusion sCMOS camera	Hamamatsu	
Nikon 100x oil-immersion TIRF-SR objective (NA 1.49)	Nikon	
Laser Scanning Confocal Microscope, LSM 780	Zeiss	
Gel Doc XR+ imaging system	Bio-Rad	
ChemiDoc imaging system	Bio-Rad	

### Parameterization and preparation of lipidated LC3 on the membrane

To investigate the dynamics of lipidated LC3 family proteins, we modeled LC3 conjugated with a phosphoethanolamine (PE) lipid anchor within the membrane. The methodology for attaching the PE anchor to the glycine residue follows previously published protocols (Thukral et al, 2015). For the coarse-grained (CG) model parametrization of the modified residue, glycine linked with PE (GLP), parameters were derived from the existing MARTINI force field for 1-palmitoyl-2-oleoyl-sn-phosphoethanolamine (POPE). The atomistic structure was converted into a CG model using the MARTINI force field and the martinize script, obtained from the MARTINI website. This script was modified to incorporate the parameters and bead definitions for the GLP residue. The protein structure was defined using standard MARTINI CG parameters, complemented by an ElNeDyn elastic network model to maintain structural integrity during simulations (Marrink et al, 2007; Periole et al, 2009).

### Generation of the heterogenous membrane model

A mixture of the most prevalent lipids found in the endoplasmic reticulum (ER) was used to mimic a lipid bilayer for depicting the autophagosome membrane (Tooze and Yoshimori, 2010; Fracchiolla et al, 2020). The heterogeneous membrane used in this work is based on ER membrane composition, i.e., DOPC 65%; DOPE 20%; POPI 10%; DOPS 5%, with lipids distributed symmetrically between the inner and outer leaflets. The membrane was generated using the insane.py python script (Wassenaar et al, 2015).

### Starting structure details

The initial structural system for studying the self-assembly of lipidated LC3 on a membrane comprised 90 lipidated LC3 proteins embedded within a 100-nm-wide heterogeneous membrane, designed to mimic the autophagosome environment. The PE anchor attached to the glycine residue of each protein was inserted into the phosphate headgroup region of the membrane to facilitate embedding. To maintain a biologically relevant orientation, the proteins were positioned such that their four previously identified membrane-interacting regions remained in close proximity to the membrane (Lillemeier et al, 2010). The proteins were initially spaced approximately 8 nm apart from one another, ensuring an even distribution while allowing for interactions during the simulation. This spacing corresponds to a local surface density of ~9 × 10⁻³ molecules/nm² and was chosen as a computationally tractable starting condition to permit sufficient lateral diffusion and spontaneous cluster formation within accessible simulation timescales. It does not represent a fixed physiological stoichiometry, which remains experimentally difficult to determine precisely. While experimental estimates in yeast suggest lower average in vivo densities (~1 Atg8 per ~2000 nm²), in vitro reconstitutions demonstrate that locally elevated Atg8 densities are biologically relevant and functionally significant, supporting the use of the density employed here as a tractable approximation for capturing spontaneous cluster formation within accessible simulation timescales (Nakatogawa et al, 2007; Xie et al, 2009; Kaufmann and Wollert, 2014; Geng et al, 2008).

### Protocol for coarse-grained molecular dynamics simulations

CG MD simulations were performed using the MARTINI model (version 2.2) with the MARTINI2.2 force field (De Jong et al, 2013). We first constructed an initial CG model of the lipidated LC3 consisting of 291 beads for 120 residues. DSSP secondary structure assignments were used to generate backbone restraints that preserve local secondary structure for models (Frishman and Argos, 1995). Elastic networks have been applied to maintain the structural fold of the protein while keeping its internal flexibility. A single CG model of lipidated LC3 was embedded into the bilayer spanning the periodic simulation box in the xy plane. The initial configuration and parameters of the CG model of lipidated LC3 were first tested by running a short simulation of 1 μs prior to simulating multiple copies. The resulting system consists of 90 lipidated LC3, with 26,190 beads and 33,280 lipids. Each simulation was performed for 10 μs, and a total of 30 μs of simulation time from three replica runs was generated.

All simulations were performed using Gromacs 2020 and the standard MARTINI protocol (Abraham et al, 2015). Periodic boundary conditions were employed, and three simulations were run with a time step of 20 fs, one with a time step of 10 fs, producing 10 μs of data each. The system was solvated with the standard MARTINI water model using the insane.py script. The system was first minimized and equilibrated using the Berendsen thermostat and barostat, along with position restraints on protein backbone beads, followed by production runs with a 20 fs time step (Berendsen et al, 1995). System temperature and pressure during the production phase were maintained at 310 K and 1 atm with the velocity rescaling thermostat and the semi-isotropic Parrinello–Rahman barostat, respectively (Parrinello and Rahman, 1981). The van der Waals interactions cutoff of 1.2 nm was used with a switching function applied from 0.9 nm in all simulations, and the reaction field coulomb type was utilized with a switching function from 0.0 to 1.2 nm. To constrain the covalent bonds, the LINCS algorithm was applied (Hess et al, 1997).

### Simulation analysis

We conducted clustering analysis using custom Python scripts using essential libraries such as NumPy, MDAnalysis. To identify oligomerization interfaces or sticky patches, we analyzed the coarse-grained interaction density of LC3 proteins. For multimeric LC3 simulations, we calculated the average occupancy, representing the percentage of time frames during which each interacting pair was observed across all simulations. In clustering algorithms, protein interactions were defined based on the proximity of their centers of geometry. Proteins were considered interacting if their centroids were within a 5 nm radius. Since the LC3 protein has an approximate diameter of 5 nm, molecules within this range were treated as potential interactors. Additionally, neighboring proteins were determined to interact when the center of geometry of their residues was within 0.1 nm of each other. This dual metric allowed us to robustly capture interaction dynamics and cluster formation within the simulation environment. To analyse the oligomerized population of LC3 within the 90 chains, we first calculated the distances between the phosphate beads of the GLP residues across all possible chain combinations. Specifically, we examined pairwise distances among all 90 LC3 chains to identify potential interactions. Chains were flagged as interacting when the distance between their phosphate beads was below a critical threshold of 5 nm, which served as the basis for characterizing oligomerization.

To further identify and characterize interaction interfaces, residue occupancy contact maps were generated for all interacting pairs. These maps were constructed using the last 5 μs of the simulation trajectory, offering a high-resolution view of interaction dynamics. A contact between two residues was defined when the distance between them was within 8 Å and the occupancy exceeded 30% of the simulation time. This detailed approach allowed us to precisely map the spatial and temporal features of the LC3B oligomerization interfaces. We combined the contact map matrix from all the generated pairs to construct a cumulative contact map matrix. This cumulative matrix was then normalized by dividing it by the total number of contacts formed.

MDAnalysis tools were extensively utilized for various analyses, such as contact analyses, protein–membrane interactions, and lipid 2D density calculations (Michaud-Agrawal et al, 2011). The LiPyphilic package was employed to determine the enrichment index for each lipid at a cutoff of 0.12 nm (Smith and Lorenz, 2021). Molecular visualizations were generated using PyMOL and ChimeraX.

### All-atomistic simulation protocol for LC3 dimer

The final snapshots from all three simulations, with the clustered population of LC3, were backmapped to all-atom resolution using the *backward.py* MARTINI script (Wassenaar et al, 2014). Each dimer interface was subsequently energy-minimized to resolve any steric clashes. Pocket volumes for each LC3 molecule in the three simulations were calculated using POVME 3.0 on the static backmapped structures (Wagner et al, 2017). The center of geometry was determined separately for the rear side, where Loop6 forms the pocket, as well as the HP1 and HP2 pockets, and used as the reference coordinates for volumetric calculations. A probe size of 0.6 nm and a grid spacing of 0.05 nm were employed to increase the accuracy of the volume measurements.

To investigate the impact of the Loop6-α2 region of the LC3 clustering interface on LC3 self-assembly, four mutants were designed and analyzed. The mutations were introduced in the top-ranked binding poses of the LC3 dimer interface, identified in the CG-MD simulations using PyMOL. The mutated protein structures were subsequently positioned on a membrane composed of heterogeneous lipids. Control simulations of LC3^WT^ in the same dimer orientation were also performed for comparison. Dimer simulations of wild-type (WT) and mutant LC3 structures were prepared using the CHARMM36m force field (2019 version) (Huang et al, 2016), while previously validated parameters were employed for GLP residue, generating a total simulation time of 8 μs. Two simulations were run for 750 ns per replica.

### Cell culture transfection and immunofluorescence

BS-C-1 cells, African green monkey (Cercopithecus aethiops) kidney epithelial cells (CCL-26; American Type Culture Collection) were maintained in a complete growth medium with 10% (v/v) fetal bovine serum (FBS, Hyclone, USA), 2 mM L-glutamine, 1 mM sodium pyruvate, and penicillin-streptomycin (Invitrogen). Neuro-2a cells were maintained in DMEM high glucose (Dulbecco’s Modified Eagle Medium, Gibco, Cat No. 11965092) supplemented with 10% (v/v) fetal bovine serum (FBS, Hyclone, USA), and antibiotic-antimycotic (Gibco). The A549 (human lung alveolar carcinoma, type II pneumocyte; ATCC No. CCL-185) cell lines were maintained in DMEM (Dulbecco’s Modified Eagle Medium, HiMedia, Cat No. AT007) supplemented with 10% fetal bovine serum (FBS, Gibco). Cell culture conditions were maintained at 37 °C with 5% carbon dioxide. Cells were seeded on #1.5H coverslip bottom 35 mm confocal dishes (Ibidi) or eight-well Lab-Tek chambers (Ibidi) for TIRF and STORM imaging. To induce autophagy, cells were starved by incubating in Krebs-Ringer’s solution (pH 7.4) (KRBH), supplemented with 4.5 mM glucose, 0.1% BSA, and 1 mM sodium pyruvate for 2.5 h at 37 °C as described previously (Wagner et al, 2017).

Transient transfection of plasmids was performed using XtremeGene HP DNA transfection reagent (Roche). Briefly, the plasmid DNA and transfection reagent were mixed in serum-free media and kept at room temperature for 20 min. The final volume was adjusted with complete MEM, and the transfection was carried out for 24 h. For transfection using the Neon NxT electroporation system (Invitrogen), A549 cells were washed thoroughly with 1X PBS followed by trypsinization. The cells were washed again with 1X PBS, followed by aliquoting ~3 × 10^6^ cells in a 1.5 ml centrifuge tube. According to the manufacturer’s protocol, these cells were resuspended in R buffer along with 3 μg plasmid DNA. The cell solution was taken in a 100 μl tip (provided by the manufacturer) and was subjected to electroporation at 1200 V for 30 ms with two pulses. The cells were incubated in fresh culture media after 6 h, and experiments were done at least after 16 h of transfection. A list of all the plasmids used in this study is listed in the Reagents and tools table.

For immunofluorescence assays, BS-C-1 and A549 cells were cultured on glass coverslips and fixed with ice-cold absolute methanol at −20 °C (7 min for BS-C-1 cells and 10 min for A549 cells). Following fixation, cells were washed with PBS and blocked for 2 h at room temperature in 3% bovine serum albumin (BSA); BS-C-1 cells were blocked in 3% BSA containing 0.2% saponin in PHEM buffer, whereas A549 cells were blocked in 3% BSA in PBS. Cells were then incubated overnight at 4 °C with the appropriate primary antibodies diluted in blocking buffer (or 1% BSA in PBS for A549 cells), washed with PBS, and subsequently incubated with the corresponding fluorophore-conjugated secondary antibodies for 1 h at room temperature. After final PBS washes, coverslips were mounted using VectaShield® mounting medium without DAPI (Vector Laboratories). A list of all the antibodies and the appropriate dilutions used in this study is listed in the Reagents and tools table.

### STORM imaging protocol

2D Super-resolution STORM images were acquired with a custom-built microscope. To achieve a high signal-to-noise ratio, we used TIRF illumination for the super-resolution STORM imaging experiments. A 647 nm continuous wave visible fiber laser (MPB Communications) was used to excite Alexa Fluor 647 (Invitrogen), while a 405-nm solid-state laser (Obis; Coherent) was used for reactivating the Alexa Fluor 647 (Invitrogen) via an activator dye (Alexa Fluor 405). The emitted light from Alexa Fluor 647 (Invitrogen) was collected by the Nikon 100× oil-immersion TIRF-SR objective (NA 1.49), filtered by an emission filter (ET705/72 m; Chroma), and imaged onto the EM-CCD camera (20 ms per frame), with a total of ~60,000 frames acquired per image. For multicolor STORM imaging, we performed sequential imaging with AF647 dye. We immunostained for LC3 and performed STORM imaging as described above, followed by treatment with sodium borohydride (1 mg/mL) for 7 min at room temperature to permanently quench the AF647 signal from LC3. Next, we performed immunostaining in situ on the microscopic stage for PI(3)P via mCherry antibody using AF647. STORM images of both channels were aligned by matching the localization obtained from the fiduciary markers (TetraSpeck microspheres, Invitrogen T7279)., using a custom image registration software, as reported before (Verdeny-Vilanova et al, 2017). 3D dSTORM imaging was done with a SAFeRedSTORM module (Abbelight) mounted on an Evident/Olympus IX3 microscope having an oil-immersion 100x objective (1.5NA oil immersion, Evident/Olympus) and fiber-coupled 642 nm laser (450 mW Errol). Fluorescent signals of single molecules were collected with an ORCA-Fusion sCMOS camera (Hamamatsu). A cylindrical lens was placed before the camera to introduce astigmatism. Image acquisition and control of microscope were driven by Abbelight’s NEO software. Each image stack contained 60,000 frames. Selected ROI (region of interest) dimension was 512 × 512 pixels (pixel size 97 nm). Cross-correlation was used to correct lateral drifts. Super-resolution images (.tiff) with a pixel size of 10 nm and localization files (.csv) were obtained using NEO_analysis software (Abbelight).

### Cluster analysis

Segmentation of localizations in STORM images was performed using the density-based clustering algorithm DBSCAN (Ester et al, 1996). DBSCAN requires two parameters: the minimum number of points (k) within a search radius (epsilon). A threshold of a minimum of 12 localizations was applied to remove the noise. To determine the epsilon in an unbiased manner, the elbow plot method was used. For cluster analysis, the ROI (region of interest) containing single autophagosomes was cropped. To estimate the size of LC3 clusters identified through DBSCAN analysis, the centroid of each cluster was calculated by taking the geometric mean of all localizations within the cluster. The outer boundary of each cluster was then approximated using a convex hull, which connects the farthest localizations within the cluster (Veeraraghavan and Gourdie, 2016). Subsequently, the distance from the centroid to the convex hull boundary points was measured for each cluster, and the mean of these distances was taken as the cluster radius. This method provides a robust estimation of cluster size by incorporating both the centroid and edge boundaries defined by the spatial distribution of localizations. For comparative analysis of the LC3 clustering between WT and mutants, we enumerated LC3 area occupancy on autophagosome (Cluster area: Total area) and the number of LC3 nanoclusters per autophagosome.

### Single-particle tracking

To perform single-particle tracking analysis of autophagosomes, cells were transfected with equal concentrations of LC3 plasmid constructs for both WT and mutant variants. Following transfection, cells were starved as described previously and subsequently subjected to live-cell imaging using TIRF illumination. We aimed to select cells with a comparable level of LC3 expression. However, we cannot rule out the possibility of variable construct expression contributing to differences in motility parameters. Imaging was performed at a 100 ms time interval for 100 s. Raw data for autophagosome motility were processed by background subtraction using a rolling ball radius of 50.0, followed by bleach correction through histogram matching. The processed data were then analyzed using the TrackMate plugin (Tripathi et al, 2025) in Fiji software using a vesicle diameter of 0.6 μm, the LoG (Laplacian of Gaussian) detector with no initial thresholding, and the Simple LAP tracker with a linking maximum distance of 0.3 μm, a gap-closing maximum distance of 1 μm, a maximum frame gap of 20, and filtering based on the number of spots in each track. Data were exported to Microsoft Excel (2013) and Origin 2022b for further analysis. For single-particle tracking in Neuro2A cells, EGFP–LC3B–positive autophagosomes were tracked using custom-written, semi-automated particle tracking software. The x and y coordinates of the trajectories were obtained by fitting a two-dimensional Gaussian function to the fluorescence intensity point spread function of the objects on each frame. A custom MATLAB script was employed to separate the active and passive transport stages, as described in previous reports (Verdeny-Vilanova et al, 2017; Ershov et al, 2022; Mohan et al, 2019). Briefly, the trajectory analysis employed a four-point sliding window moving along the coordinate data points. A confinement ratio (R) was calculated for each four-point segment by dividing the net distance between the start and end points by the sum of the individual step distances between each point within the segment. As the window advanced point-by-point, individual trajectory points contributed to multiple overlapping segments. The final confinement ratio (R) for each point on the trajectory was estimated as the average ratio of all segments containing that point. While a perfectly linear, directed trajectory yields R = 1, we applied a threshold of *R* > 0.7 to identify directed active transport phases, distinguishing them from confined or diffusive phases (*R* < 0.7). To further refine the classification, an angle-based criterion was implemented: segments that were labeled as passive were those in which consecutive displacement vectors formed angles less than 90°. Furthermore, the classification was validated through mean square displacement (MSD) analysis, which demonstrated an α-value of 1.45 or higher for active transport. Only trajectories with at least five data points were analyzed to calculate transport metrics. Run length was defined as the net displacement of each active (directed) segment, pause duration as the cumulative time spent in confined/diffusive phases (*R* < 0.7), and speed as the instantaneous velocity averaged over active transport phases only. As speed was computed exclusively within directed segments, it is independent of the frequency and duration of pauses.

#### Confocal imaging

Confocal images were acquired using a Laser Scanning Confocal Microscope (LSM 780) under 40X or 63X oil-immersion objectives. Optical sectioning was performed prior to image acquisition, and the images were processed using Fiji or Zen Lite software (Version 5.0).

#### Immunoprecipitation and Western blot

BSC1 cells expressing either LC3^WT^ or its mutant variants were lysed using RIPA buffer supplemented with a protease inhibitor cocktail (Promega) and a phosphatase inhibitor cocktail (GBioSciences, #786-870). Cell lysates were kept on ice for 30 min and vortexed briefly four times at 15-min intervals. The samples were then sonicated and centrifuged at 14,000 rpm for 20 min at 4 °C to remove debris. The clarified supernatant was collected, and protein concentrations were determined. Equal volumes of each sample were resolved on 12% polyacrylamide gels (1 mm thickness) and subsequently transferred to PVDF membranes (Millipore, Immobilon-FL, 0.45 µm) using a semi-dry transfer system in Towbin buffer. The membranes were blocked for 1 h at room temperature with 3% BSA and then incubated overnight at 4 °C with anti-GFP primary antibody. Following three washes with TBST (10 min each), the membranes were incubated for 1 h at room temperature with HRP-conjugated secondary antibody. After another series of three 10-min TBST washes, protein bands were visualized using the Clarity Max Western ECL Substrate (Bio-Rad, #1705062) and imaged on a Bio-Rad Gel Doc XR+ system with Image Lab software.

Following 16 h of transfection, mammalian cells were treated with HBSS (Hank’s Balanced Salt Solution) and Bafilomycin A1 (25 nM) for 8 h. The cells were then gently fixed using 0.1% PFA (paraformaldehyde) for 10 min at room temperature (27 °C), after which they were lysed with RIPA buffer for 2 h at 4 °C. Equal quantities of protein from LC3^WT^ and its variant samples were incubated with 2 μg of anti-ATG3 (CST, 3415S), anti-ATG4B (CST, 5299S), and anti-ATG7 (CST, 8558S) antibodies overnight at 4 °C to promote immunoprecipitation. To capture the immune complexes, 40 μl of Protein G Agarose beads (HiMedia, DS0818) were added to the lysates and incubated for 3 h at 4 °C. The beads were then collected by centrifugation (7000 rpm, 1 min) and subjected to several washes to eliminate non-specifically attached proteins. The immunoprecipitated proteins were eluted using Laemmli SDS loading buffer, subjected to SDS-PAGE, and transferred onto PVDF membranes (150 mA for 2.5 h). The interaction of the LC3B protein was identified using a suitable anti-LC3B antibody (Sigma, L7453) and visualized with a Bio-Rad ChemiDoc imaging system.

#### Infection and gentamycin protection assay

SPN strain R6 (serotype 2, unencapsulated) (ATCC BAA-255) was grown in THY medium, composed of Todd-Hewitt broth (THB, HiMedia, Cat No. M313) and 1.5% yeast extract (HiMedia, Cat No. RM027) at 37 ⁰C in 5% CO_2_, till OD_600_ 0.4. For the immunofluorescence assay, mammalian cells (A549) transfected with LC3^WT^ and its variants were cultured on glass coverslips, and after 16 h, were subsequently infected with Streptococcus pneumoniae (SPN) strains at a multiplicity of infection (MOI) of 30. After the infection, extracellular bacteria were removed by treating the infected cells with gentamicin (400 µg/ml) for 2 h. At specified time points after infection (6 h post-infection), the cells were rinsed with 1X PBS and processed for immunofluorescence assay.

For the gentamycin protection assay (Survival assay), completely confluent A549 monolayers underwent infection with Streptococcus pneumoniae (SPN) for 1 h at a multiplicity of infection (MOI) of 20. After the infection period, extracellular bacteria were removed by treating the infected cells with gentamicin (400 µg/ml) for 2 h. The cells were then rinsed with 1X PBS, trypsinized using 0.025% trypsin EDTA, and subsequently lysed with 0.025% Triton X-100. Intracellular bacterial survival was analyzed by preparing lysates at specific time intervals following the initial 1-h infection and the subsequent 2-h antibiotic treatment. These lysates were then plated on BHI agar, with bacterial counts represented as a percentage relative to the 0-h time point (right after antibiotic treatment). To determine the fold change in bacterial survival, the survival efficiency of the test strain was compared to that of the wild-type (WT) strain, and the resulting ratio of survival efficiencies of the test culture to that of the WT was calculated and plotted. Data were represented as mean ± SD from four independent biological replicates. Experiments were not performed under blinded conditions.

### Statistical analysis

For the quantification of microscopy experiments, the statistical significance of differences between wild-type and mutant samples was assessed using the non-parametric Mann–Whitney *U*-test. Significant differences were denoted as * for *p* ≤ 0.05, ** for *p* ≤ 0.01, and “ns” for non-significant results. Exact *P* values for all pairwise comparisons are provided in Dataset EV1, and the corresponding sample sizes (*N*) are provided in Dataset EV2.

### Use of artificial intelligence (AI)

AI tools were used to assist with grammar and language correction. All content was subsequently reviewed and verified by the authors, who take full responsibility for the final text.

## Supplementary information

Appendix

Peer Review File

Dataset EV1

Dataset EV2

Movie EV1

Movie EV2

Movie EV3

Movie EV4

Movie EV5

Movie EV6

Movie EV7

Movie EV8

Movie EV9

Movie EV11

Movie EV12

Movie EV15

Movie EV16

Movie EV10

Movie EV13

Movie EV14

Source data Fig. 1

Source data Fig. 2

Source data Fig. 3

Source data Fig. 4

Source data Fig. 5

Source data Fig. 6

Expanded View Figures

## Data Availability

The data files for LC3B crowding coarse-grained MD simulations, dimer atomistic simulations, and all extracted dimer conformation can be accessed from https://github.com/CSB-Thukral-Lab/LC3B-clustering.git. The source data of this paper are collected in the following database record: biostudies:S-SCDT-10_1038-S44319-026-00931-0.

## References

[CR1] Abraham MJ, Murtola T, Schulz R, Páll S, Smith JC, Hess B, Lindahl E (2015) GROMACS: high performance molecular simulations through multi-level parallelism from laptops to supercomputers. SoftwareX 1–2:19–25

[CR2] Banerjee C, Mehra D, Song D, Mancebo A, Park JM, Kim DH, Puchner EM (2023) ULK1 forms distinct oligomeric states and nanoscopic structures during autophagy initiation. Sci Adv 9:eadh4094 10.1126/sciadv.adh4094 PMC10541014 37774021

[CR3] Berendsen HJC, van der Spoel D, van Drunen R (1995) GROMACS: a message-passing parallel molecular dynamics implementation. Comput Phys Commun 91:43–56

[CR4] De Jong DH, Singh G, Bennett WD, Arnarez C, Wassenaar TA, Schafer LV, Periole X, Tieleman DP, Marrink SJ (2013) Improved parameters for the Martini coarse-grained protein force field. J Chem Theory Comput 9:687–697 10.1021/ct300646g 26589065

[CR5] Dooley HC, Razi M, Polson HEJ, Girardin SE, Wilson MI, Tooze SA (2014) WIPI2 links LC3 conjugation with PI3P, autophagosome formation, and pathogen clearance by recruiting Atg12-5-16L1. Mol Cell 55:238–252 10.1016/j.molcel.2014.05.021 PMC4104028 24954904

[CR6] Ercoli G, Fernandes VE, Chung WY, Wanford JJ, Thomson S, Bayliss CD, Straatman K, Crocker PR, Dennison A, Martinez-Pomares L et al (2018) Intracellular replication of Streptococcus pneumoniae inside splenic macrophages contributes to persistent bacteraemia. Nat Microbiol 3:600–610 10.1038/s41564-018-0147-1 PMC6207342 29662129

[CR7] Ershov D, Phan MS, Pylvänäinen JW, Rigaud SU, Le Blanc L, Charles-Orszag A, Conway JRW, Laine RF, Roy NH, Bonazzi D et al (2022) TrackMate 7: integrating state-of-the-art segmentation algorithms into tracking pipelines. Nat Methods 19:829–832 10.1038/s41592-022-01507-1 35654950

[CR8] Ester M, Kriegel HP, Sander J, Xu X (1996) A density-based algorithm for discovering clusters in large spatial databases with noise. In: Proc 2nd Int Conf Knowledge Discovery and Data Mining (KDD-96). AAAI Press, pp 226–231.

[CR9] Fracchiolla D, Chang C, Hurley JH, Martens S (2020) A PI3K-WIPI2 positive feedback loop allosterically activates LC3 lipidation in autophagy. J Cell Biol 219:e201912098 10.1083/jcb.201912098 PMC7337497 32437499

[CR10] Frishman D, Argos P (1995) Knowledge-based protein secondary structure assignment. Proteins 23:566–579 10.1002/prot.340230412 8749853

[CR11] Fu MM, Nirschl JJ, Holzbaur EL (2014) LC3 binding to the scaffolding protein JIP1 regulates processive dynein-driven transport of autophagosomes. Dev Cell 29:577–590 10.1016/j.devcel.2014.04.015 PMC4109720 24914561

[CR12] Galluzzi L, Baehrecke EH, Ballabio A, Boya P, Bravo-San Pedro JM, Cecconi F, Choi AM, Chu CT, Codogno P, Colombo MI et al (2017) Molecular definitions of autophagy and related processes. EMBO J 36:1811–1836 10.15252/embj.201796697 PMC5494474 28596378

[CR13] Geng J, Baba M, Nair U, Klionsky DJ (2008) Quantitative analysis of autophagy-related protein stoichiometry by fluorescence microscopy. J Cell Biol 182:129–140 10.1083/jcb.200711112 PMC2447896 18625846

[CR14] González A, Covarrubias-Pinto A, Bhaskara RM, Glogger M, Kuncha SK, Xavier A, Seemann E, Misra M, Hoffmann ME, Bräuning B et al (2023) Ubiquitination regulates ER-phagy and remodelling of endoplasmic reticulum. Nature 618:394–401 10.1038/s41586-023-06089-2 PMC10247366 37225996

[CR15] Guizetti J, Schermelleh L, Mäntler J, Maar S, Poser I, Leonhardt H, Müller-Reichert T, Gerlich DW (2011) Cortical constriction during abscission involves helices of ESCRT-III-dependent filaments. Science 331:1616–1620 10.1126/science.1201847 21310966

[CR16] Hess B, Bekker H, Berendsen HJC, Fraaije JGEM (1997) LINCS: a linear constraint solver for molecular simulations. J Comput Chem 18:1463–1472

[CR17] Huang B, Babcock H, Zhuang X (2010) Breaking the diffraction barrier: super-resolution imaging of cells. Cell 143:1047–1058 10.1016/j.cell.2010.12.002 PMC3272504 21168201

[CR18] Huang J, Rauscher S, Nawrocki G, Ran T, Feig M, de Groot BL, Grubmüller H, MacKerell AD (2016) CHARMM36m: an improved force field for folded and intrinsically disordered proteins. Nat Methods 14:71–73 10.1038/nmeth.4067 PMC5199616 27819658

[CR19] Ichimura Y, Imamura Y, Emoto K, Umeda M, Noda T, Ohsumi Y (2004) In vivo and in vitro reconstitution of Atg8 conjugation essential for autophagy. J Biol Chem 279:40584–40592 10.1074/jbc.M405860200 15277523

[CR20] Jatana N, Ascher DB, Pires DEV, Gokhale RS, Thukral L (2019) Human LC3 and GABARAP subfamily members achieve functional specificity via specific structural modulations. Autophagy 16:239–255 10.1080/15548627.2019.1606636 PMC6984608 30982432

[CR21] Kabeya Y, Mizushima N, Ueno T, Yamamoto A, Kirisako T, Noda T, Kominami E, Ohsumi Y, Yoshimori T (2000) LC3, a mammalian homologue of yeast Apg8p, is localized in autophagosome membranes after processing. EMBO J 19:5720–5728 10.1093/emboj/19.21.5720 PMC305793 11060023

[CR22] Kaizuka T, Morishita H, Hama Y, Tsukamoto S, Matsui T, Toyota Y, Kodama A, Ishihara T, Mizushima T, Mizushima N (2016) An autophagic flux probe that releases an internal control. Mol Cell 64:835–849 10.1016/j.molcel.2016.09.037 27818143

[CR23] Kamtekar S, Schiffer JM, Xiong H, Babik JM, Hecht MH (1993) Protein design by binary patterning of polar and nonpolar amino acids. Science 262:1680–1685 10.1126/science.8259512 8259512

[CR24] Kaufmann A, Wollert T (2014) Scaffolding the expansion of autophagosomes. Autophagy 10:1343–1345 10.4161/auto.28980 PMC4203560 24963637

[CR25] Kirisako T, Ichimura Y, Okada H, Kabeya Y, Mizushima N, Yoshimori T, Ohsumi M, Takao T, Noda T, Ohsumi Y (2000) The reversible modification regulates the membrane-binding state of Apg8/Aut7 essential for autophagy and the cytoplasm to vacuole targeting pathway. J Cell Biol 151:263–276 10.1083/jcb.151.2.263 PMC2192639 11038174

[CR26] Koide A, Gilbreth RN, Esaki K, Tereshko V, Koide S (2007) High-affinity single-domain binding proteins with a binary-code interface. Proc Natl Acad Sci USA 104:6632–6637 10.1073/pnas.0700149104 PMC1871837 17420456

[CR27] Lillemeier BF, Mörtelmaier MA, Forstner MB, Huppa JB, Groves JT, Davis MM (2010) TCR and Lat are expressed on separate protein islands on T cell membranes and concatenate during activation. Nat Immunol 11:90–96 10.1038/ni.1832 PMC3273422 20010844

[CR28] Mandala VS, Fu Z, MacKinnon R (2025) Molecular contacts in self-assembling clusters of membrane proteins. Proc Natl Acad Sci USA 122:e2507112122 10.1073/pnas.2507112122 PMC12232663 40549920

[CR29] Marrink SJ, Risselada HJ, Yefimov S, Tieleman DP, de Vries AH (2007) The MARTINI force field: coarse grained model for biomolecular simulations. J Phys Chem B 111:7812–7824 10.1021/jp071097f 17569554

[CR30] Mennella V, Keszthelyi B, McDonald KL, Chhun B, Kan F, Rogers GC, Huang B, Agard DA (2012) Subdiffraction-resolution fluorescence microscopy reveals a domain of the centrosome critical for pericentriolar material organization. Nat Cell Biol 14:1159–1168 10.1038/ncb2597 PMC3767400 23086239

[CR31] Michaud-Agrawal N, Denning EJ, Woolf TB, Beckstein O (2011) MDAnalysis: a toolkit for the analysis of molecular dynamics simulations. J Comput Chem 32:2319–2327 10.1002/jcc.21787 PMC3144279 21500218

[CR32] Mizushima N, Yoshimori T, Ohsumi Y (2011) The role of Atg proteins in autophagosome formation. Annu Rev Cell Dev Biol 27:107–132 10.1146/annurev-cellbio-092910-154005 21801009

[CR33] Mohan N, Sorokina EM, Vilanova Verdeny I, Sandoval Alvarez A, Lakadamyali M (2019) Detyrosinated microtubules spatially constrain lysosomes facilitating lysosome-autophagosome fusion. J Cell Biol 218:632–643 10.1083/jcb.201807124 PMC6363446 30567713

[CR34] Nakatogawa H, Ichimura Y, Ohsumi Y (2007) Atg8, a ubiquitin-like protein required for autophagosome formation, mediates membrane tethering and hemifusion. Cell 130:165–178 10.1016/j.cell.2007.05.021 17632063

[CR35] Nakatogawa H, Suzuki K, Kamada Y, Ohsumi Y (2009) Dynamics and diversity in autophagy mechanisms: lessons from yeast. Nat Rev Mol Cell Biol 10:458–467 10.1038/nrm2708 19491929

[CR36] Nascimbeni AC, Codogno P, Morel E (2017) Phosphatidylinositol-3-phosphate in the regulation of autophagy membrane dynamics. FEBS J 284:1267–1278 10.1111/febs.13987 27973739

[CR37] Nishimura T, Tooze SA (2020) Emerging roles of ATG proteins and membrane lipids in autophagosome formation. Cell Discov 6:32 10.1038/s41421-020-0161-3 PMC7248066 32509328

[CR38] Ogawa M, Matsuda R, Takada N, Tomokiyo M, Shizukuishi S, Yamaji T, Yoshikawa Y, Yoshida M, Tanida I, Koike M et al (2018) Molecular mechanisms of *Streptococcus pneumoniae*-targeted autophagy via pneumolysin, Golgi-resident Rab41, and Nedd4-1-mediated K63-linked ubiquitination. Cell Microbiol 20:e12846 10.1111/cmi.12846 29582580

[CR39] Owen DM, Rentero C, Rossy J, Magenau A, Williamson D, Rodriguez M, Gaus K (2010) PALM imaging and cluster analysis of protein heterogeneity at the cell surface. J Biophotonics 3:446–454 10.1002/jbio.200900089 20148419

[CR40] Pankiv S, Alemu EA, Brech A, Bruun JA, Lamark T, Øvervatn A, Bjørkøy G, Johansen T et al (2010) FYCO1 is a Rab7 effector that binds to LC3 and PI3P to mediate microtubule plus end-directed vesicle transport. J Cell Biol 188:253–269 10.1083/jcb.200907015 PMC2812517 20100911

[CR41] Parrinello M, Rahman A (1981) Polymorphic transitions in single crystals: a new molecular dynamics method. J Appl Phys 52:7182–7190

[CR42] Parton R (2004) Lipid rafts and plasma membrane microorganization: insights from Ras. Trends Cell Biol 14:141–147 10.1016/j.tcb.2004.02.001 15003623

[CR43] Periole X, Cavalli M, Marrink SJ, Ceruso MA (2009) Combining an elastic network with a coarse-grained molecular force field: structure, dynamics, and intermolecular recognition. J Chem Theory Comput 5:2531–2543 10.1021/ct9002114 26616630

[CR44] Proikas-Cezanne T, Takacs Z, Dönnes P, Kohlbacher O (2015) WIPI proteins: essential PtdIns3P effectors at the nascent autophagosome. J Cell Sci 128:207–217 10.1242/jcs.146258 25568150

[CR45] Puri C, Gratian MJ, Rubinsztein DC (2023) Mammalian autophagosomes form from finger-like phagophores. Dev Cell 58:2746–2760.e5 10.1016/j.devcel.2023.08.016 37683632

[CR46] Ragusa MJ, Stanley RE, Hurley JH (2012) Architecture of the Atg17 complex as a scaffold for autophagosome biogenesis. Cell 151:1501–1512 10.1016/j.cell.2012.11.028 PMC3806636 23219485

[CR47] Rivas G, Minton AP (2016) Macromolecular crowding in vitro, in vivo, and in between. Trends Biochem Sci 41:970–981 10.1016/j.tibs.2016.08.013 PMC5804487 27669651

[CR48] Rogov VV, Nezis IP, Tsapras P, Zhang H, Dagdas Y, Noda NN, Nakatogawa H, Wirth M, Mouilleron S, McEwan DG et al (2023) Atg8 family proteins, LIR/AIM motifs and other interaction modes. Autophagy Rep 2:2188523 10.1080/27694127.2023.2188523 PMC7615515 38214012

[CR49] Rust MJ, Bates M, Zhuang X (2006) Sub-diffraction-limit imaging by stochastic optical reconstruction microscopy (STORM). Nat Methods 3:793–796 10.1038/nmeth929 PMC2700296 16896339

[CR50] Santra S, Nayak I, Paladhi A, Das D, Banerjee A (2024) Estimates of differential toxin expression governing heterogeneous intracellular lifespans of *Streptococcus pneumoniae*. J Cell Sci 137:jcs260891 10.1242/jcs.260891 38411297

[CR51] Sawa-Makarska J, Baumann V, Coudevylle N, von Bülow S, Nogellova V, Abert C, Schuschnig M, Graef M, Hummer G, Martens S (2020) Reconstitution of autophagosome nucleation defines Atg9 vesicles as seeds for membrane formation. Science 369:eaaz7714 10.1126/science.aaz7714 PMC7610778 32883836

[CR52] Sharma P, Varma R, Sarasij RC, Ira, Gousset K, Krishnamoorthy G, Rao M, Mayor S (2004) Nanoscale organization of multiple GPI-anchored proteins in living cell membranes. Cell 116:577–589 10.1016/s0092-8674(04)00167-9 14980224

[CR53] Shewell LK, Harvey RJ, Badylak SF, Mitchell TJ, Andrew PW, Standish AJ, Weiser JN (2014) The cholesterol-dependent cytolysins pneumolysin and streptolysin O require binding to red blood cell glycans for hemolytic activity. Proc Natl Acad Sci USA 111:E5312–E5320 10.1073/pnas.1412703111 PMC4267402 25422425

[CR54] Smith P, Lorenz CD (2021) LiPyphilic: a Python toolkit for the analysis of lipid membrane simulations. J Chem Theory Comput 17:5907–5919 10.1021/acs.jctc.1c00447 34450002

[CR55] Sou YS, Tanida I, Komatsu M, Ueno T, Kominami E (2006) Phosphatidylserine in addition to phosphatidylethanolamine is an in vitro target of the mammalian Atg8 modifiers, LC3, GABARAP, and GATE-16. J Biol Chem 281:3017–3024 10.1074/jbc.M505888200 16303767

[CR56] Surve MV, Bhutda S, Datey A, Anil A, Rawat S, Pushpakaran A, Singh D, Kim KS, Chakravortty D, Banerjee A (2018) Heterogeneity in pneumolysin expression governs the fate of *Streptococcus pneumoniae* during blood-brain barrier trafficking. PLoS Pathog 14:e1007168 10.1371/journal.ppat.1007168 PMC6062133 30011336

[CR57] Suzuki K, Kirisako T, Kamada Y, Mizushima N, Noda T, Ohsumi Y (2001) The pre-autophagosomal structure organized by concerted functions of APG genes is essential for autophagosome formation. EMBO J 20:5971–5981 10.1093/emboj/20.21.5971 PMC125692 11689437

[CR58] Tanida I, Ueno T, Kominami E (2004) Human light chain 3/MAP1LC3B is cleaved at its carboxyl-terminal Met121 to expose Gly120 for lipidation and targeting to autophagosomal membranes. J Biol Chem 279:47704–47710 10.1074/jbc.M407016200 15355958

[CR59] Tanida I, Waguri S (2010) Measurement of autophagy in cells and tissues. Methods Mol Biol 648:193–214 10.1007/978-1-60761-756-3_13 20700714

[CR60] Thukral L, Sengupta D, Ramkumar A, Murthy D, Agrawal N, Gokhale RS (2015) The molecular mechanism underlying recruitment and insertion of lipid-anchored LC3 protein into membranes. Biophys J 109:2067–2078 10.1016/j.bpj.2015.09.022 PMC4656858 26588566

[CR61] Thurston TL, Wandel MP, von Mühlen N, Foeglein A, Randow F (2012) Galectin 8 targets damaged vesicles for autophagy to defend cells against bacterial invasion. Nature 482:414–418 10.1038/nature10744 PMC3343631 22246324

[CR62] Tooze SA, Yoshimori T (2010) The origin of the autophagosomal membrane. Nat Cell Biol 12:831–835 10.1038/ncb0910-831 20811355

[CR63] Tripathi S, Huda S, Kar J, Chandra D, Jayandharan GR, Mohan N (2025) Suppressing microtubule detyrosination augments adeno-associated virus 2 endosomal escape and gene delivery. J Cell Sci 138:jcs264190 10.1242/jcs.264190 PMC12752493 41140178

[CR64] Veeraraghavan R, Gourdie RG (2016) Stochastic optical reconstruction microscopy-based relative localization analysis (STORM-RLA) for quantitative nanoscale assessment of spatial protein organization. Mol Biol Cell 27:3583–3590 10.1091/mbc.E16-02-0125 PMC5221590 27307586

[CR65] Verdeny-Vilanova I, Wehnekamp F, Mohan N, Sandoval Álvarez Á, Borbely JS, Otterstrom JJ, Lamb DC, Lakadamyali M (2017) 3D motion of vesicles along microtubules helps them to circumvent obstacles in cells. J Cell Sci 130:1904–1916 10.1242/jcs.201178 PMC5482975 28420672

[CR66] Wagner JR, Sørensen J, Hensley N, Wong C, Zhu C, Perison T, Amaro RE (2017) POVME 3.0: software for mapping binding pocket flexibility. J Chem Theory Comput 13:4584–4592 10.1021/acs.jctc.7b00500 PMC5751414 28800393

[CR67] Wassenaar TA, Ingólfsson HI, Böckmann RA, Tieleman DP, Marrink SJ (2015) Computational lipidomics with insane: a versatile tool for generating custom membranes for molecular simulations. J Chem Theory Comput 11:2144–2155 10.1021/acs.jctc.5b00209 26574417

[CR68] Wassenaar TA, Pluhackova K, Böckmann RA, Marrink SJ, Tieleman DP (2014) Going backward: a flexible geometric approach to reverse transformation from coarse grained to atomistic models. J Chem Theory Comput 10:676–690 10.1021/ct400617g 26580045

[CR69] Weidberg H, Shpilka T, Shvets E, Abada A, Shimron F, Elazar Z (2011) LC3 and GATE-16 N termini mediate membrane fusion processes required for autophagosome biogenesis. Dev Cell 20:444–454 10.1016/j.devcel.2011.02.006 21497758

[CR70] Weidberg H, Shvets E, Shpilka T, Shimron F, Shinder V, Elazar Z (2010) LC3 and GATE-16/GABARAP subfamilies are both essential yet act differently in autophagosome biogenesis. EMBO J 29:1792–1802 10.1038/emboj.2010.74 PMC2885923 20418806

[CR71] Weiser JN, Ferreira DM, Paton JC (2018) Streptococcus pneumoniae: transmission, colonization and invasion. Nat Rev Microbiol 16:355–367 10.1038/s41579-018-0001-8 PMC5949087 29599457

[CR72] Wirth M, Zhang W, Razi M, Nyoni L, Joshi D, O’Reilly N, Johansen T, Tooze SA, Mouilleron S (2019) Molecular determinants regulating selective binding of autophagy adapters and receptors to ATG8 proteins. Nat Commun 10:2055 10.1038/s41467-019-10059-6 PMC6499816 31053714

[CR73] Wurm CA, Neumann D, Lauterbach MA, Harke B, Egner A, Hell SW, Jakobs S (2011) Nanoscale distribution of mitochondrial import receptor Tom20 is adjusted to cellular conditions and exhibits an inner-cellular gradient. Proc Natl Acad Sci USA 108:13546–13551 10.1073/pnas.1107553108 PMC3158204 21799113

[CR74] Xie Z, Nair U, Geng J, Szefler MB, Rothman ED, Klionsky DJ (2009) Indirect estimation of the area density of Atg8 on the phagophore. Autophagy 5:217–220 10.4161/auto.5.2.7201 PMC2941343 19088501

[CR75] Xie Z, Nair U, Klionsky DJ (2008) Atg8 controls phagophore expansion during autophagosome formation. Mol Biol Cell 19:3290–3298 10.1091/mbc.E07-12-1292 PMC2488302 18508918

[CR76] Xu G, Wang W, Groves JT, Hecht MH (2001) Self-assembled monolayers from a designed combinatorial library of de novo β-sheet proteins. Proc Natl Acad Sci USA 98:3652–3657 10.1073/pnas.071400098 PMC31106 11274383

[CR77] Yang Z, Klionsky DJ (2010) Eaten alive: a history of macroautophagy. Nat Cell Biol 12:814–822 10.1038/ncb0910-814 PMC3616322 20811353

[CR78] Ye Y, Tyndall ER, Bui V, Bewley MC, Wang G, Hong X, Shen Y, Flanagan JM, Wang HG, Tian F (2023) Multifaceted membrane interactions of human Atg3 promote LC3-phosphatidylethanolamine conjugation during autophagy. Nat Commun 14:5503 10.1038/s41467-023-41243-4 PMC10485044 37679347

[CR79] Yim WWY, Mizushima N (2020) Lysosome biology in autophagy. Cell Discov 6:6. 10.1038/s41421-020-0141-7 PMC7010707 32047650

[CR80] Yu L, Chen Y, Tooze SA (2017) Autophagy pathway: cellular and molecular mechanisms. Autophagy 14:207–215 10.1080/15548627.2017.1378838 PMC5902171 28933638

[CR81] Zhang W, Nishimura T, Gahlot D, Saito C, Davis C, Jefferies HB, Schreiber A, Thukral L, Tooze SA (2023) Autophagosome membrane expansion is mediated by the N-terminus and cis-membrane association of human ATG8s. eLife 12:e89185 10.7554/eLife.89185 PMC10289813 37288820

[CR82] Zhou Y, Hancock JF (2023) RAS nanoclusters are cell surface transducers that convert extracellular stimuli to intracellular signalling. FEBS Lett 597:892–908 10.1002/1873-3468.14569 PMC10919257 36595205

[CR83] Zhou Y, Prakash P, Liang H, Cho KJ, Gorfe AA, Hancock JF (2017) Lipid-sorting specificity encoded in K-Ras membrane anchor regulates signal output. Cell 168:239–251.e16 10.1016/j.cell.2016.11.059 PMC5653213 28041850

