# Supplementary material for: LC3 forms functional nanoclusters on autophagosomes
Source: EMBO Rep. 2026 Sep 25;27(21):6139–65. doi: 10.1038/s44319-026-00931-0 (PMC13743770; doi:10.1038/s44319-026-00931-0)
Supplement: Supplementary file 1 [file 44319_2026_931_MOESM1_ESM.pdf]

Appendix to

# LC3 forms functional nanoclusters on autophagosome

Fatihi & Khushalani, et al.

*Corresponding author. Email: nitinm@iitk.ac.in; lipi.thukral@igib.res.in

## Table of Contents


**Appendix Figure S1** Membrane-interacting module (MTM) in the clustered population of LC3 ........ 3
**Appendix Figure S2** Progression of LC3B cluster formation over the 10 µs coarse-grained trajectory ........ 4
**Appendix Figure S3** LC3B clustering is curvature-independent locally but membrane shows global undulations ........ 6
**Appendix Figure S4** PI lipids bind LC3B and accumulate in the vicinity of LC3 clusters ........ 7
**Appendix Figure S5** TIRF imaging of autophagosomes marked by LC3 immunostaining ........ 9
**Appendix Figure S6** Cell-wise distribution of LC3B nanocluster number and size ........ 10
**Appendix Figure S7** Correlation between autophagosome size and LC3B cluster size ........ 11
**Appendix Figure S8** LC3 is progressively recruited as autophagosomes grow ........ 12
**Appendix Figure S9** Hetero-clustering of LC3B and GABARAP in mixed simulations ........ 13
**Appendix Figure S10** Independent PX probe confirms PI3P–LC3 colocalization during autophagy initiation. ........ 14
**Appendix Figure S11** Workflow for identifying clustering interfaces ........ 15
**Appendix Figure S12** Time evolution of the top binding pose interactions forming the interface ........ 16
**Appendix Figure S13** LC3 protein–protein interaction map derived from experimental complexes ........ 17
**Appendix Figure S14** Evolutionary conservation of the rear binding pocket ........ 18
**Appendix Figure S15** Relative residue accessibility at the Loop 6–α4 interface ........ 19
**Appendix Figure S16** Canonical binding pocket is non-overlapping with the LC3B clustering interface ........ 20
**Appendix Figure S17** Crystal packing reveals a recurrent LC3 self-association interface ........ 21
**Appendix Figure S18** In silico characterisation of the Loop 3 and C-terminal LC3B clustering mutants ........ 22
**Appendix Figure S19** Lipidation and endogenous ATG8 levels are preserved across GFP-LC3B variants ........ 23
**Appendix Figure S20** Diminished association of LC3B Loop6Mutant2 with PI3P-positive vesicles ........ 24
**Appendix Figure S21** Autophagosome motility is impaired in LC3B clustering mutants ........ 25
**Appendix Figure S22** Interaction of FYCO1 with different GFP-LC3 variants ........ 26
**Appendix Figure S23** Immunoblot demonstrating autophagy flux as p62 accumulation as a readout ........ 27
**Appendix Figure S24** Mutant LC3 shows unaltered interaction with the autophagic machinery ........ 28

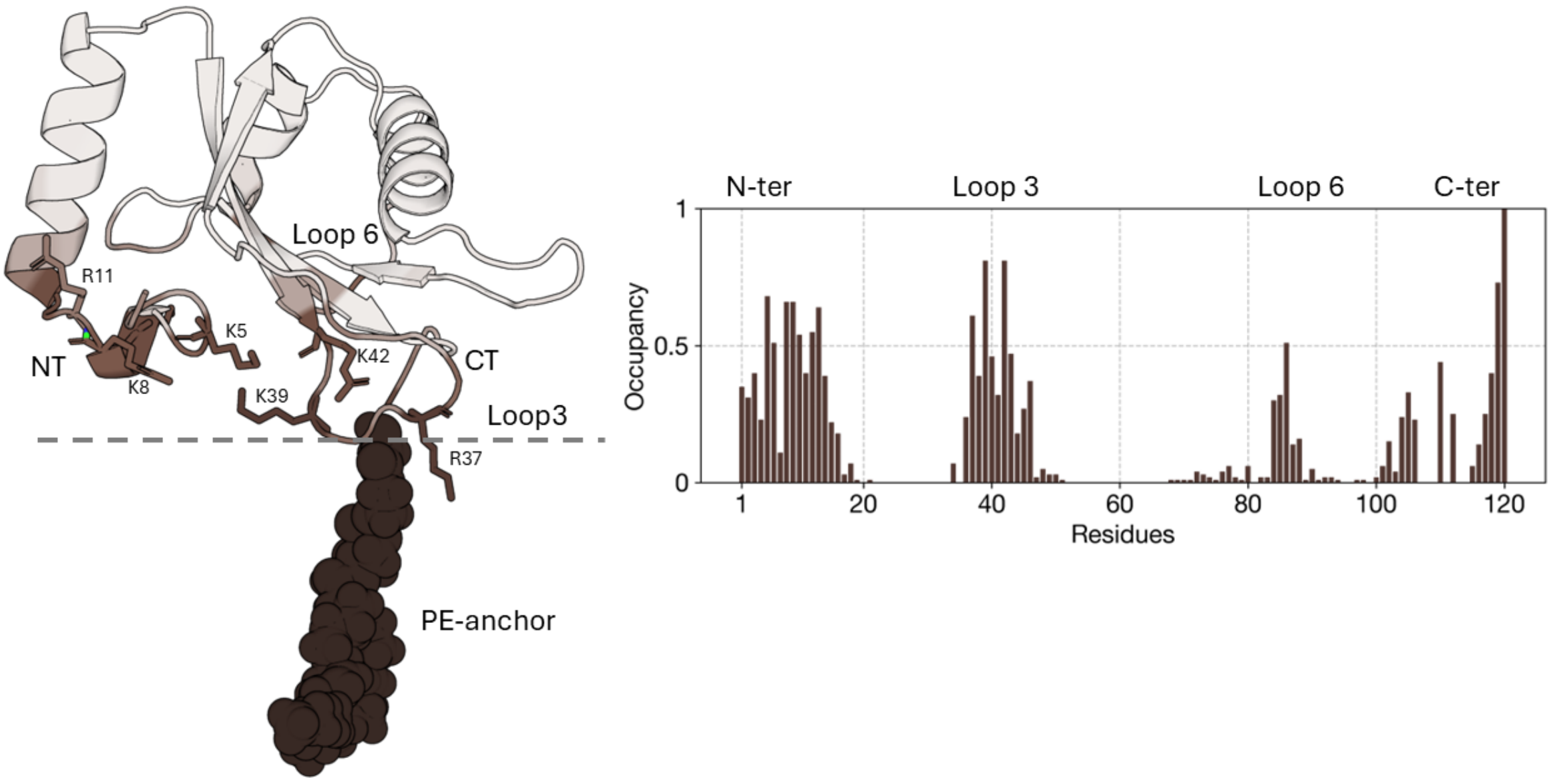


**Appendix Figure S1. Membrane-interacting module (MTM) in the clustered population of LC3.** Residues interacting with the membrane are mapped onto a representative structure, with color intensity reflecting interaction occupancy during the final 5 µs of the simulation trajectory (interaction cutoff distance was 5 Å). The accompanying bar plot shows the average occupancy values for each residue across all 90 chains.

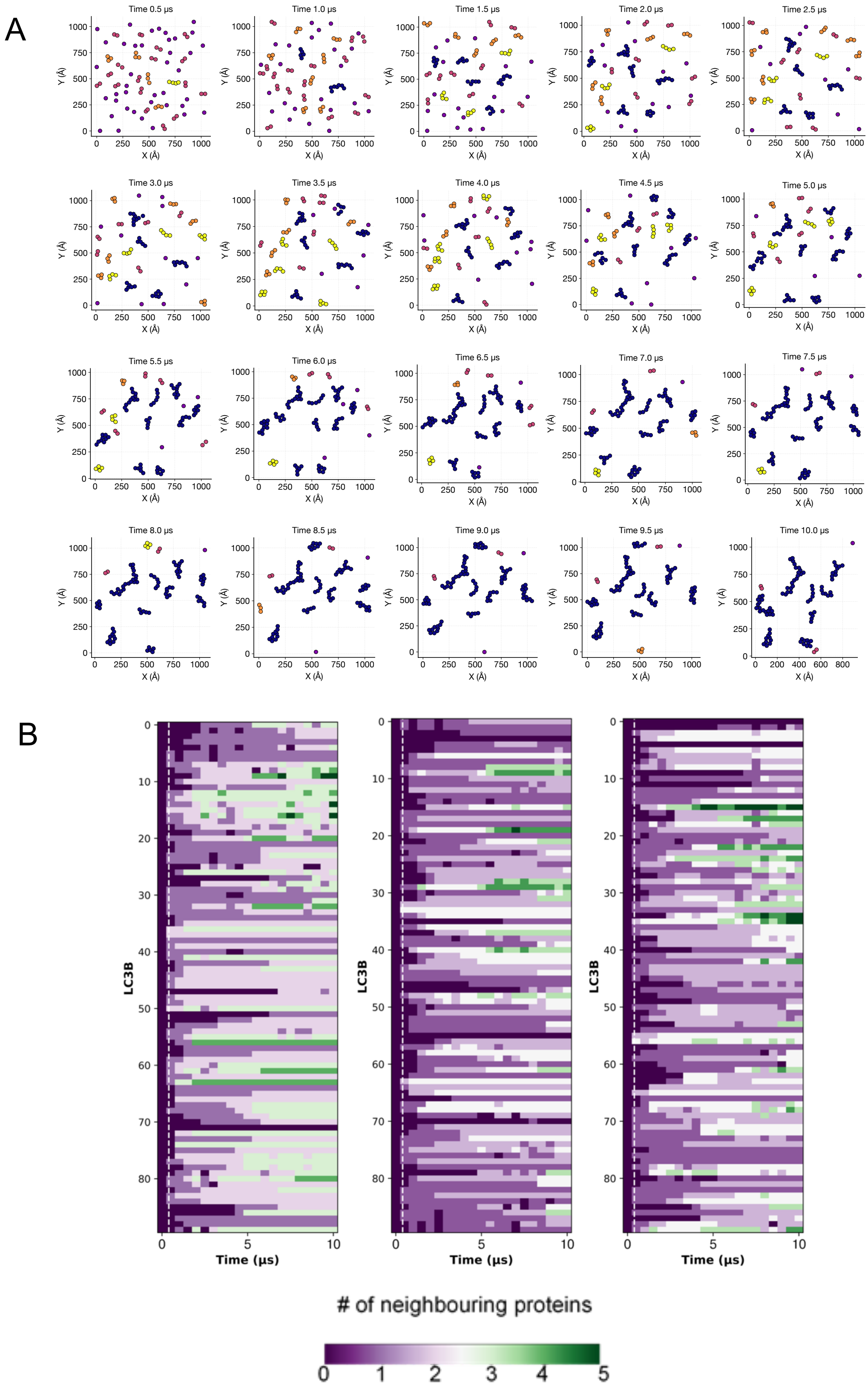


**Appendix Figure S2. Progression of LC3B cluster formation over the 10 μs coarse-grained trajectory.** (A) Positions of LC3 molecules plotted relative to the dimensions of the simulation box at 500 ns intervals to track the progression of cluster formation. Circle colours represent cluster types: purple

(monomer), pink (dimer), orange (trimer), yellow (tetramer) and dark blue (oligomer). The cluster distribution is displayed for all three simulation replicates. (B) Heatmap showing the number of neighbouring proteins in LC3B clusters. Each of the three boxes represents a separate simulation, with each row within a box corresponding to an individual protein over the simulation time. The colour scale indicates the number of neighbouring proteins, defined by a 5 nm cutoff distance between the centres of mass of two proteins. Neighbour counts range from 0 (purple) to 6 (green).

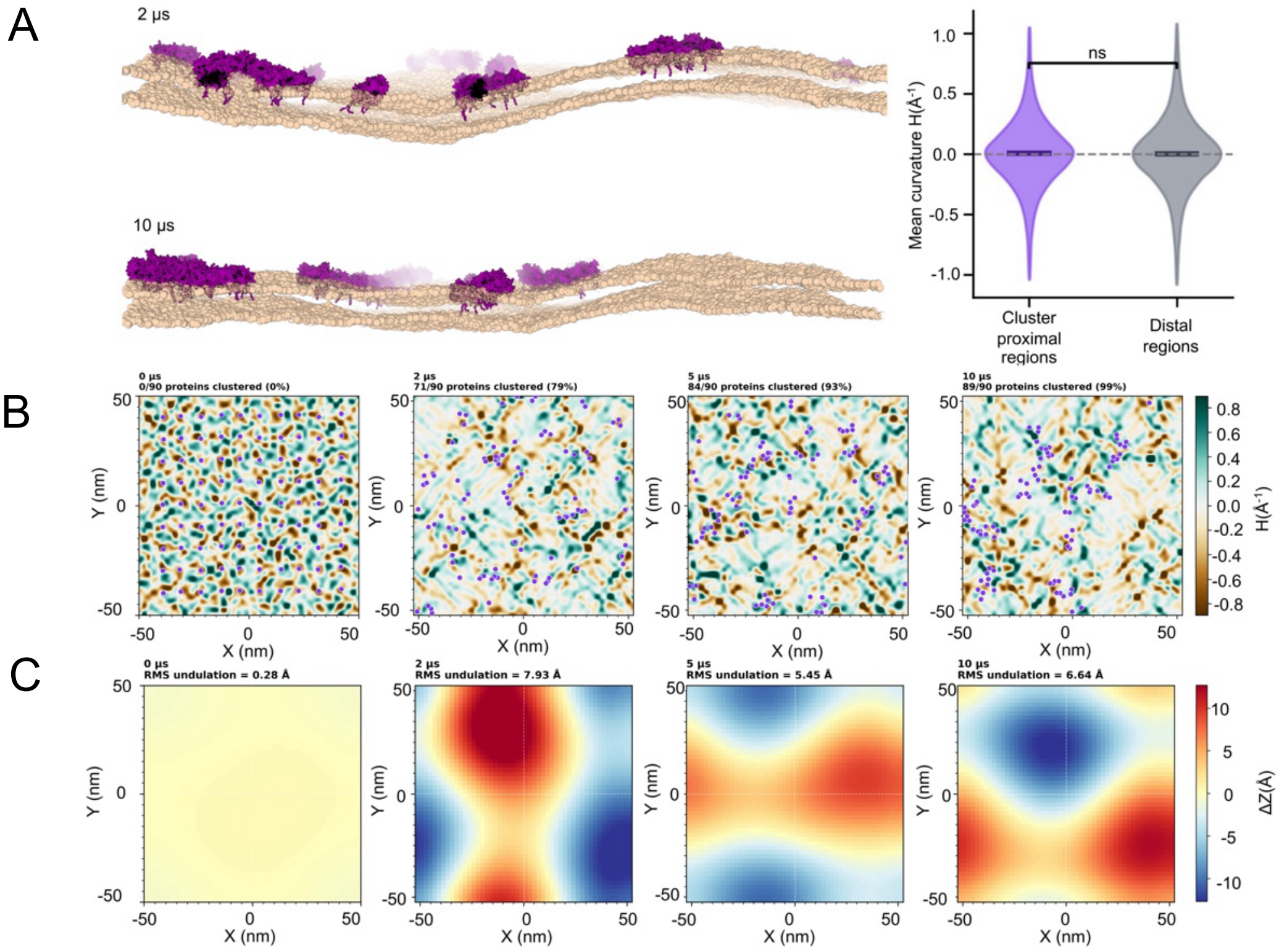


**Appendix Figure S3: LC3B clustering is curvature-independent locally but membrane shows global undulations.** (A) Representative coarse-grained snapshots showing LC3B (magenta) on the membrane illustrating subtle membrane curvature. Top right: Distribution of local mean curvature (H) in cluster-proximal and distal regions shows no significant difference (cluster: 0.00185 ± 0.32 $Å^{-1}$; non-cluster: −0.00329 ± 0.32 $Å^{-1}$; Mann–Whitney p = 0.3065, n.s.), indicating absence of curvature enrichment at cluster sites. (B) Spatial maps of mean curvature (*H*) at different time points with LC3B centers of mass overlaid (purple). Despite increasing clustering (0–99%) curvature remains spatially heterogeneous without systematic localization to clusters. (C) Corresponding membrane height deviation (Δ*z*) maps at the same time points. As clustering proceeds, the membrane develops large-scale smooth undulations spanning tens of nanometres. RMS undulation amplitude increases from ~0.28 Å at 0 µs to ~6.64 Å at 10 µs, stabilising at a mean of 4.47 ± 2.15 Å across the simulation. These undulations reflect collective deformation of the membrane by the growing LC3 protein assemblies rather than local deformation at individual cluster sites.

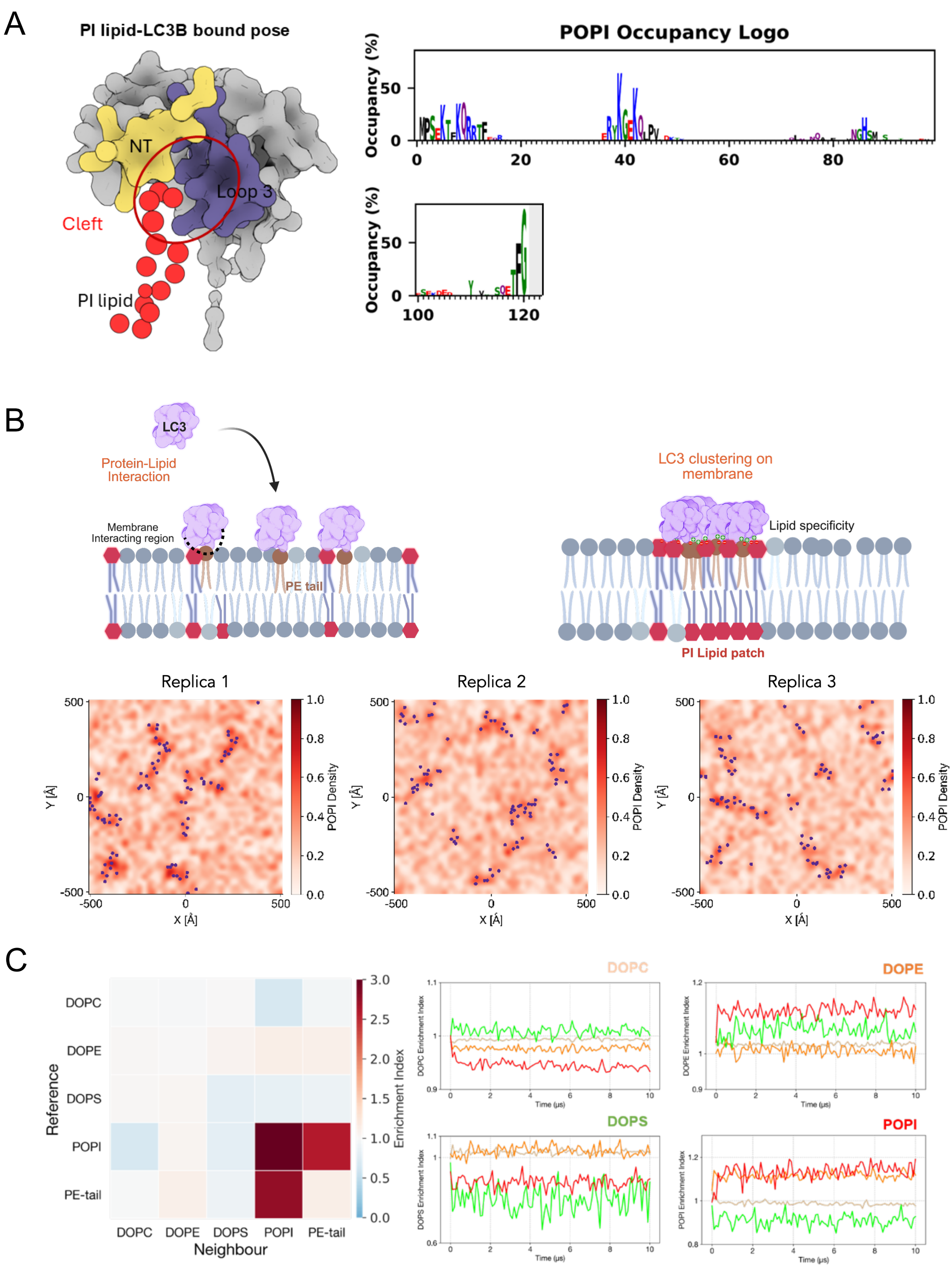


**Appendix Figure S4. PI lipids bind LC3B and accumulate in the vicinity of LC3 clusters.** (A) Representative bound pose of a phosphatidylinositol (PI) lipid species interacting with LC3B. The top-ranked pose, identified through back-mapping from coarse-grained simulations, reveals binding within a cleft formed between Loop 3 and the N-terminal region of LC3B. The accompanying logo plot depicts the amino acid composition of the binding site, which is predominantly enriched in positively charged residues. (B) Preferential accumulation of PI lipids in the vicinity of LC3 clusters. Schematic illustrating PI-lipid enrichment around LC3 clusters, together with the probability distribution of lipid density calculated across the simulation XY plane. Dots represent LC3 proteins; a substantial overlay is

observed with PI lipids. (C) Heatmap showing lipid presence and depletion around LC3B, with the enrichment and depletion index for each lipid species calculated from the number of lipids within 12 Å of LC3B in each frame. Index values above 1 indicate lipid enrichment, while values below 1 indicate lipid depletion. The heatmap emphasises the dynamic clustering and distribution of POPI lipid species near LC3B. The accompanying line plot depicts the self-enrichment index of POPI lipids over time, providing a temporal perspective on lipid dynamics.

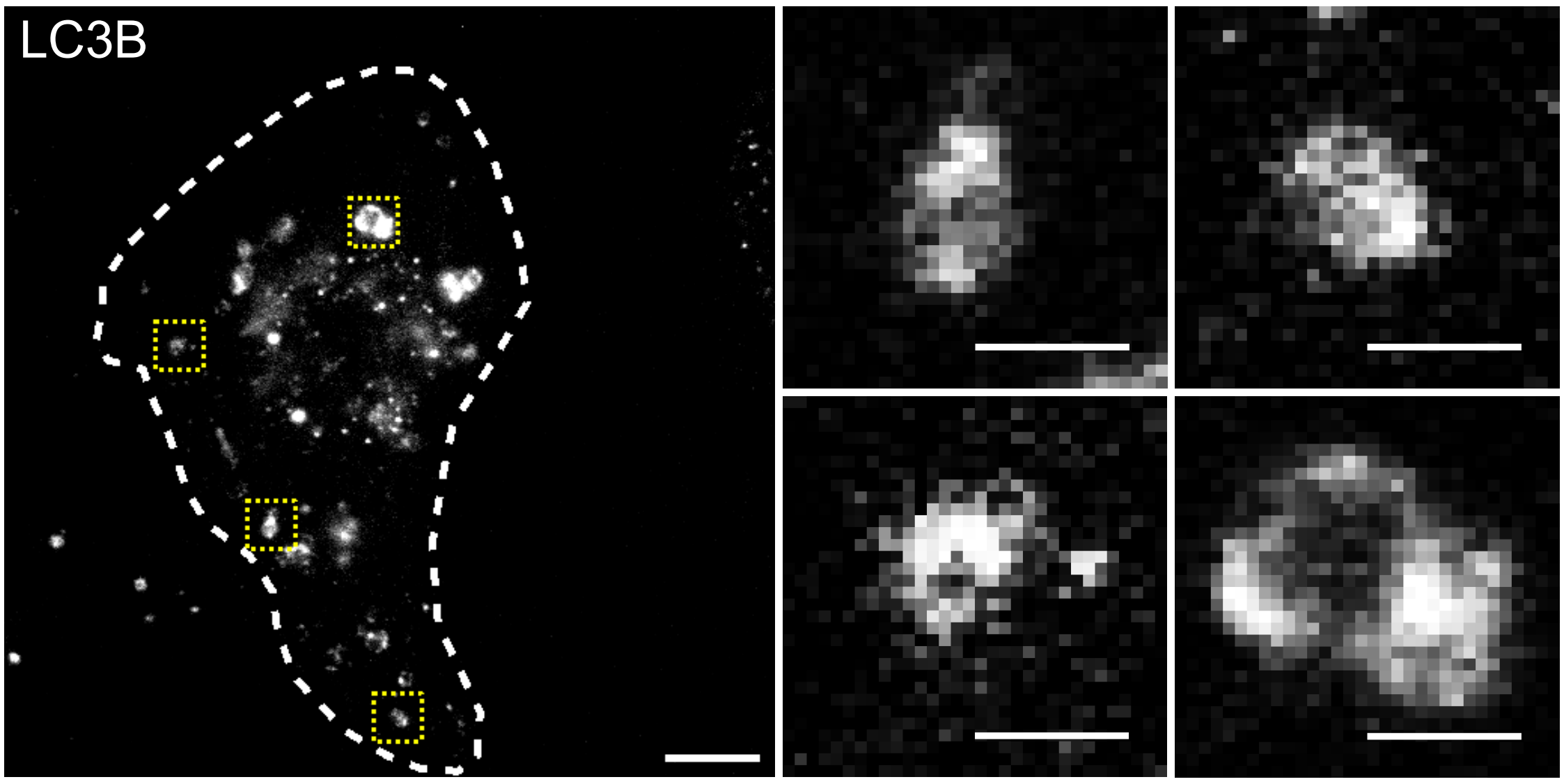


**Appendix Figure S5. TIRF imaging of autophagosome marked by LC3 immunostaining**: Conventional TIRF microscopy image of autophagosomes reveals LC3 localised to autophagosome recapitulate autophagosome structure, traditionally referred as autophagosome puncta. The diffraction limited TIRF imaging does not resolve the individual LC3B proteins localised on autophagosome. Scale bar 10 µm (image), 2 µm (zoom).

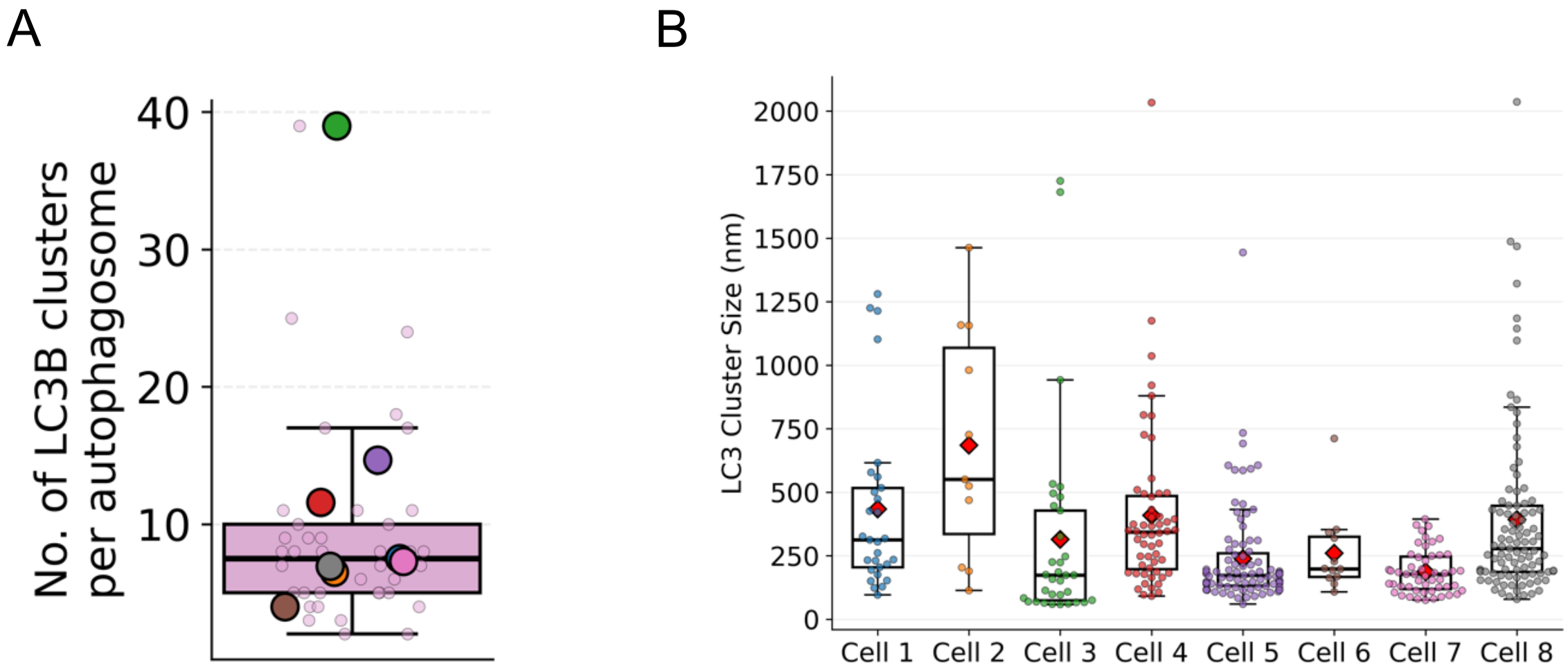


**Appendix Figure S6. Cell-wise distribution of LC3B nanocluster number and size.** (A) Average number of LC3B nanoclusters per autophagosome across N = 8 cells. Data replotted from Figure 2D as Box plots. Large circles show individual cell means, overlaid on single-autophagosome data. Box plots represent the median, interquartile range, and SD across all cells. (B) Data replotted from Figure 2F as Box plots showing size distribution of LC3B cluster size across 8 individual cells. Red diamonds marking the cell mean, highlighting single-vesicle size variation within individual cells.

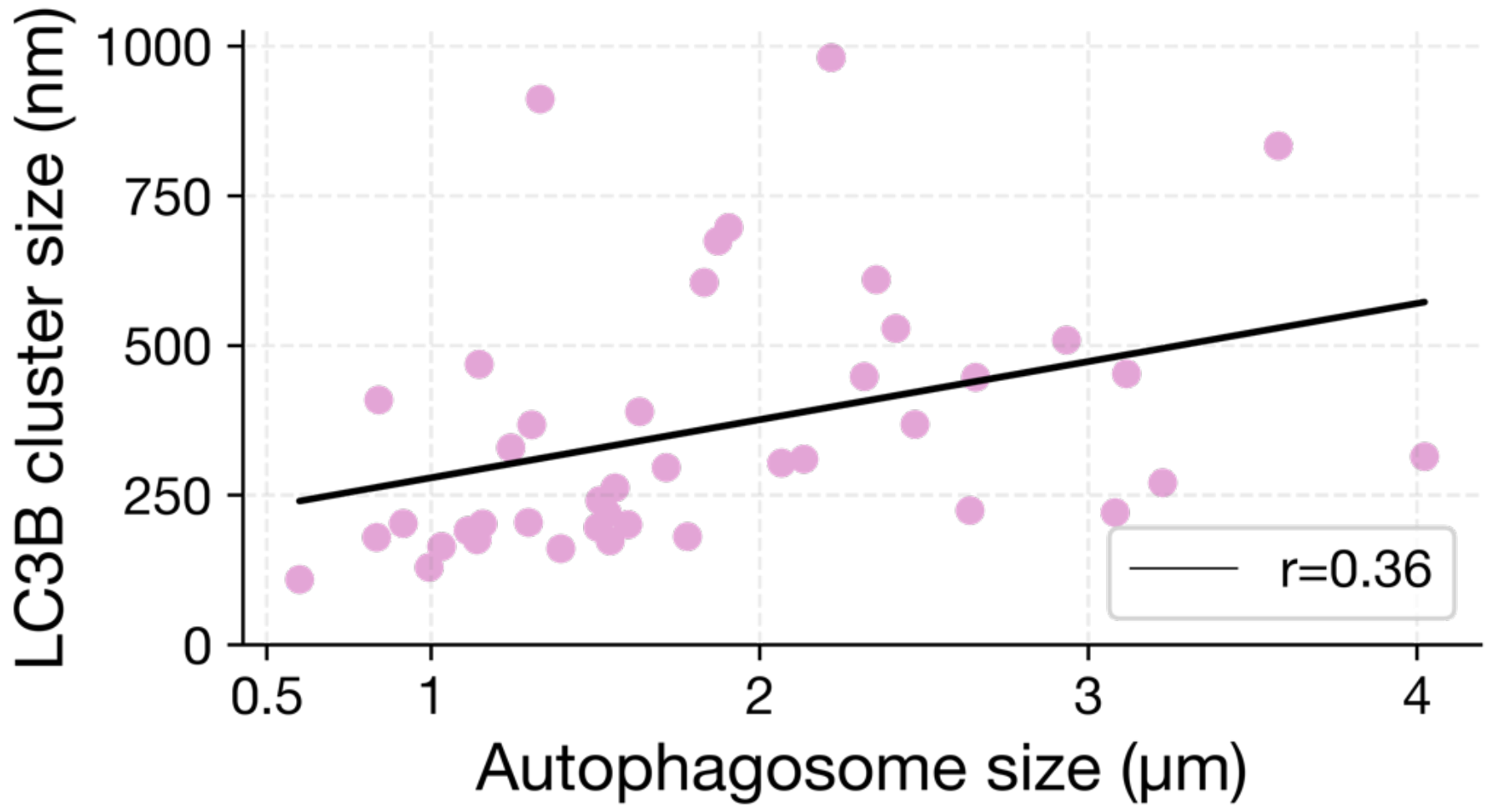


**Appendix Figure S7. Correlation analysis between autophagosome size and LC3B cluster size.** The average size of LC3 clusters does not proportionally vary with autophagosome size variability. The data represented here is from 8 cells across two independent experiments.

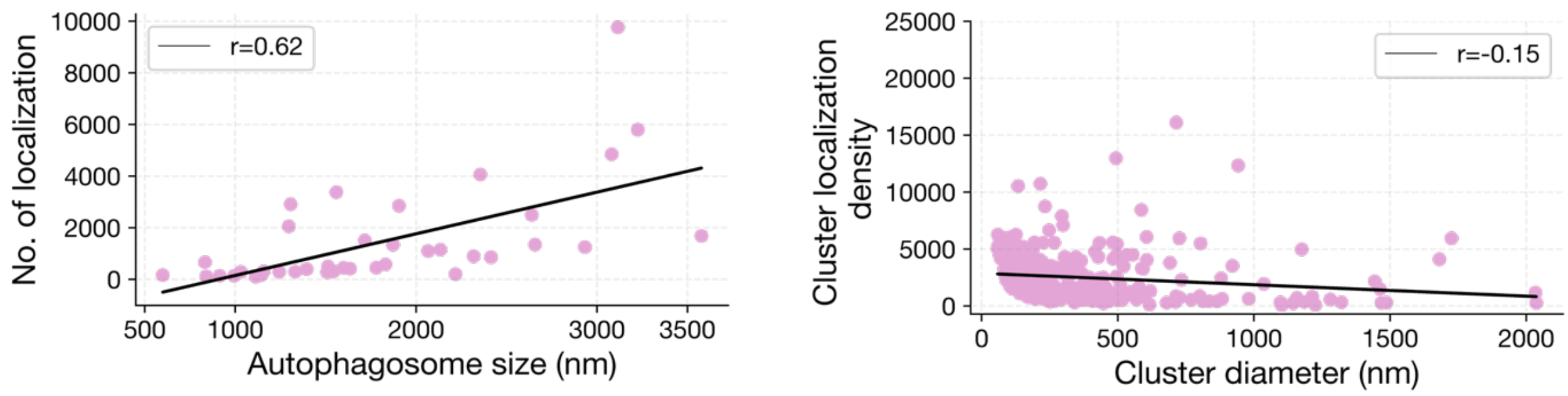


**Appendix Figure S8. LC3 is progressively recruited as autophagosomes grow.** (Left) Total number of LC3 localizations per autophagosome shows a positive correlation with autophagosome size, consistent with progressive LC3 recruitment during autophagosome growth. (Right) LC3 nanocluster localization density shows no significant correlation with cluster size. The data represented here is from 8 cells across two independent experiments.

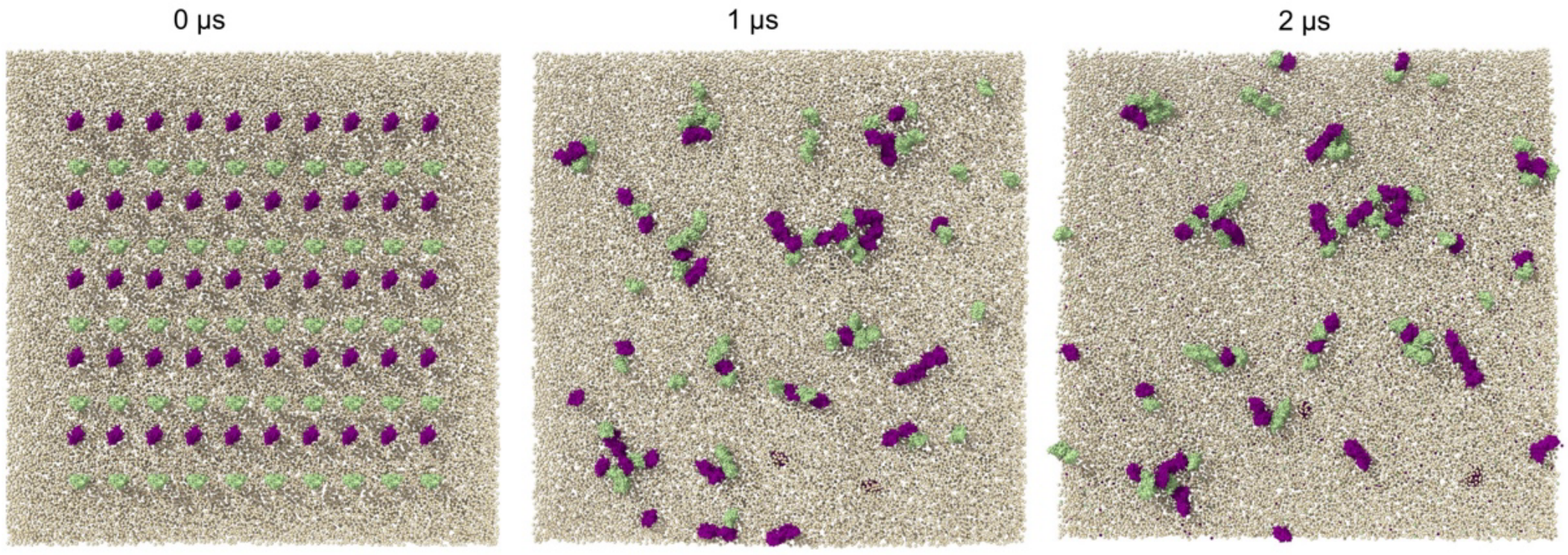


**Appendix Figure S9. Hetero clustering of LC3B and GABARAP in mixed simulations.** Representative snapshots from coarse-grained molecular dynamics simulations of a mixed system containing 50 LC3B (purple) and 50 GABARAP (green) molecules at 1.0 and 1.5 μs. The proteins exhibit a tendency to form mixed clusters, with LC3B and GABARAP frequently co-localizing within shared assemblies, suggesting that heterotypic clustering can occur under these simulation conditions.

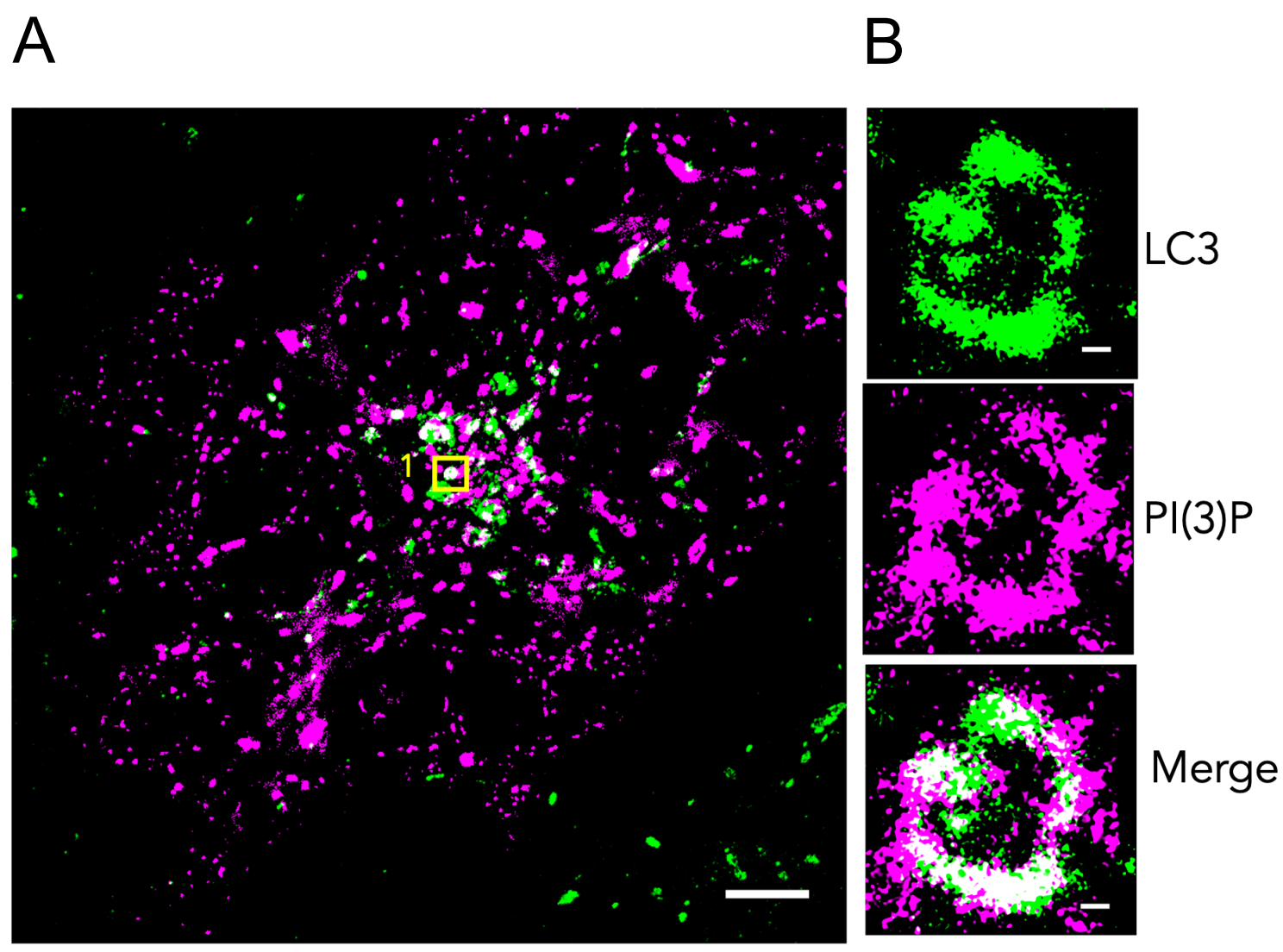


**Appendix Figure S10. Independent PX probe confirms PI3P–LC3 colocalization during autophagy initiation.** (A) Representative confocal imaging of starved BS-C-1 cells overexpressing the p40phox-PX domain tagged with GFP, immunostained for endogenous LC3B (green), and for GFP (magenta). (B) Regions of interest (ROI-yellow) from sequential 2-colour 2D STORM imaging of the cell marked in (A), showing colocalization with LC3B. Scale bars:(A) 10 µm; (B) 200 nm

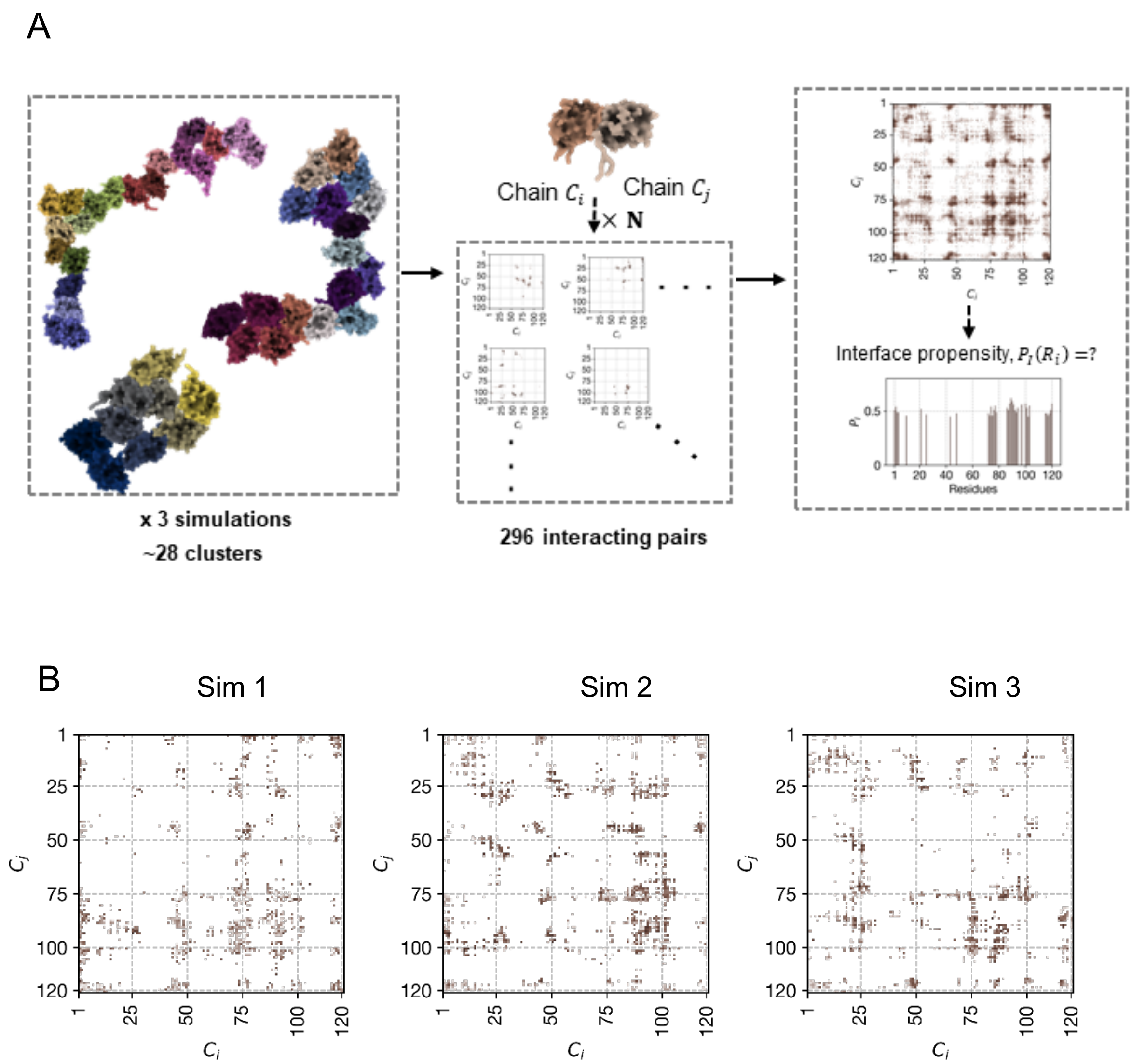


**Appendix Figure S11: Appendix Figure S11. Workflow for identifying LC3B clustering interfaces and mapping residue interaction propensities.** (A) Schematic overview of the analysis pipeline used to identify protein–protein interfaces from coarse-grained molecular dynamics simulations. A total of 28 LC3B homo-oligomeric clusters, comprising 296 unique dimer interfaces, were extracted and analyzed. For each interface, residue–residue contact maps were generated to quantify the propensity of individual residues to participate in clustering interactions. (B) Contact matrices for all residue pairs across three simulations showing contacts with an occupancy greater than 0.3 across all interfaces observed in each simulation. The color scale represents contact occupancy, ranging from 0 (no contact) to 1 (persistent contact), highlighting the interaction hotspots that define the LC3B clustering interface.

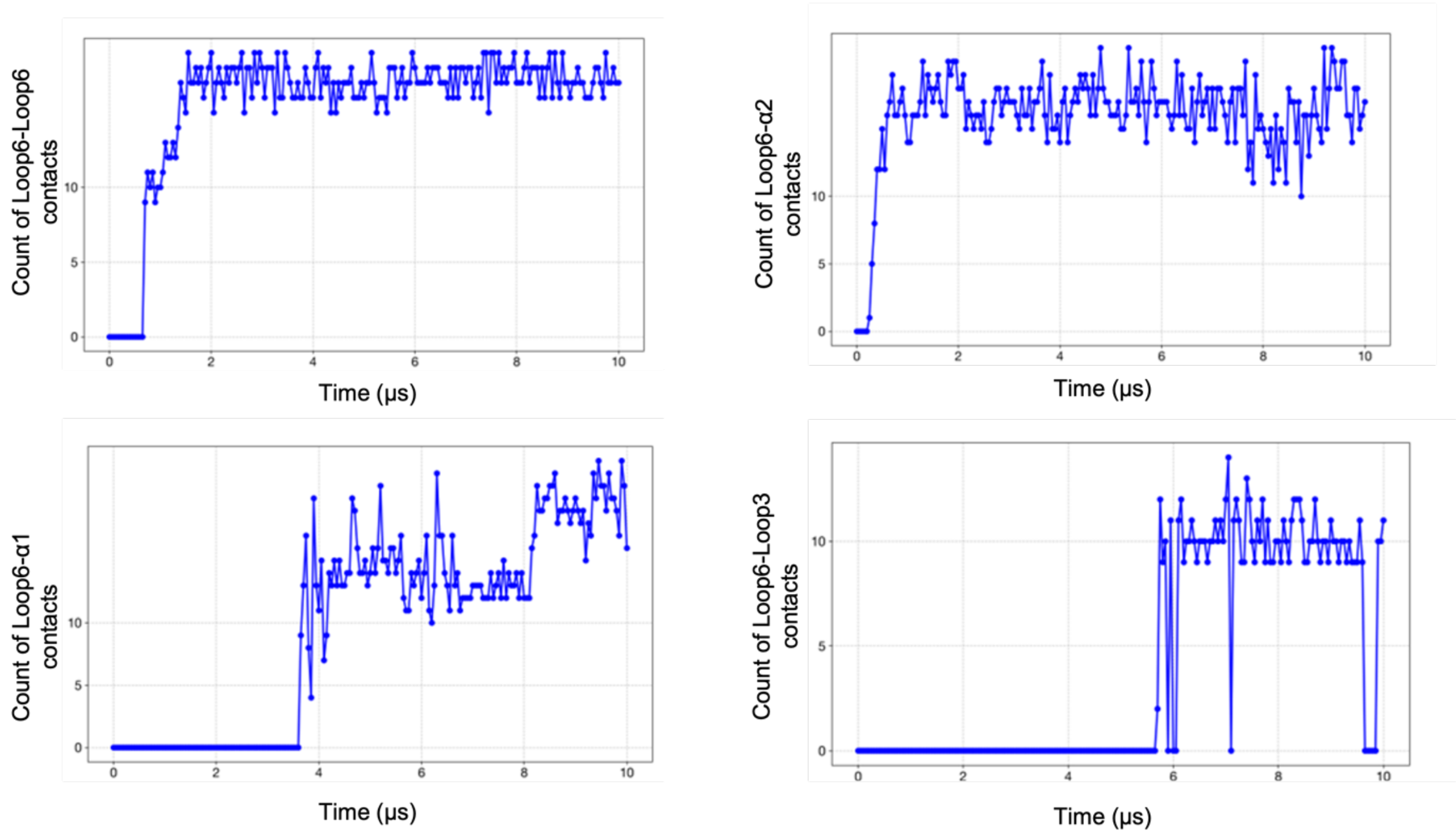


**Appendix Figure S12: Time evolution of the top binding poses interactions forming the interface.** The most probable region to be at the interface i.e, Loop 6 formed interactions early on the simulation leading to the clustering. Other regions which constitute the periphery of the interface formed interactions later.

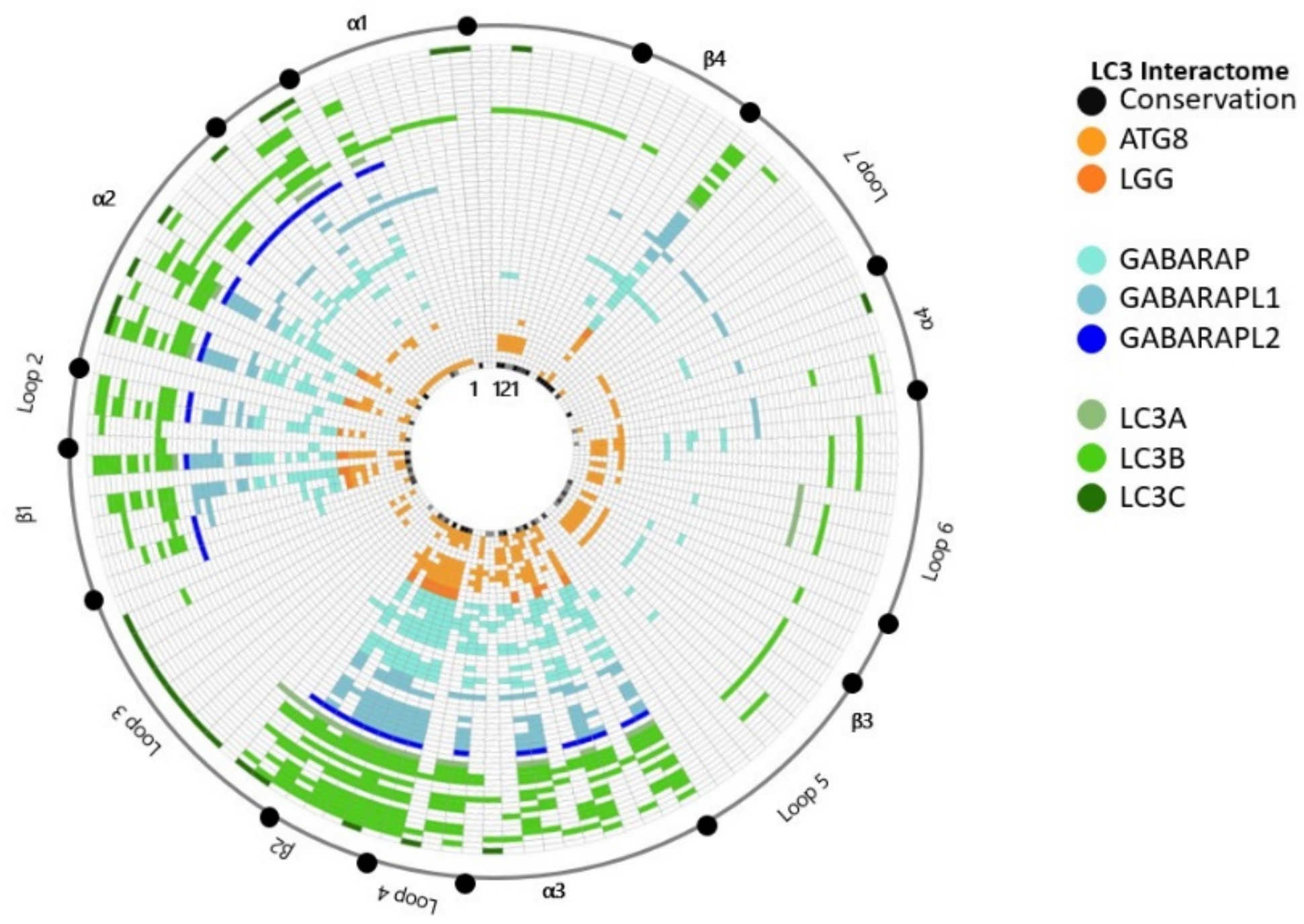


**Appendix Figure S13: LC3 protein-protein interaction map at residue level derived from experimental complexes.** A total of 46 bound crystal structures of all the orthologs of LC3 protein were included for understanding interfacial regions of LC3 complexes. The interface residues were calculated by taking a cutoff of 5Å. Radial plot displaying residue-wise information from available bound crystal structure data of all the orthologs of LC3B, illustrating the participation of each residue at the binding site.

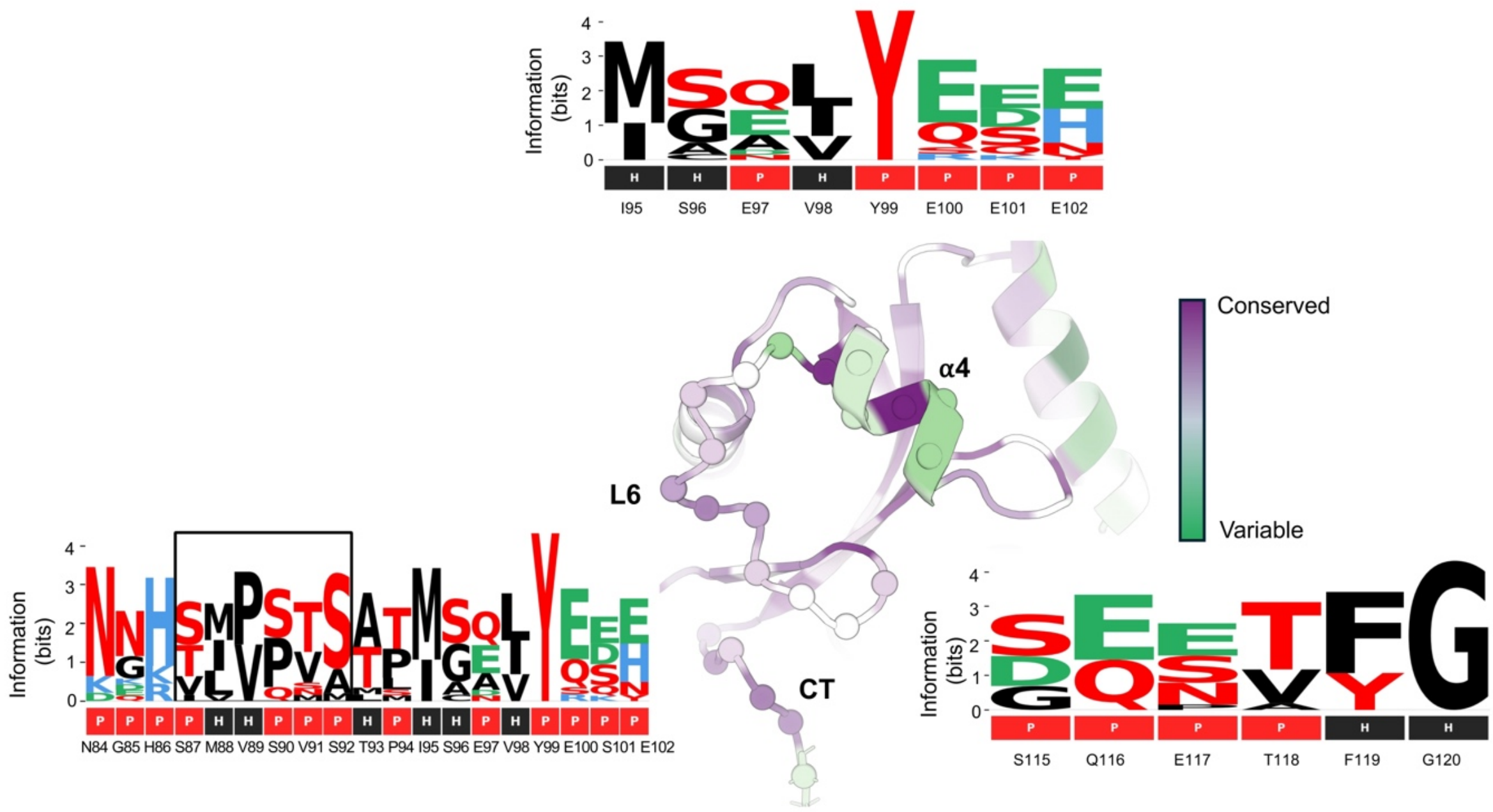


**Appendix Figure S14. Evolutionary conserveness of rear binding pocket.** The representative snapshot highlights the novel rear-side Loop6 pocket formed by Loop6-α4 in LC3B, identified as a key clustering interface alongside CT residues. Conservation scores mapped onto the structure indicate that this region is moderately to highly conserved across Atg8-family proteins supporting its functional relevance. A corresponding logo plot illustrates the residue distribution at each site, revealing a defined compositional pattern across Loop6, α4, and CT segments**.** The SMVSVS motif is highlighted (black box) as a prominent feature within Loop6. The property annotation below the logo plots demonstrating an alternating H/P pattern in Loop6.

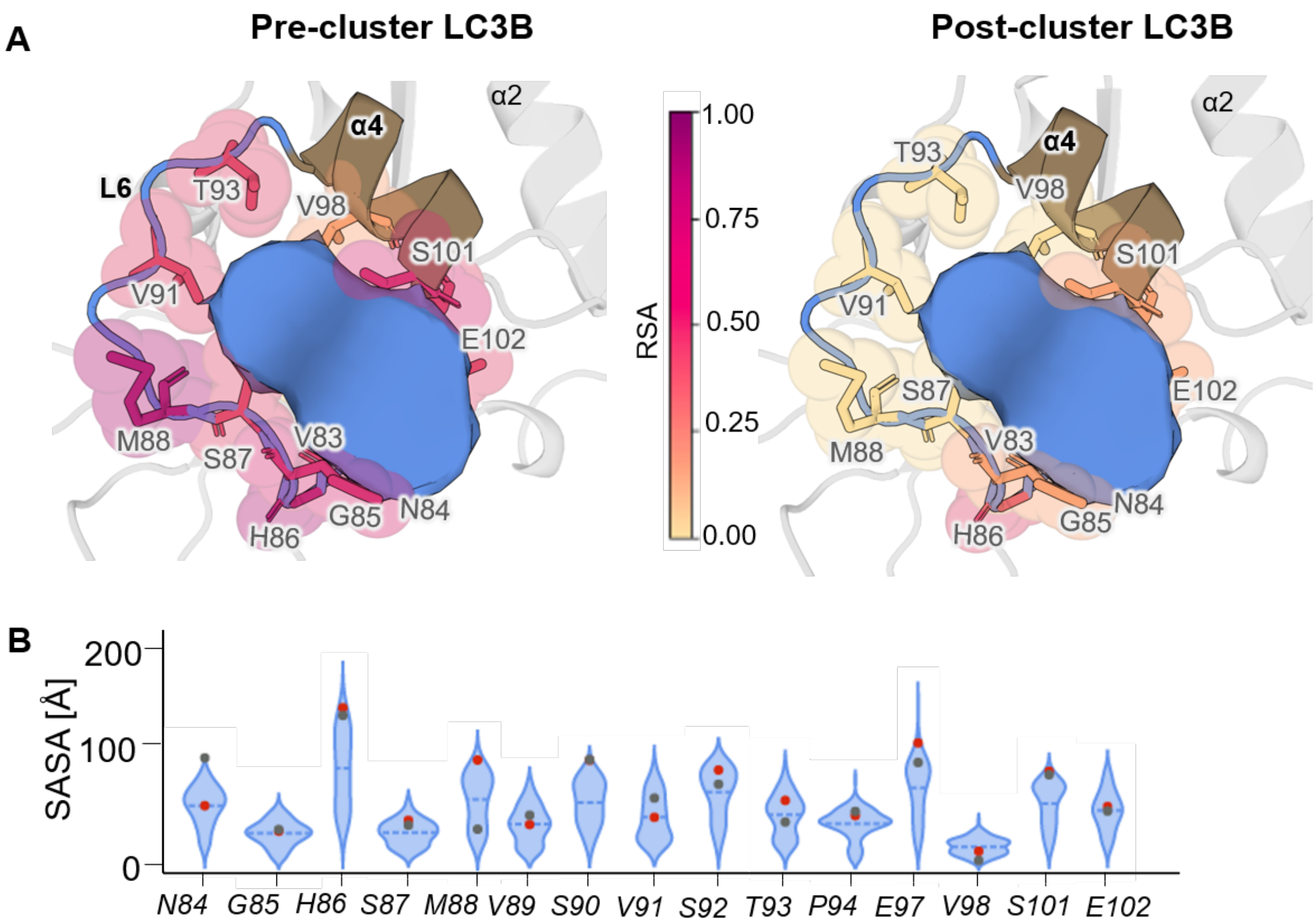


**Appendix Figure S15. Snapshots highlighting the relative residue accessibility in the most probable interface region between Loop 6 and α4 of LC3B.** A) Residue-specific relative solvent accessibility (RSA) differences (ΔRSA, unbound - bound) < 25% are mapped to emphasize their contribution to binding. (B) Violin plot showing the distribution of solvent-accessible surface area (SASA) for interface residues in LC3B dimers, demonstrating that most Loop 6 residues remain fully or partially exposed to facilitate clustering.

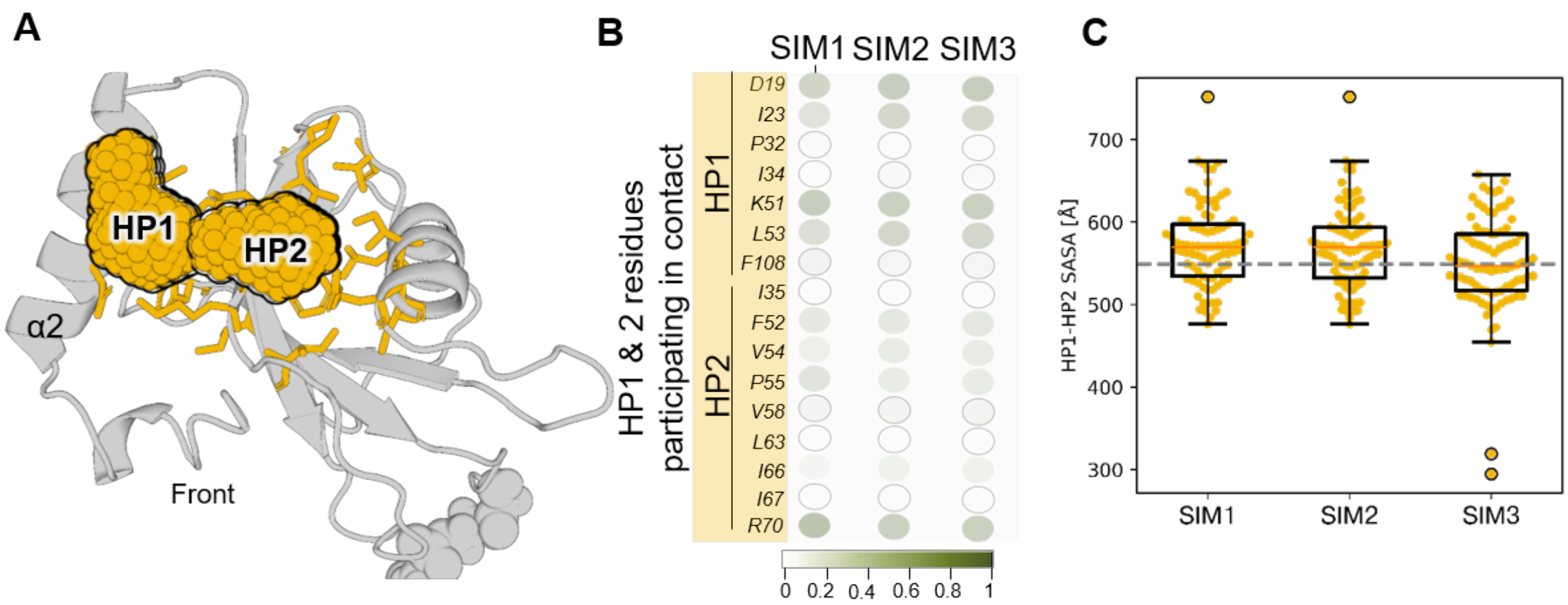


**Appendix Figure S16. Canonical binding pocket is non-overlapping with LC3B clustering interface.** (A) The two canonical hydrophobic pockets, HP1 and HP2, are highlighted in mustard color. (B) Binding pocket residues were analyzed for interactions contributing to clustering, but no such contacts were observed. (C) Surface accessibility calculations confirmed that the binding pocket remains exposed and available for potential binding.

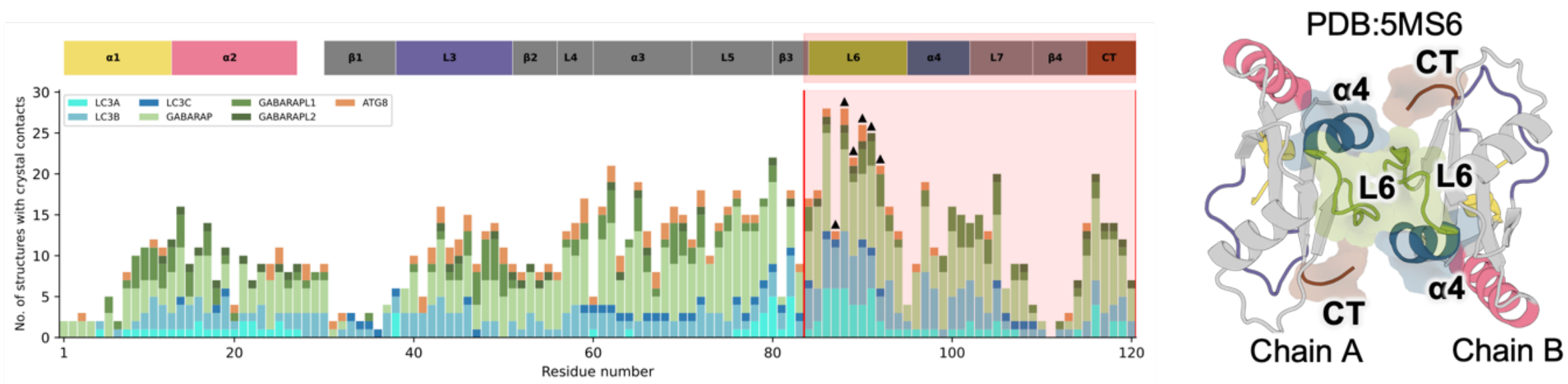


**Appendix Figure S17: Crystal packing reveals a recurrent LC3 self-association interface** (A) Percentage of crystal structures in which each residue participates in lattice contacts across 69 Atg8-family structures. Residues within Loop6 and helix α4 show the highest contribution to crystal packing. Secondary structure elements are annotated above the plot. The shaded region highlights the L6–α4–C-terminal (CT) interface (residues ~84–101 and 115–120). The black triangle marks the SMVSVS motif. (B) Representative crystal structure PDB: 5MS6 illustrating Loop 6 positioned at the dimer interface, consistent with its recurrent engagement in lattice contacts across structures.

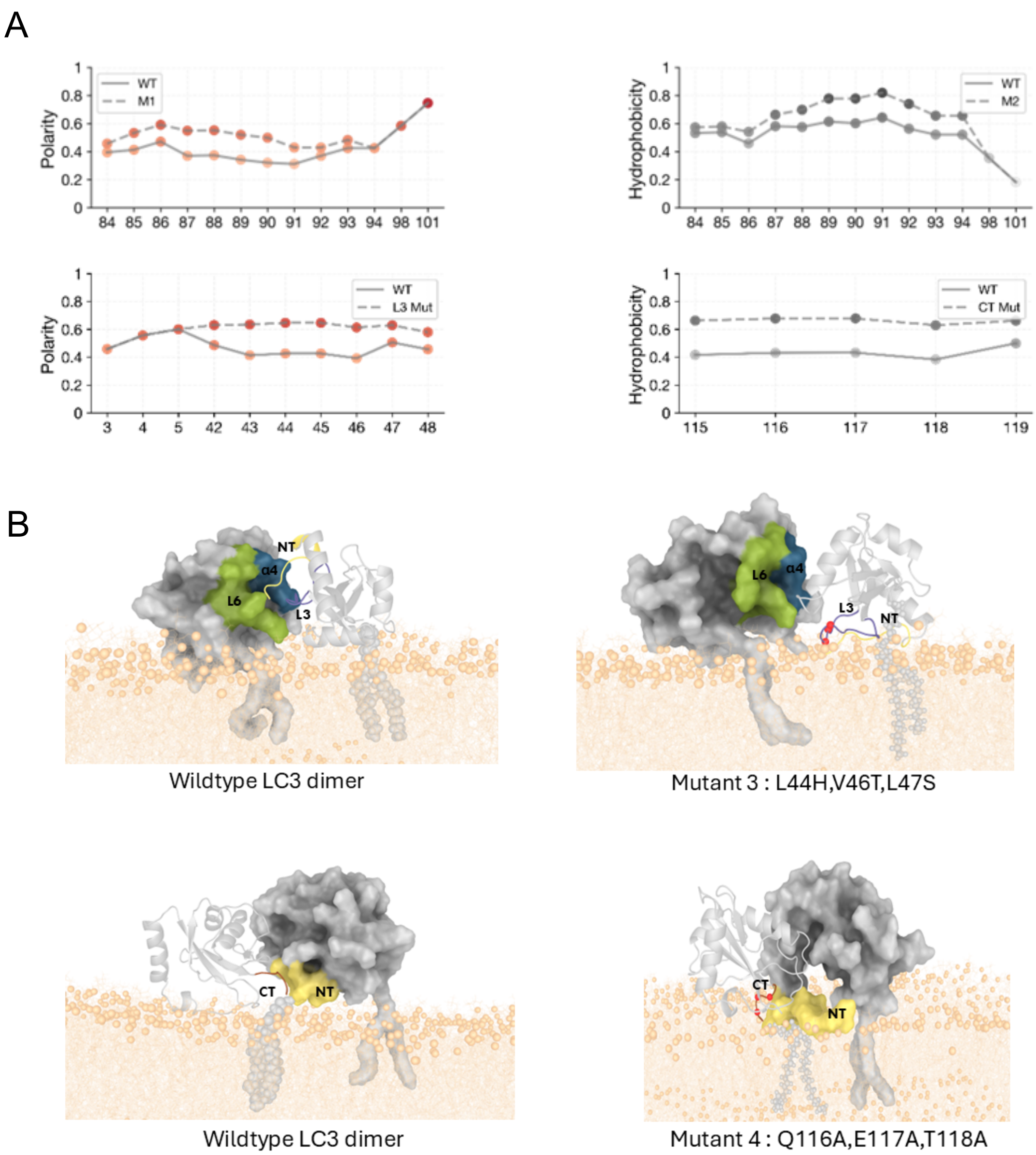


**Appendix Figure S18. In silico characterisation of the Loop 3 and C-terminal LC3B clustering mutants.** (A) Polarity and hydrophobicity profiles of wild-type (WT) and mutants across the regions flanking the mutation site. The quantitative line plot compares polarity (Grantham scale) and hydrophobicity (Kyte & Doolittle scale) profiles for the WT and each of the four mutants. For every mutation, values are plotted across the residues flanking the mutation site to visualise local physicochemical changes. Shifts between the profiles indicate alterations in polarity and hydrophobicity within the local sequence environment, highlighting how each mutation perturbs its immediate neighbourhood. (B) Final configurations of $LC3^{Loop3Mutant}$ after 650 ns of MD simulation, shown alongside $LC3^{WT}$ initiated from the same starting structure. In contrast to the Loop 6 mutants, the Loop 3 mutant did not exhibit dissociation between LC3B molecules. However, a clear reorientation of key interface residues was observed, suggesting that the Loop 3 mutation perturbs the geometry of the interaction surface without disrupting overall dimer association. (C) Final configurations of $LC3^{CterMutant}$ after 650 ns of MD simulation alongside the wild-type in the same starting configuration. The $LC3^{CterMutant}$ exhibited no dissociation between LC3B molecules.

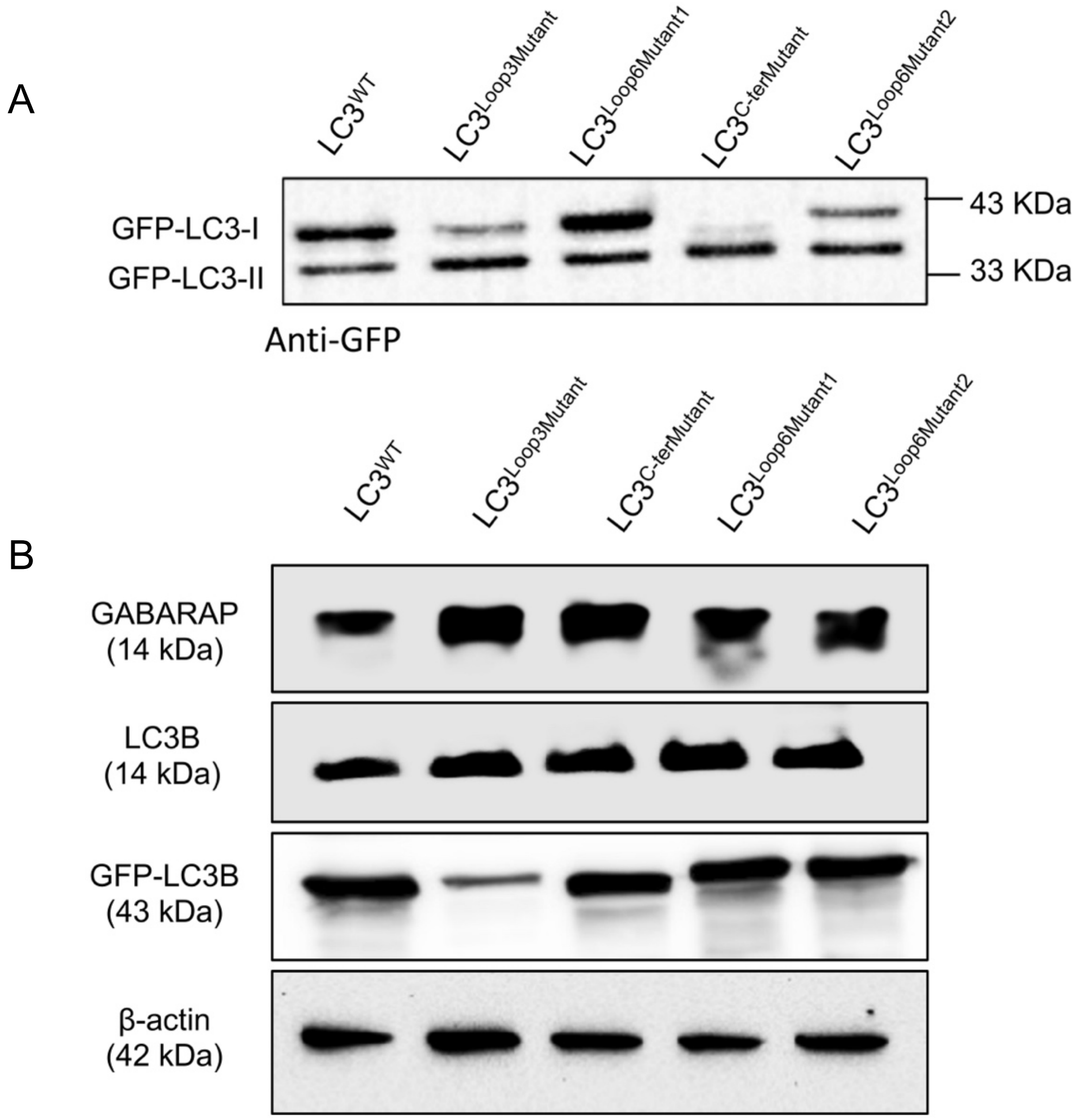


**Appendix Figure S19. Lipidation and endogenous ATG8 levels are preserved across GFP-LC3B variants.** (A) Western blot analysis of GFP-tagged LC3B wild-type protein and the four mutant proteins, performed to confirm lipidation and demonstrating the presence of two populations of LC3. (B) Immunoblot depicting the endogenous GABARAP and LC3B levels in host cells following transfection of the different GFP-LC3 variants. GABARAP and LC3 levels remained unchanged across all mutant variants.

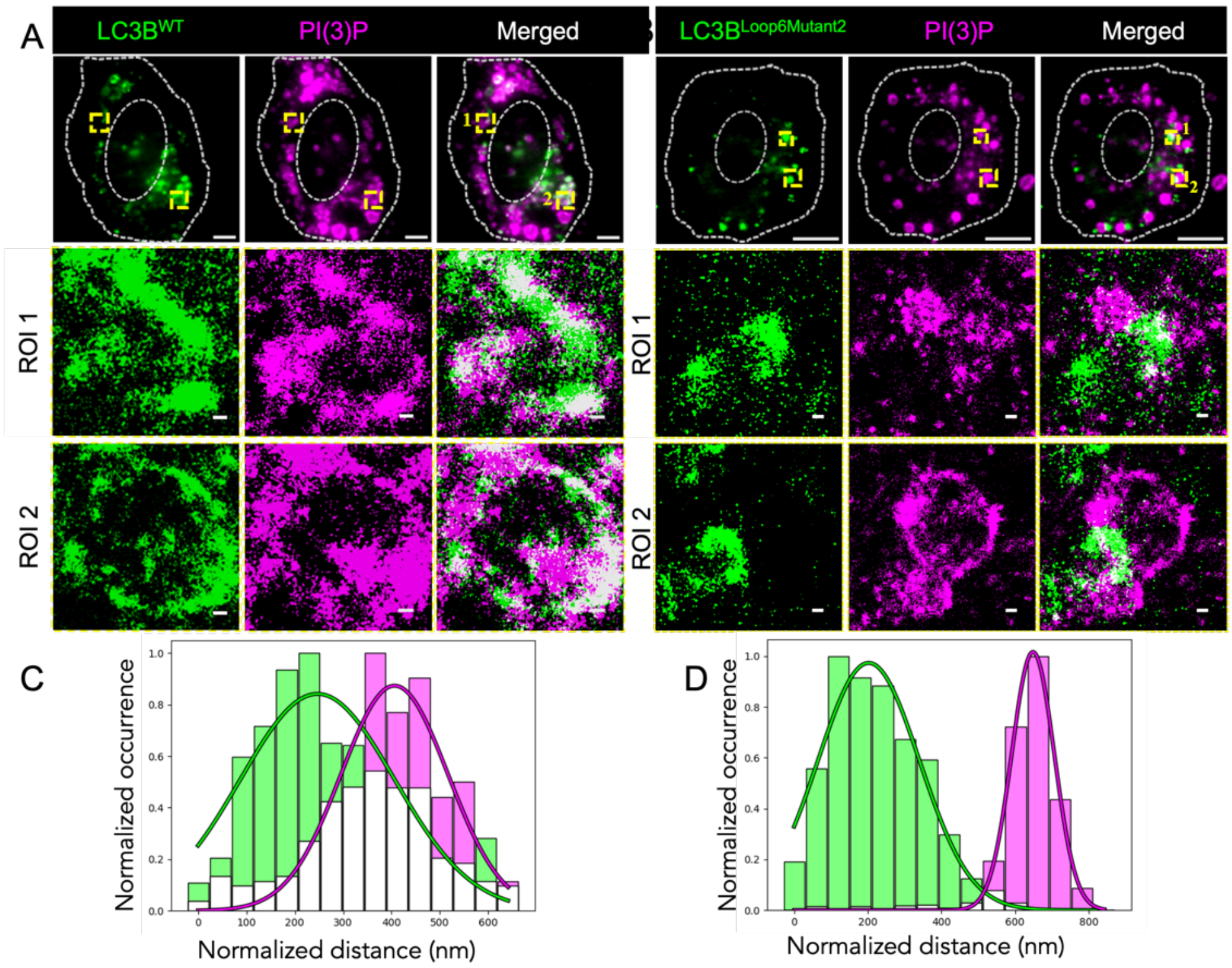


**Appendix Figure S20. Super-resolution imaging reveals diminished association of the LC3B$^{Loop6Mutant2}$ with PI3P-positive vesicles**. Sequential two-colour 2D STORM imaging of starved BS-C-1 cells overexpressing the mCherry-tagged 2×FYVE domain of HRS together with either EGFP-LC3B$^{WT}$ or EGFP-LC3B$^{Loop6Mutant2}$, followed by immunostaining against EGFP and mCherry. (A,B) Representative STORM images of cells expressing EGFP-LC3B$^{WT}$ (A) or EGFP-LC3B$^{Loop6Mutant2}$ (B). Insets highlight representative regions showing the overlap between autophagosomes and PI3P-positive vesicles. (C,D) Representative localization histograms depicting the overlap between 2×FYVE-HRS and EGFP-LC3B$^{WT}$ (C) or EGFP-LC3B$^{Loop6Mutant2}$ (D). Scale bars: 5 µm; inset scale bars: 200 nm.

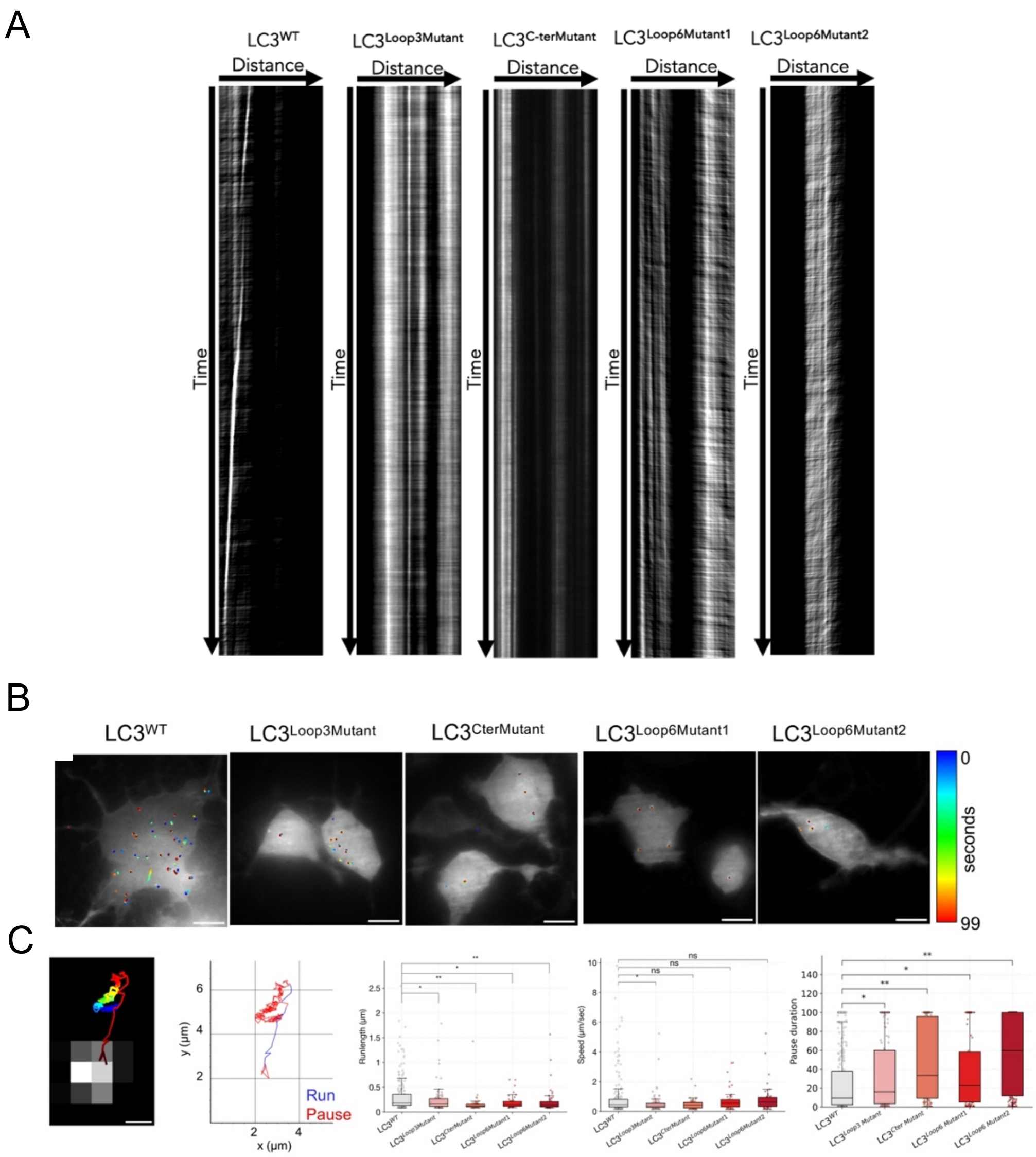


**Appendix Figure S21. Autophagosome motility is impaired in LC3B clustering mutants.** (A) Representative kymographs depicting the motility of eGFP-LC3B-positive autophagosome vesicles in Neuro-2a cells (600 fps). Kymograph analysis of TIRF imaging illustrates the movement patterns of autophagosomes labelled with LC3 constructs. Autophagosomes expressing $LC3^{WT}$ display robust long-range, directional transport along neurite-like cellular projections. In contrast, cells expressing mutant LC3 constructs exhibit markedly reduced motility. (B) Representative single-particle tracking trajectories (coloured lines) of EGFP–LC3B–positive autophagosomes in Neuro2A cells. Scale bar: 10 µm. Trajectories are colour-coded by time (T). (C) Quantitative analysis of autophagosome trafficking dynamics. Representative temporal colour-coded trajectory of an EGFP–$LC3B^{WT}$-positive autophagosome. Scale bar: 200 nm. Representative colour-coded EGFP–LC3B–positive autophagosome trajectory showing direct run phases in blue and confined pause phases in red. Distribution of run lengths for directed trajectories in wild-type and mutant LC3B. Distribution of the speed of run phases (direct trajectories) for the wild-type and mutant LC3. Distribution of pause duration for all trajectories for the wild-type and mutant LC3. Data are shown from 18 cells ($LC3^{WT}$), 22 cells ($LC3^{Loop3Mutant}$), 18 cells ($LC3^{CterMutant}$), 23 cells ($LC3^{Loop6Mutant1}$) and 21 cells ($LC3^{Loop6Mutant2}$) pooled from three independent experiments. All box plots are as described in the legend to Figure 5.

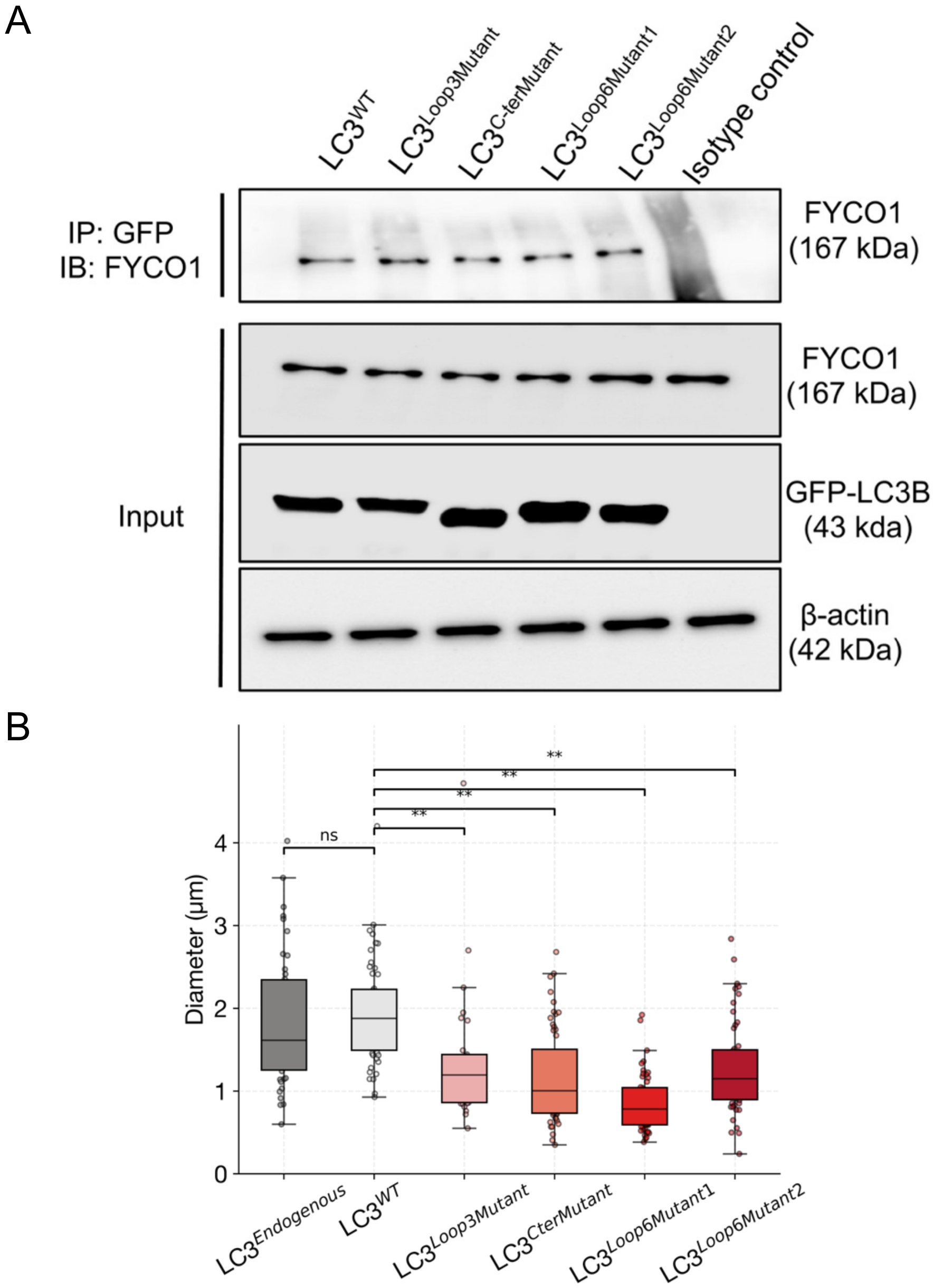


**Appendix Figure S22. FYCO1 binding and autophagosome size in cells expressing LC3 clustering mutants.** (A) Immunoblot showing the interaction of FYCO1 with GFP-tagged LC3 variants. Host cells were transfected with GFP-LC3 constructs for 36 h, followed by immunoprecipitation using an anti-GFP antibody and immunoblotting with an anti-FYCO1 antibody to assess FYCO1 association with the different LC3 variants. (B) Box plot comparing autophagosome size distributions in cells expressing wild-type and mutant LC3 constructs. Disruption of LC3 clustering results in significantly smaller autophagosomes compared with LC3WT, which exhibits a broader size distribution extending from approximately 800 nm to nearly 3 µm (two-sided Mann–Whitney U test; exact P values are provided in Dataset EV1). Data were obtained from 6–8 cells across two independent experiments. The box plot is defined as in the legend to Figure 5.

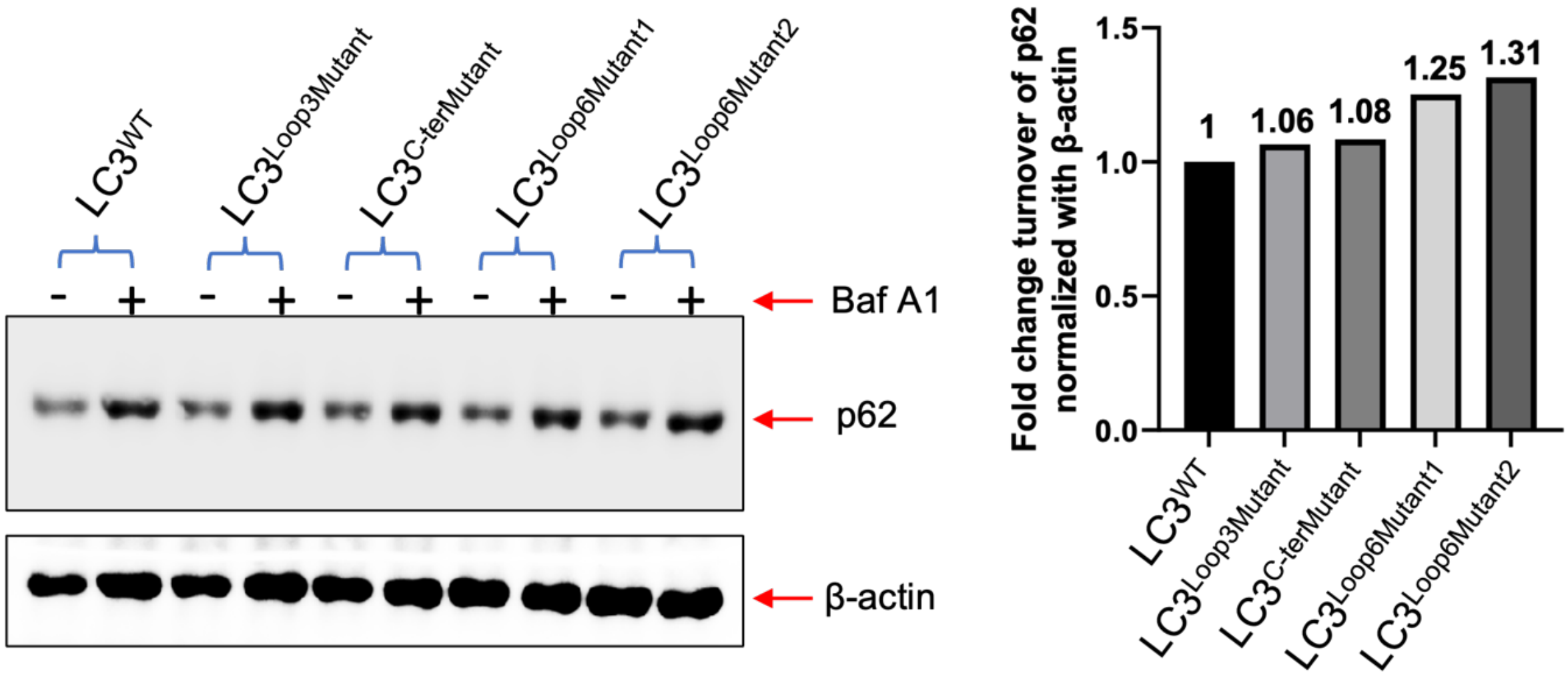


**Appendix Figure S23. Immunoblot demonstrating autophagy flux as p62 accumulation as a readout.** p62 turnover was assessed in cells treated with or without Baf A1 (25 nM) for 8 h. Immunoblotting of the cell lysate was performed with anti-p62 Ab.

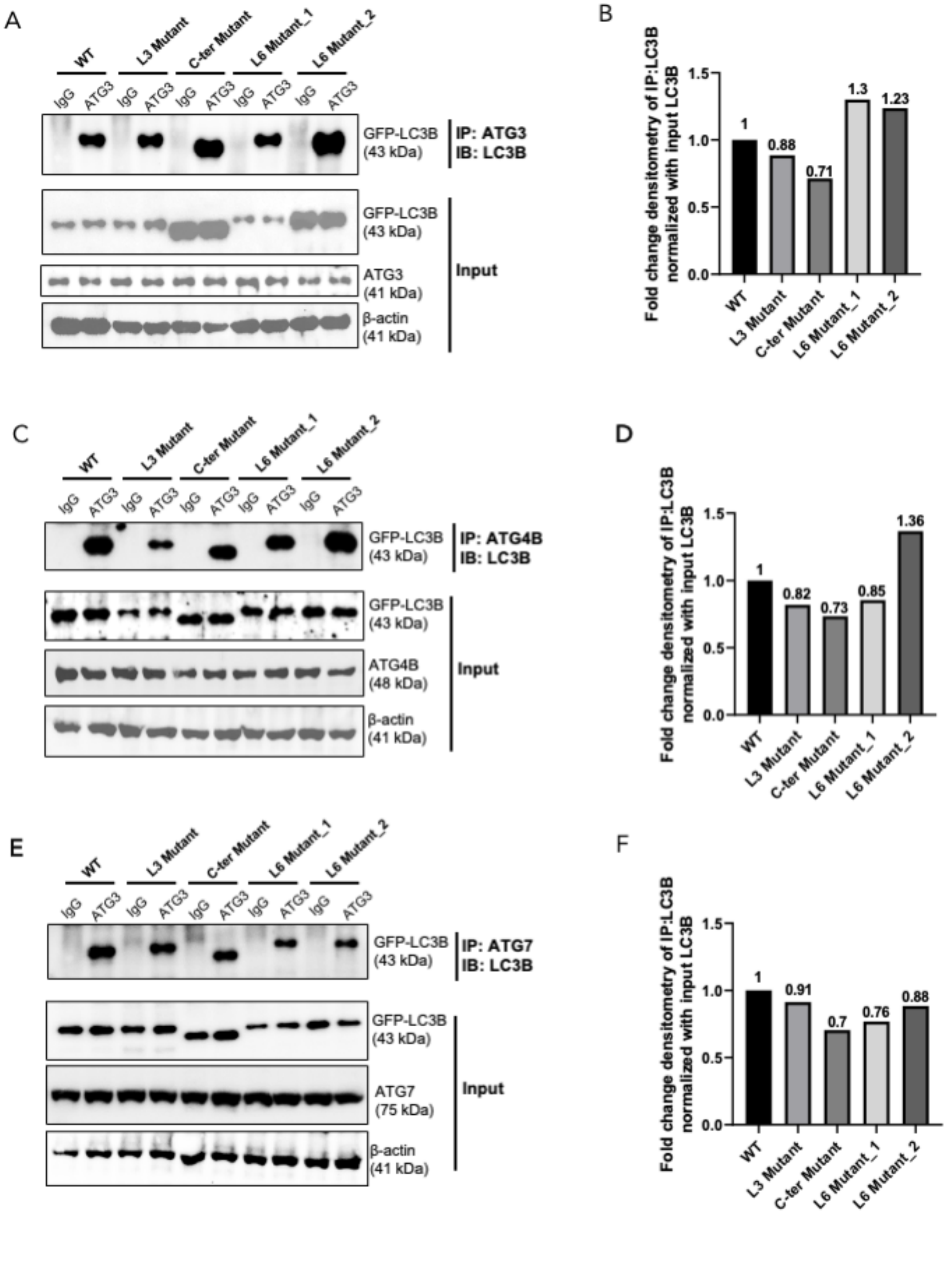


**Appendix Figure S24. Mutant LC3 had unaltered interaction with the autophagic machinery**. A, C, E. Immunoblot demonstrating the interaction of LC3 (WT) and its mutants with ATG3 (A), ATG4B (C), and ATG7 (E) in host A549 cells upon treatment with HBSS and Bafilomycin A1 (25 nM) for 8 h. B, D, F. Graph depicting the densitometry of the amount of GFP-LC3 immunoprecipitated with ATG3 (B), ATG4B (D) and ATG7 (F), normalized with the amount of GFP-LC3 expressed in host A549 cells, which has been transiently transfected.
