# Supplementary material for: LC3 forms functional nanoclusters on autophagosomes
Source: EMBO Rep. 2026 Sep 25;27(21):6139–65. doi: 10.1038/s44319-026-00931-0 (PMC13743770; doi:10.1038/s44319-026-00931-0)
Supplement: Supplementary file 2 [file 44319_2026_931_MOESM27_ESM.pdf]

Expanded View Figures

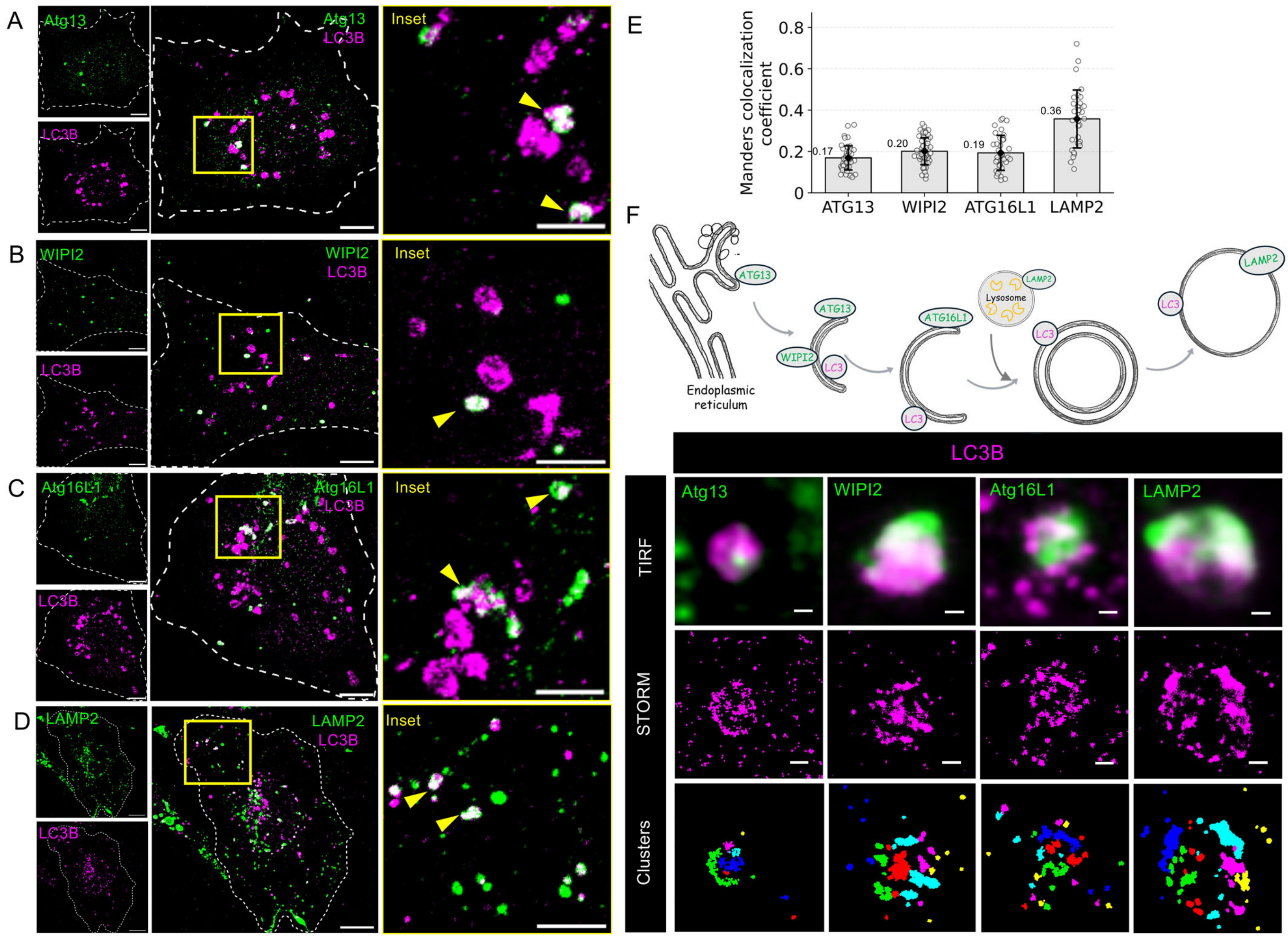

Figure EV1. LC3 forms nanoclusters on Atg13-, WIPI2-, ATG16L1-, and LAMP2-positive vesicles.

(**A–D**) Representative dual-color confocal images of LC3B (magenta) with ATG13, WIPI2, ATG16L1, or LAMP2 (green). Yellow boxes indicate enlarged regions highlighting double-positive vesicles. (**E**) Manders' colocalization coefficient quantifying colocalization of LC3 with ATG13, WIPI2, ATG16L1, and LAMP2. Data are shown as mean ± SD from 50 cells (ATG13), 54 cells (WIPI2), 34 cells (ATG16L1), and 36 cells (LAMP2), pooled from two independent experiments. (**F**) Schematic representation of autophagy pathway along with dual-color TIRF images of LC3 (magenta) with ATG13, WIPI2, ATG16L1, or LAMP2 (green), with corresponding STORM images showing the spatial organization of LC3 nanoclusters on marker-positive vesicles. Scale bars: 10 µm (full images), 5 µm (zoomed insets, **A–D**), and 500 nm (**F**).

 © The Author(s)

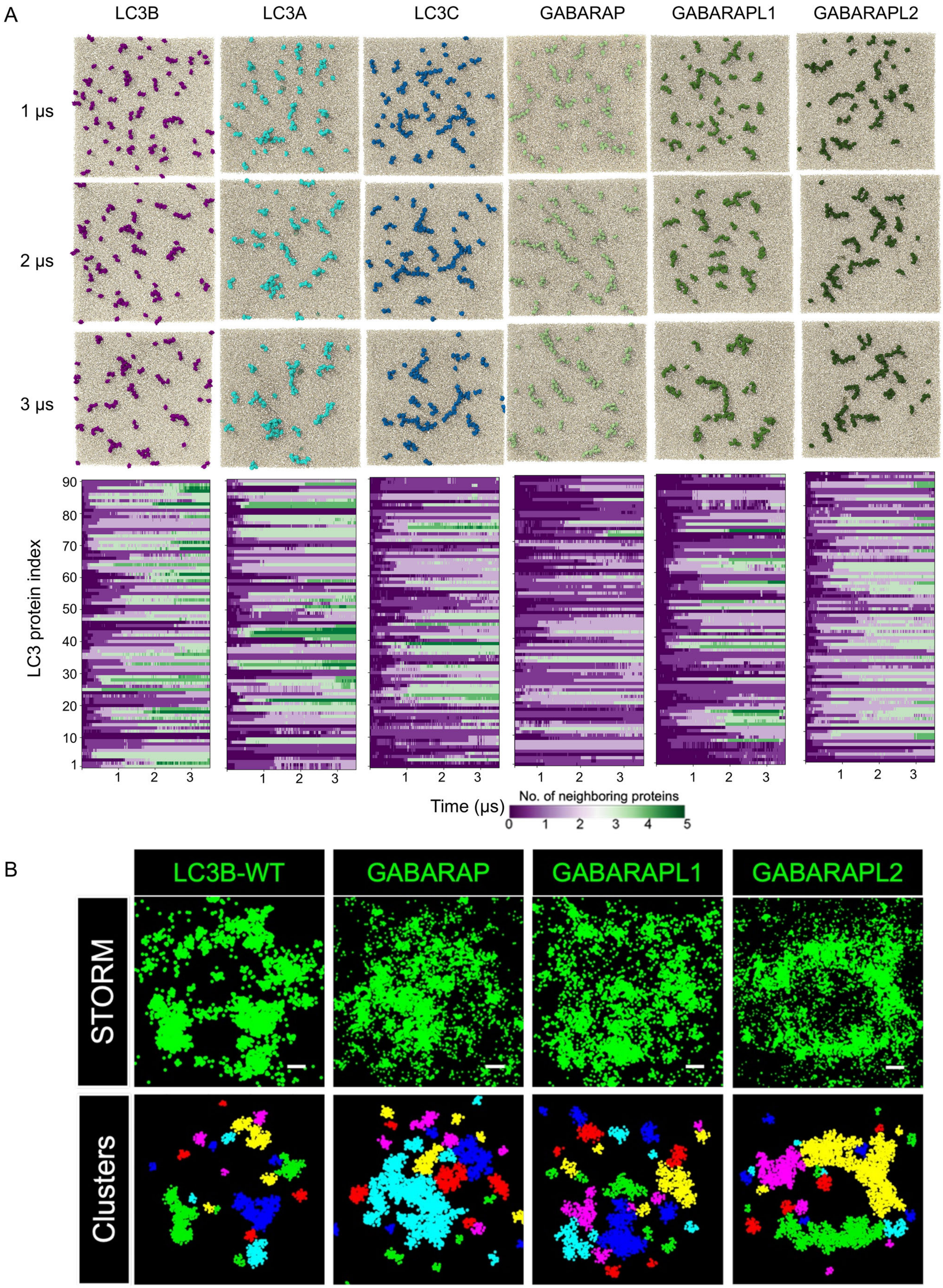
A
LC3B
LC3A
LC3C
GABARAP
GABARAPL1
GABARAPL2
1 μs
2 μs
3 μs
LC3 protein index
Time (μs)
No. of neighboring proteins
B
LC3B-WT
GABARAP
GABARAPL1
GABARAPL2
STORM
Clusters

Figure EV2. Simulations and STORM imaging reveal cluster formation of Atg8-family homologs.

(**A**) Representative snapshots from coarse-grained molecular dynamics simulations of the human Atg8-family homologs LC3A, LC3B, LC3C, GABARAP, GABARAPL1, and GABARAPL2 (GATE16) at 1, 2, and 3 µs. The LC3B 1 µs snapshot is reproduced from Fig. 1C for comparison with the other LC3 homologs. All homologs exhibit spontaneous membrane-associated clustering qualitatively similar to LC3B, indicating that clustering is a conserved feature of the Atg8 family rather than a property unique to LC3B. The lower panel shows a comparative analysis of clustering organization across the six homologs. Heatmaps show the number of protein–protein interfaces formed by each monomer over time, revealing distinct clustering topologies despite the shared propensity to self-associate. GABARAP predominantly forms linear assemblies characterized by two interfaces per molecule, whereas LC3B displays more heterogeneous and interconnected interaction networks. (**B**) Representative STORM images of individual autophagosomes from starved BS-C-1 cells transiently expressing LC3B, GABARAP, GABARAPL1, and GABARAPL2, demonstrating that all homologs form discrete nanoscale clusters on autophagosomes in cells. Scale bar:200 nm.

 © The Author(s)

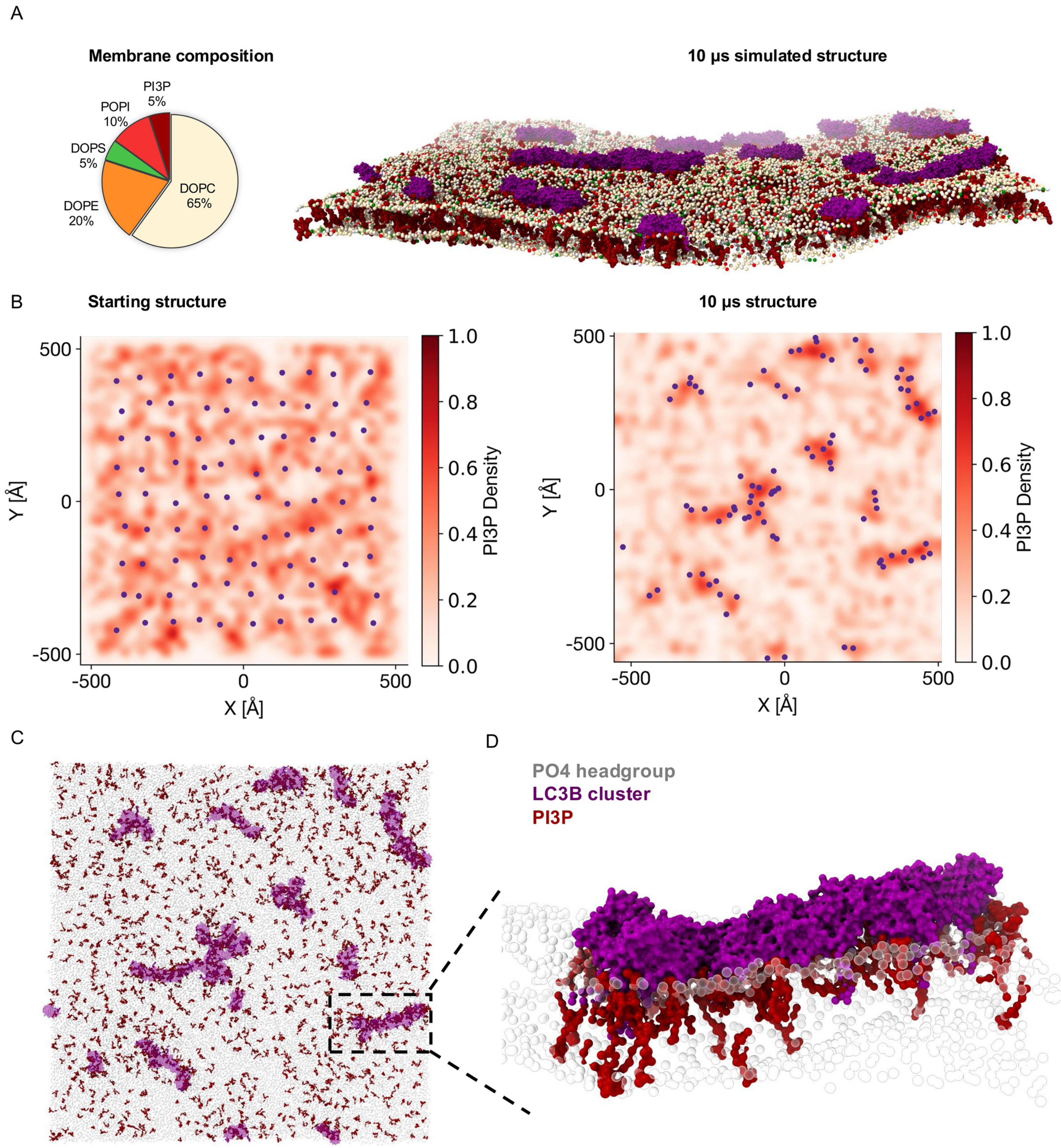

Figure EV3. PI3P enrichment around LC3 clusters in coarse-grained simulations.

(**A**) Simulation setup and representative membrane configuration after 10 μs showing LC3 clusters (purple) on a mixed lipid bilayer. (**B**) Probability density maps of PI3P projected onto the membrane plane. Purple dots indicate LC3 positions. Regions occupied by LC3 clusters coincide with areas of elevated PI3P density. (**C**) Top view highlighting the spatial colocalization of LC3 clusters (purple) and PI3P lipids (red). (**D**) Zoomed view showing accumulation of PI3P headgroups beneath membrane-bound LC3 assemblies.
