# Supplementary material for: LC3 forms functional nanoclusters on autophagosomes
Source: EMBO Rep. 2026 Sep 25;27(21):6139–65. doi: 10.1038/s44319-026-00931-0 (PMC13743770; doi:10.1038/s44319-026-00931-0)
Supplement: Supplementary file 3 [file 44319_2026_931_MOESM2_ESM.pdf]

EMBO reports

# LC3 forms functional nanoclusters on autophagosome

Saman Fatihi, Deepak Khushalani, Joydipta Kar, Sourav Ghosh, Aayushi Singh, Suvapriya Roy, Anirban Banerjee, Nitin Mohan, and Lipi Thukral

*Corresponding author(s): Lipi Thukral (lipi.thukral@igib.in) , Nitin Mohan (nitinm@iitk.ac.in)*

**Review Timeline:**

| | |
|---|---|
| Submission Date: | 10th Dec 25 |
| Editorial Decision: | 5th Feb 26 |
| Revision Received: | 18th Jun 26 |
| Editorial Decision: | 15th Jul 26 |
| Revision Received: | 5th Aug 26 |
| Accepted: | 27th Aug 26 |

*Editor: Martina Rembold*

## Transaction Report:

(Note: With the exception of the correction of typographical or spelling errors that could be a source of ambiguity, letters and reports are not edited. Depending on transfer agreements, referee reports obtained elsewhere may or may not be included in this compilation. Referee reports are anonymous unless the Referee chooses to sign their reports.)

Dear Dr. Thukral

Thank you for the submission of your manuscript to EMBO reports. I apologize for the delay in handling it, but we have now received the full set of referee reports as well as referee cross-comments that are all pasted below.

As you will see, the referees acknowledge that the findings are potentially interesting but they also have a number of concerns and suggestions how to further strengthen your data and conclusions, which need to be addressed. During cross-commenting referee #1 noted that "[...] LC3 is the only reliable marker for autophagosome membranes, so it's difficult to ask for another marker in Fig 2G.". Referee #2 emphasized that "The most important concern I have is this one: "STORM visualizes clusters of 1000 or more LC3B on a scale of 150-200 nm, which is far more massive than the clusters simulated in the MD. Yet the term cluster is used as if these are the same thing. They are actually completely different. The authors should use clear terminology to separate these things. Then they need to explain how the two different scales are related to each other."

Given the positive evaluation and constructive comments I would like to invite you to revise your manuscript with the understanding that the referee concerns must be fully addressed and their suggestions taken on board. Please address all referee concerns in a complete point-by-point response. Acceptance of the manuscript will depend on a positive outcome of a second round of review. It is EMBO reports policy to allow a single round of major revision only and acceptance or rejection of the manuscript will therefore depend on the completeness of your responses included in the next, final version of the manuscript.

We realize that it is difficult to revise to a specific deadline. In the interest of protecting the conceptual advance provided by the work, we recommend a revision within 3 months (8th May 2026). Please discuss the revision progress ahead of this time with the editor if you require more time to complete the revisions.

You can either publish the study as a short report or as a full article. For short reports, the revised manuscript should not exceed 27,000 characters (including spaces but excluding materials & methods and references) and 5 main plus 5 expanded view figures. The results and discussion sections must further be combined, which will help to shorten the manuscript text by eliminating some redundancy that is inevitable when discussing the same experiments twice. For a normal article there are no length limitations, but it should have more than 5 main figures and the results and discussion sections must be separate. In both cases, the entire materials and methods must be included in the main manuscript file.

IMPORTANT NOTE: we perform an initial quality control of all revised manuscripts before re-review. Your manuscript will FAIL this control and the handling will be DELAYED if the following APPLIES:
1) A data availability section providing access to data deposited in public databases is missing. If you have not deposited any data, please add a sentence to the data availability section that explains that.
2) Your manuscript contains statistics and error bars based on n=2. Please use scatter blots in these cases. No statistics should be calculated if n=2.

When submitting your revised manuscript, please review the submission guidelines (https://link.springer.com/journal/44319/submission-guidelines#cms-Revised-submissions) and carefully review the instructions that follow below. Failure to include requested items will delay the evaluation of your revision.

1) a .docx formatted version of the manuscript text (including legends for main figures, EV figures and tables). Please make sure that the changes are highlighted to be clearly visible.

2) individual production quality figure files as .eps, .tif, .jpg (one file per figure). See hhttps://media.springernature.com/original/springer-cms/rest/v1/content/27825798/data/v1 for more info on how to prepare your figures.

3) We replaced Supplementary Information with Expanded View (EV) Figures and Tables that are collapsible/expandable online. A maximum of 5 EV Figures can be typeset. EV Figures should be cited as 'Figure EV1, Figure EV2" etc... in the text and their respective legends should be included in the main text after the legends of regular figures.

- For the figures that you do NOT wish to display as Expanded View figures, they should be bundled together with their legends in a single PDF file called *Appendix*, which should start with a short Table of Content. Appendix figures should be referred to in the main text as: "Appendix Figure S1, Appendix Figure S2" etc. See 'Expanded View' section of the submission guidelines.

- Additional Tables/Datasets should be labeled and referred to as Table EV1, Dataset EV1, etc. Legends have to be provided in a separate tab in case of .xls files. Alternatively, the legend can be supplied as a separate text file (README) and zipped together with the Table/Dataset file.

4) a .docx formatted letter INCLUDING the reviewers' reports and your detailed point-by-point responses to their comments. As

part of the EMBO Press transparent editorial process, the point-by-point response is part of the Review Process File (RPF), which will be published alongside your paper.

5) a complete author checklist, which you can download from our author guidelines. Please insert information in the checklist that is also reflected in the manuscript. The completed author checklist will also be part of the RPF.

6) Please note that all corresponding authors are required to supply an ORCID ID for their name upon submission of a revised manuscript (<https://orcid.org/>), which can be linked to your profile in the manuscript submission system.

7) Before submitting your revision, primary datasets produced in this study need to be deposited in an appropriate public database. Please remember to provide a reviewer password if the datasets are not yet public. The accession numbers and database should be listed in a formal "Data Availability" section placed after Methods. Please note that the Data Availability Section is restricted to new primary data that are part of this study. * Note - All links should resolve to a page where the data can be accessed. *

If your study has not produced novel datasets, please mention this fact in the Data Availability Section.

8) At EMBO Press we ask authors to provide source data for the main manuscript figures. You will receive a separate email with instructions for providing source data with your revised manuscript, including how to upload and organize the files.

9) Our journal also encourages inclusion of *data citations in the reference list* to directly cite datasets that were re-used and obtained from public databases. Data citations in the article text are distinct from normal bibliographical citations and should directly link to the database records from which the data can be accessed. In the main text, data citations are formatted as follows: "Data ref: Smith et al, 2001" or "Data ref: NCBI Sequence Read Archive PRJNA342805, 2017". In the Reference list, data citations must be labeled with "[DATASET]". A data reference must provide the database name, accession number/identifiers and a resolvable link to the landing page from which the data can be accessed at the end of the reference.

10) Regarding data quantification, the following points must be specified in each figure legend:

- the name of the statistical test used to generate error bars and P values,

- the number (n) of independent experiments (please specify technical or biological replicates) underlying each data point,

- the nature of the bars and error bars (s.d., s.e.m.),

- If the data are obtained from n Program fragment delivered error ``Can't locate object method "less" via package "than" (perhaps you forgot to load "than"?) at //ejpvfs23/sites23b/embor_www/letters/embor_decision_revise_and_review.txt line 54.' 2, use scatter blots showing the individual data points.

Discussion of statistical methodology can be reported in the materials and methods section, but figure legends should contain a basic description of n, P and the test applied.

- Please also include scale bars in all microscopy images.

11) The journal requires a statement specifying whether or not authors have competing interests (defined as all potential or actual interests that could be perceived to influence the presentation or interpretation of an article). In case of competing interests, this must be specified in your disclosure statement.

12) All Materials and Methods need to be described in the main text using our 'Structured Methods' format, which is required for all research articles. According to this format, the Methods section includes a Reagents and Tools Table (listing key reagents, experimental models, software and relevant equipment and including their sources and relevant identifiers) followed by a Methods and Protocols section describing the methods using a step-by-step protocol format. The aim is to facilitate adoption of the methodologies across labs. More information on how to adhere to this format as well as a downloadable template (.docx) for the Reagents and Tools Table can be found in our author guidelines.

We would also welcome the submission of cover suggestions, or motifs to be used by our Graphics Illustrator in designing a cover.

As part of the EMBO publication's Transparent Editorial Process, EMBO reports publishes online a Review Process File (RPF) to accompany accepted manuscripts. This File will be published in conjunction with your paper and will include the referee reports, your point-by-point response and all pertinent correspondence relating to the manuscript.

You are able to opt out of this by letting the editorial office know (emboreports@embo.org). If you do opt out, the Review Process File link will point to the following statement: "No Review Process File is available with this article, as the authors have

chosen not to make the review process public in this case."

I look forward to seeing a revised form of your manuscript when it is ready.

Yours sincerely,

Martina Rembold, PhD
Senior Editor
EMBO reports

==================

Referee #1:

In this manuscript, Fatihi et al. demonstrate that LC3B organizes into dense, spatially distinct nanoclusters enriched in specific PI3P lipids on the autophagosomal membrane. The authors support this model using a combination of molecular dynamics simulations and high-resolution imaging. They introduce functional mutations that disrupt LC3B self-association, showing that these mutations impair nanocluster formation, and further demonstrate consequences for autophagy-related processes, including FYCO1 association, autophagosomal trafficking, and pathogen clearance. Overall, the data were quantitative and largely convincing. This is a concise and impactful study that will be of broad interest to the field. Suggestions for improvement are outlined below.

1) A major conceptual issue is that the authors do not adequately address other Atg8 homologs. It remains unclear whether the observed clustering behavior is specific to LC3B or represents a general principle applicable to all human Atg8 family members. (Similarly, it could be helpful to consider whether this phenomenon extends to other species [e.g., yeast Atg8], although the other human homologs feel more critical to include). Coarse-grained MD simulations incorporating other Atg8 homologs would help define the scope of this finding. This issue is particularly important given that LC3B is dispensable for autophagy in mammalian systems, presumably due to redundancy among Atg8s. Relatedly, it would be valuable to know whether LC3B nanoclusters are composed exclusively of LC3B, or whether other LC3s or GABARAP family members are also present (This could be done with MD and/or high res imaging). Determining whether different Atg8 proteins mix within clusters or segregate into distinct domains would substantially increase the impact of the study.
2) The authors should provide more than a single simulation for Fig. 4B/C. For consistency with earlier figures, why not use coarse-grained simulations as in Fig. 1? It is not clear what additional insight was gained by switching from coarse-grained to atomistic simulations in this context.
3) For Fig. S21 (kymographs), quantification is needed, rather than a single example being shown. For example, speed and run length could be quantified.
4) It is not entirely clear why the LC3B mutants behave as dominant negatives. While this issue is not disqualifying, it is the main result that does not align with naïve expectations and would benefit from additional discussion or clarification.
5) The authors consider membrane thickness effects (Fig. 1E), but do not address membrane curvature. Do LC3B nanoclusters influence curvature, or does curvature affect cluster formation? The inner and outer membranes of the autophagosome are likely to present distinct physical environments that could differentially impact clustering.

Minor points
• The heterogeneity among interface contacts is reminiscent of ubiquitin-ubiquitin interactions (PMID: 37225996) and could be discussed.
• Line 218 / Fig. S14: It is not clear where the SMVSVS motif maps onto the sequence logo.
• Line 265: "Our positive control is wild-type LC3-eGFP..." should be corrected to "eGFP-LC3."
• Line 402: WIPI proteins do not contain FYVE domains; this should be corrected.

Referee #2:

Fatihi et al. report that LC3B forms nanoclusters that are important for autophagy. The strength of the paper is in the MD simulations and in the general concept, which makes sense. I have a great many questions about the experimental details and interpretations.
LC3B density: If LC3B is spaced 8 nm apart, that works out to 64 nm2 per LC3, or 1000 LC3D per island. How does this fit with other numbers in the literature?
LC3 self association. The authors state there are 43 solved structures showing an empty pocket. Many of these are crystallographic? The authors should check if any of them are occupied in self-associating lattice contacts. If not, why not?
LC3B is overexpressed in these studies on top of wild-type LC3B. The implications of a potential dominant negative mechanism

should be discussed. Transfection: show if there is equal expression and compare to endogenous levels. Are there changes in LC3A/B, GABARAPs?

STORM visualizes clusters of 1000 or more LC3B on a scale of 150-200 nm, which is far more massive than the clusters simulated in the MD. Yet the term cluster is used as if these are the same thing. They are actually completely different. The authors should use clear terminology to separate these things. Then they need to explain how the two different scales are related to each other.
This paper reminds me of one by Rod MacKinnon in PNAS, Nov. 2025, on higher order transient structures. (HOTS). The MacKinnon paper is much deeper. I don't expect the authors to do everything MacKinnon did, but do please cite the paper and compare the ideas. This paper is a good example of clarity in discussing nanoclusters of a few molecules as opposed to larger mesoscale entities.
Check grammar throughout, as there are frequent missing articles and wrong tenses. Font sizes in figures are almost unreadably small in some cases (mutant labels in Fig. 5, for ex.).
In Fig. 5 the phenotypes vary in ways that are hard to understand. Loop3 and Cter have no difference in number of clusters/autophagosome and less area/LC3B, while Loop6 mut 1 and 2 have fewer clusters/autophagosome and more area/LC3B. Why are the effects different on mesoscale clustering? Since the effects are so different, why are the effects on function the same? How does breaking the L6 interface lead to the single blob like clusters described? The rationalization offered is that there are also multivalent interactions with lipids. What if all the effects are actually mediated by changes in lipid co-clustering?
All mutants have less track displacement and less FYCO1 colocalization. FYCO1 binding- the authors should confirm FYCO1 LIR binding to LC3 is unaffected using purified proteins.
Fig. S20 show large unexplained differences in conjugation efficiency. Methods for Fig. S20 are not explained. Was autophagy induced, to drive LC3-II formation? L6 M1 has lower conjugation but other mutants have higher. The differences don't correlate with the phenotypes but are in some cases large, especially for the C term mutant (which is not surprising given its location). More thought should be given to the possiblility that the phenotypes relate to conjugation efficiency instead of nanoclustering. The authors should show conjugation is equal under the conditions of the experiments in Fig. 5 and 6.

Analysis of movies 10-14- there are many static and dynamic objects. It was not clear to me how some of the static objects in the mutant analyses were chosen for analysis. The apparent mutational loss of function could be an artifact of biased selection of objects for analysis, if I understand what the authors did.

It is very interesting that PI3P and LC3B clusters coincide. This seems to be one of the stronger conclusions of the paper but the implications are not as analyzed and discussed as thoroughly as they could be.
line 403. Wrong. WIPI proteins do not contain FYVE "motifs".
In the end, I am not clear about what the nanoclusters are actually doing. The discussion should be more forthright and specific about limitations. For ex, discussion, line 414, "For instance, in the selective autophagy of specific 414 organelles or protein aggregates, it may guide the autophagic machinery to the exact location 415 where it is needed." What does "it" refer to? I assume this refers to the nanocluster. How do the authors imagine that a nanocluster can guide the autophagic machinery to the exact location where it is needed, in a way that individual or randomly distributed molecules could not?

Referee #3:

In this study, the authors highlight the functional importance of LC3B nanoclustering in autophagy. Combining molecular dynamics simulations with super-resolution microscopy, they report that LC3B self-assembles into ~150 nm "islands" on autophagosome membranes with a preference for PI3P. They further identify a hydrophobic-polar clustering motif; mutations in this region disrupt LC3B clustering, weaken FYCO1 association, and, upon loss of LC3B nanoclustering, significantly compromise autophagic entrapment of Streptococcus pneumoniae, resulting in elevated intracellular bacterial survival. This is an interesting study for a broad audience; however, the manuscript needs a few clarifications to support its claims, particularly regarding the role of LC3B in the late maturation stage.

Comments

1. The manuscript states that LC3 nanoclusters appear as early as WIPI2-positive phagophores and persist through LAMP2-positive autolysosomes, implying that clusters accompany autophagosomes throughout maturation. This claim should be supported by quantitative co-localization (and ideally temporal analysis) with additional autophagy markers across stages (e.g., initiation/elongation, closure, fusion), not limited to WIPI2 and LAMP2.

2. Figure 2G: It is unclear whether the large circular structures are bona fide autophagosomes. Please include three-color imaging with a well-established autophagosomal membrane marker to confirm that the structures are autophagosomes.

Sometimes a larger lipid droplet may appear, which may contain PI(3)P or nonspecific binding. It looks very different compared to Figure 2A.

3. The authors propose that LC3 clusters maintain a relatively constant size while increasing in number. If the intended interpretation is that LC3B recruitment to autophagosomes is progressive, further evidence is needed.
An alternative explanation is that larger structures may be lower-density than the initial cluster and/or dissipate over time, leading to sparser distributions; this possibility should be tested and discussed.

4. Please include autophagy flux data using WB for all the LC3B mutants.
5. Figure 5: Please use consistent zoom/magnification (and scale bars) across all panels to enable direct visual comparison.
6. References: The references are not up to date; there are studies in which other groups observed LC3B clustering that need to be cited. (e.g., Science Advances 9 (39), eadh4094),
7. Statistics/replicates: Several figures require error bars, statistical tests, and clear reporting of n (including biological replicates). Please specify the number of independent experiments and the statistical methods used in the figure caption.

**Point-by-point Response to Editor's and Reviewers' Comments**

In the following, we address the comments (in *italics*) point by point and highlight changes made in the revised manuscript (in red).

**Title:**" LC3 forms functional nanoclusters"
**Manuscript #**: EMBOR-2025-63271V1

**Authors**: Fatihi et. al.,

**Editor comments**

*As you will see, the referees acknowledge that the findings are potentially interesting, but they also have a number of concerns and suggestions how to further strengthen your data and conclusions, which need to be addressed.*

**Our response: We thank you for the careful evaluation of our manuscript and for the constructive comments and suggestions provided by the referees. We appreciate that the reviewers found the findings potentially interesting, and we have now addressed the concerns to further strengthen the manuscript.**

*During cross-commenting referee #1 noted that "[...] LC3 is the only reliable marker for autophagosome membranes, so it's difficult to ask for another marker in Fig 2G."*

**Our response: We thank the reviewers for the cross-commenting exercise. We agree with Reviewer 1 that LC3 is one of the most extensively used and reliable markers for autophagosome membranes. Nevertheless, to further strengthen the interpretation of the observed LC3-positive structures, we performed additional colocalization analyses with multiple autophagy-stage markers, including ATG13 and ATG16L1. These complementary experiments support the conclusion that the LC3 assemblies analyzed in this study correspond to bona fide autophagic structures at distinct stages of autophagosome biogenesis and maturation.**

*Referee #2 emphasized that "The most important concern I have is this one: "STORM visualizes clusters of 1000 or more LC3B on a scale of 150-200 nm, which is far more massive than the clusters simulated in the MD. Yet the term cluster is used as if these are the same thing. They are actually completely different. The authors should use clear terminology to separate these things. Then they need to explain how the two different scales are related to each other."*

**Our Response: We thank the reviewer for raising this important conceptual point regarding the use of the term "cluster" across different spatial scales. We agree that the clusters observed in the MD simulations and the larger structures visualized by STORM cannot be interpreted as identical physical entities. Importantly, the coarse-grain simulations were designed to probe the underlying molecular interactions and weak multivalent interfaces that drive local LC3 self-association on membranes. We interpret these local oligomeric interactions as the molecular basis that may collectively contribute to the emergence of larger supramolecular membrane-associated assemblies observed experimentally. Thus, the two scales are related hierarchically rather than representing the same structure directly.**

**In the revised manuscript, we have therefore clarified the terminology throughout and now explicitly distinguish between (i) LC3 oligomers/clusters captured in simulations, which represent local protein–protein interaction-driven assemblies involving small numbers of LC3 molecules, and (ii) higher-order nanoscale LC3 assemblies observed experimentally by STORM imaging on autophagosomal membranes. This rationale has also discussed and updated in Revised manuscript on Pg 6.**

**We also believe there may have been some confusion in our earlier presentation, as we do not observe thousands of LC3 clusters. The gaussian distribution analysis of STORM-resolved LC3 assemblies (Figure 2F, also shown below) shows that the predominant LC3-positive structures are centered around ~150–300 nm. This typically corresponding to ~5-25 LC3 assemblies per autophagosome (Figure 2D, shown below). <u>However, only a small fraction of assemblies (~20 out of 377 detected clusters; ~5.5%) correspond to very large supramolecular LC3 assemblies</u>, indicating that these represent relatively rare higher-order organizational states rather than the dominant population.**

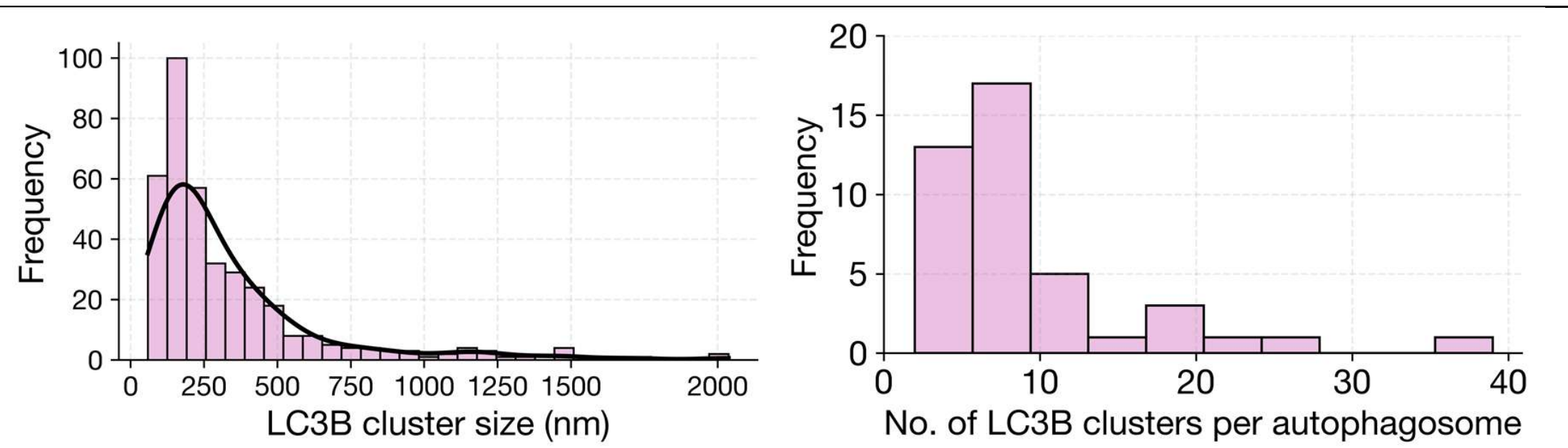


Figure 2F) Histogram and Gaussian fitting analysis of STORM-resolved LC3 assemblies showing that the predominant LC3-positive membrane-associated assemblies are centered around ~150–300 nm, representing the dominant organizational state observed across autophagosomes. Only a small fraction of assemblies (~5.5%) correspond to larger supramolecular LC3 structures. Figure 2D) Bar plot of the number of LC3B nanoclusters per autophagosome.

**Having said the above, as suggested by reviewer, these structures should be distinguishing from 'cluster' terminology, and we have consistently changed that in the manuscript to 'higher-order nanoscale LC3 assemblies' observed experimentally by STORM imaging on autophagosomal membranes. In the revised manuscript, we have also updated the Discussion section (Pg 14), and figure legends to more clearly explain the numbers of clusters.**

**Referee #1:**

*In this manuscript, Fatihi et al. demonstrate that LC3B organizes into dense, spatially distinct nanoclusters enriched in specific PI3P lipids on the autophagosomal membrane. The authors support this model using a combination of molecular dynamics simulations and high-resolution imaging. They introduce functional mutations that disrupt LC3B self-association, showing that these mutations impair nanocluster formation, and further demonstrate consequences for autophagy-related processes, including FYCO1 association, autophagosomal trafficking, and pathogen clearance. Overall, the data were quantitative and largely convincing. This is a concise and impactful study that will be of broad interest to the field. Suggestions for improvement are outlined below.*

**Our reply: We thank the reviewer for the careful reading and positive evaluation of our manuscript. We are encouraged that the reviewer found the study quantitative, convincing, and of broad interest to the field. We have carefully addressed all the comments and suggestions raised, including additional analyses related to LC3B homolog clustering, and describe each point in detail below.**

*R1.1) A major conceptual issue is that the authors do not adequately address other Atg8 homologs. It remains unclear whether the observed clustering behavior is specific to LC3B or represents a general principle applicable to all human Atg8 family members. (Similarly, it could be helpful to consider whether this phenomenon extends to other species [e.g., yeast Atg8], although the other human homologs feel more critical to include). Coarse-grained MD simulations incorporating other Atg8 homologs would help define the scope of this finding. This issue is particularly important given that LC3B is dispensable for autophagy in mammalian systems, presumably due to redundancy among Atg8s. Relatedly, it would be valuable to know whether LC3B nanoclusters are composed exclusively of LC3B, or whether other LC3s or GABARAP family members are also present (This could be done with MD and/or high res imaging). Determining whether different Atg8 proteins mix within clusters or segregate into distinct domains would substantially increase the impact of the study.*

**Our Response: We thank the reviewer for raising this important conceptual point regarding the generality of membrane-associated clustering across the human ATG8 family. In fact,**

**we have previously reported that the six human Atg8 homologs exhibit both distinct structural behavior as well as partially redundant structure-based functions (Jatana et al., Autophagy, 2020). Therefore, we agree that distinguishing whether clustering is unique to LC3B or represents a broader organizational principle is highly important.**

**As suggested by the reviewer, we performed comparative coarse-grained simulations for other five human ATG8 homologs (LC3A, LC3C, GABARAP, GABARAPL1, and GABARAPL2/GATE16) with similar simulation protocol. Interestingly, all homologs exhibited membrane-associated clustering behavior, suggesting that nanoscale assembly is likely a shared organizational feature across the ATG8 family (Figure S12). However, notable differences emerged in their assembly topology and clustering dynamics. In particular, GABARAP-family proteins tended to form more elongated and linear assemblies, whereas LC3-family proteins exhibited comparatively heterogeneous and distributed interaction networks (Supplementary Figure S13).**

**To further validate the simulation results, we performed additional STORM imaging experiments across GABARAP family members, including GABARAP, GABARAPL1, and GABARAPL2 (Supplementary Figure. S14), as the simulations revealed relatively distinct clusters for the GABARAP family proteins. Consistent with the simulations, all GABARAP homologs formed prominent membrane-associated nanoscale assemblies on autophagosomal structures. Interestingly, the GABARAP protein assemblies appeared more interconnected and less compact relative to LC3B, supporting the idea that although clustering is broadly conserved across LC3 family of human proteins, the detailed supramolecular organization and assembly topology may differ between homologs.**

**The reviewer also raised the important question of whether LC3 and GABARAP proteins can form heterotypic membrane-associated assemblies. To address this, we performed additional mixed 50:50 LC3B–GABARAP coarse-grained simulations and analyzed the temporal evolution of clustering behavior (Supplementary Figure S15). Interestingly, we observed frequent co-clustering of LC3B and GABARAP molecules within shared membrane-associated assemblies, suggesting that heterotypic clustering can occur. We are also excited to pursue this in future studies and answer the mechanism of how hetero assemblies may be involved in different aspects of autophagosome biogenesis. However, the detailed molecular mechanisms governing assembly morphology, lipid interactions, and homolog-specific organization remain beyond the scope of the present study. Since corresponding multicolor STORM experiments were not performed, we interpret these observations cautiously and discuss them as preliminary. In the updated manuscript, we have now added four new supplementary figures together with corresponding Results and Discussion sections in the revised manuscript.**

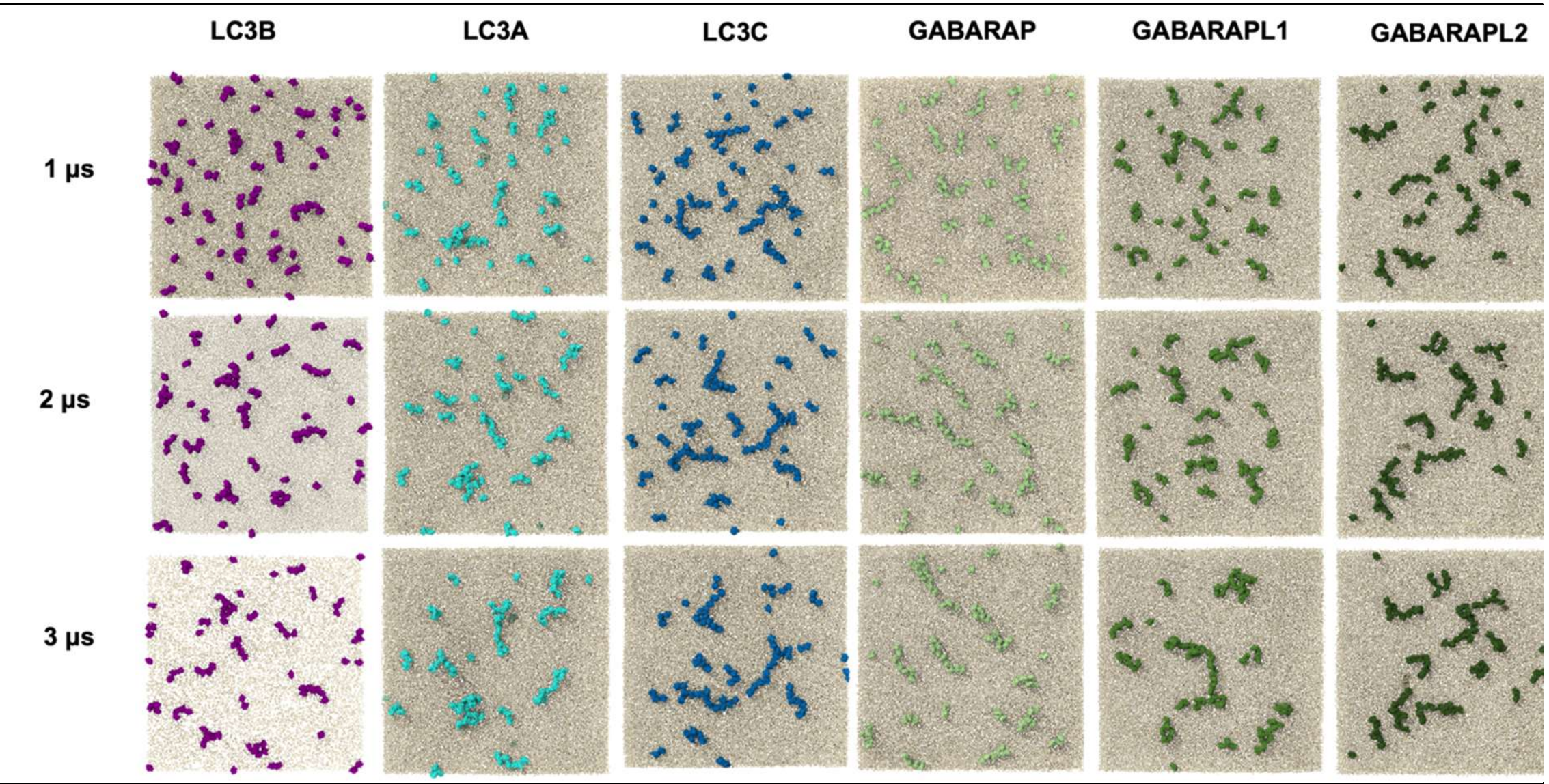


**Figure S12. Clustering behaviour of Atg8 homologs in coarse-grained simulations**. Representative snapshots from coarse-grained molecular dynamics simulations of human Atg8 homologs (LC3A, LC3C, GABARAP, GABARAPL1 and GATE16) at 1, 2, and 3 µs. Across all systems, proteins exhibit a consistent tendency to form clusters qualitatively similar to the behaviour observed for LC3B. These results suggest that clustering is not unique to LC3B but may represent a shared property of Atg8 family members.

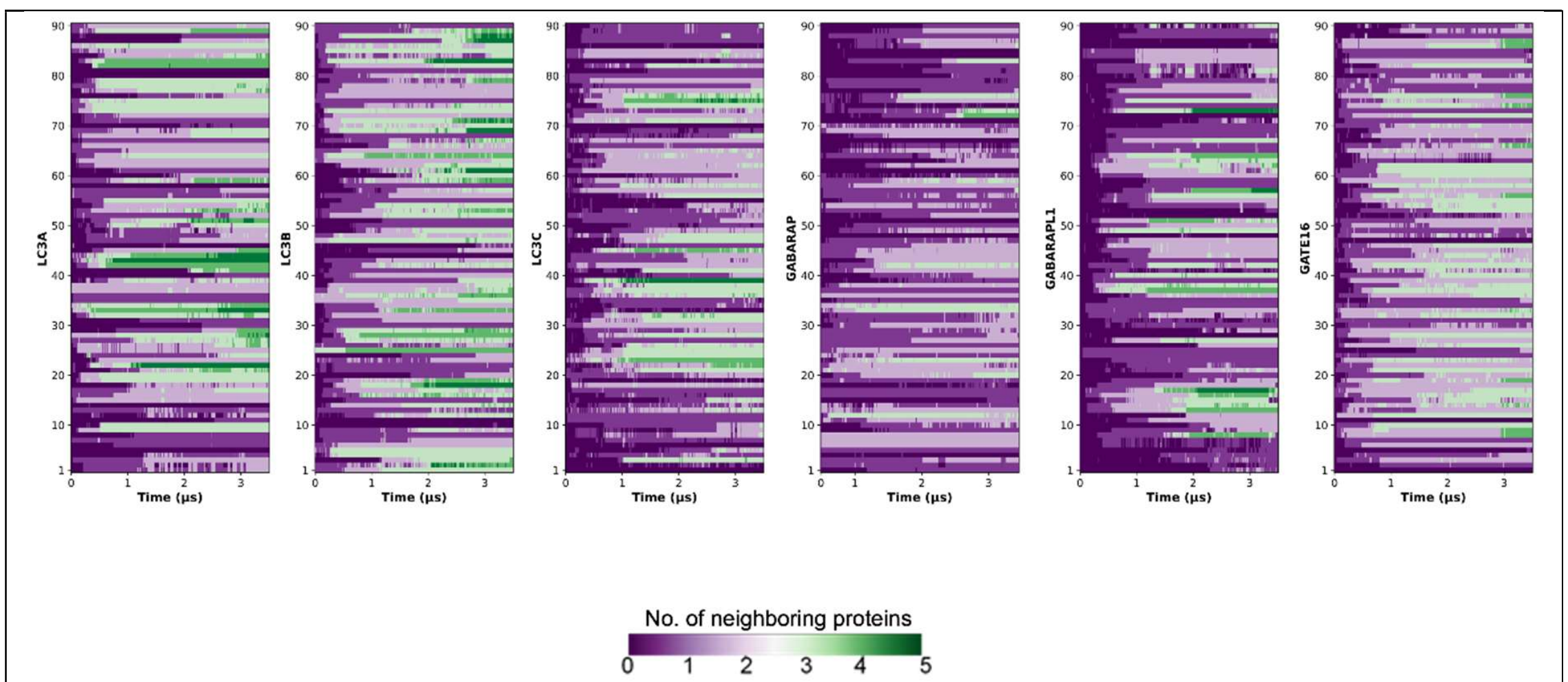


**Figure S13. Comparative clustering behavior of all six ATG8 homologs.** All six homologs (LC3A, LC3B, LC3C, GABARAP, GABARAPL1, and GATE16) exhibit clustering behavior, while differing in their organizational properties. The heatmaps show the number of clustering interfaces present on each protein monomer over time. Neighbor heatmaps suggest that GABARAP tends to form more linear assemblies, characterized by two interfaces per molecule, whereas LC3B exhibits more heterogeneous and distributed contact networks.

Together, these observations preliminarily indicate that although clustering is a shared feature among ATG8 homologs, the underlying interaction topology may differ.

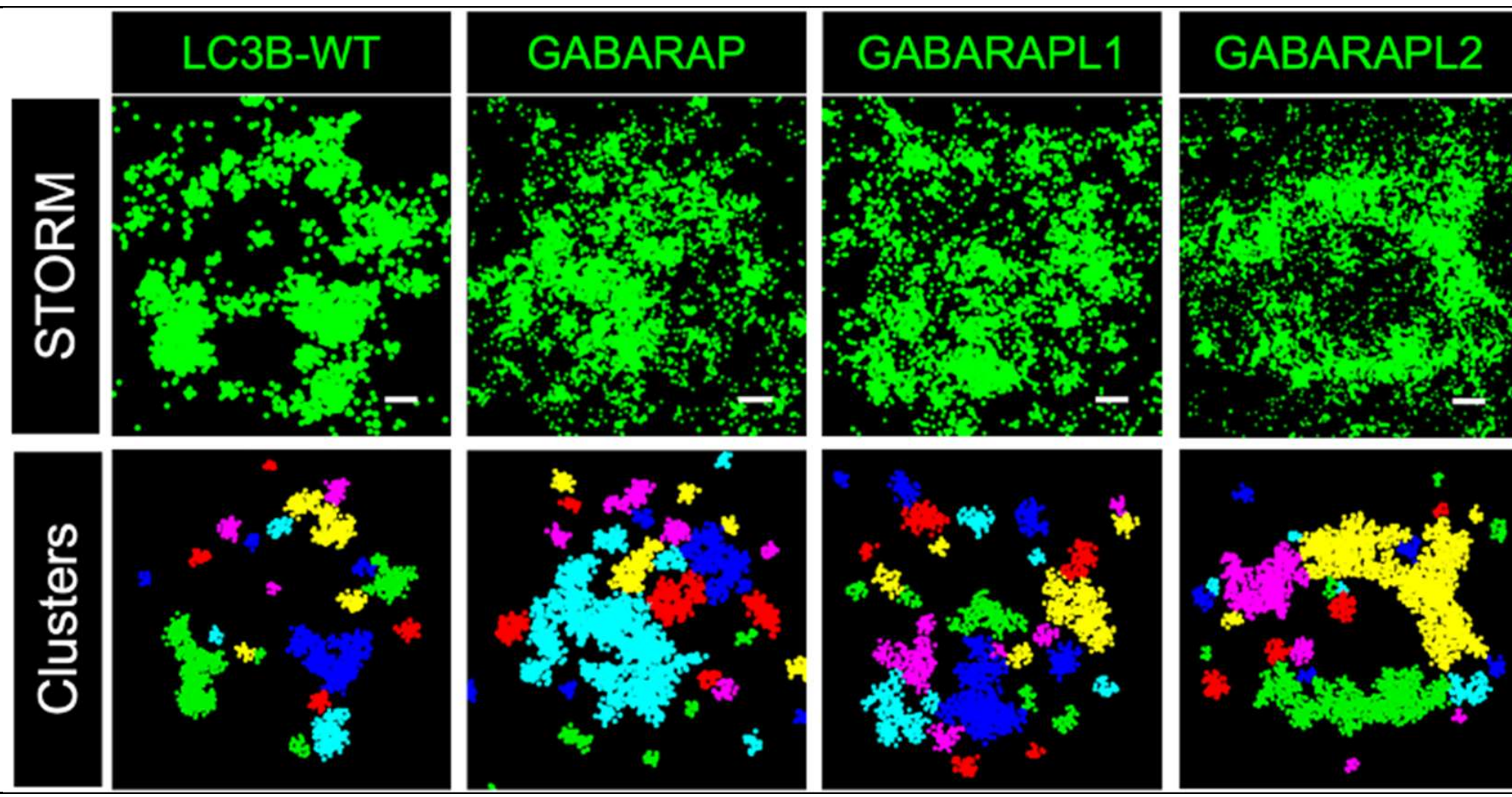


**Figure S14. Representative STORM images of individual autophagosomes from starved BS-C-1 cells transiently expressing LC3B, GABARAP, GABARAPL1, and GABARAPL2 respectively.** Scale bar: 200nm.

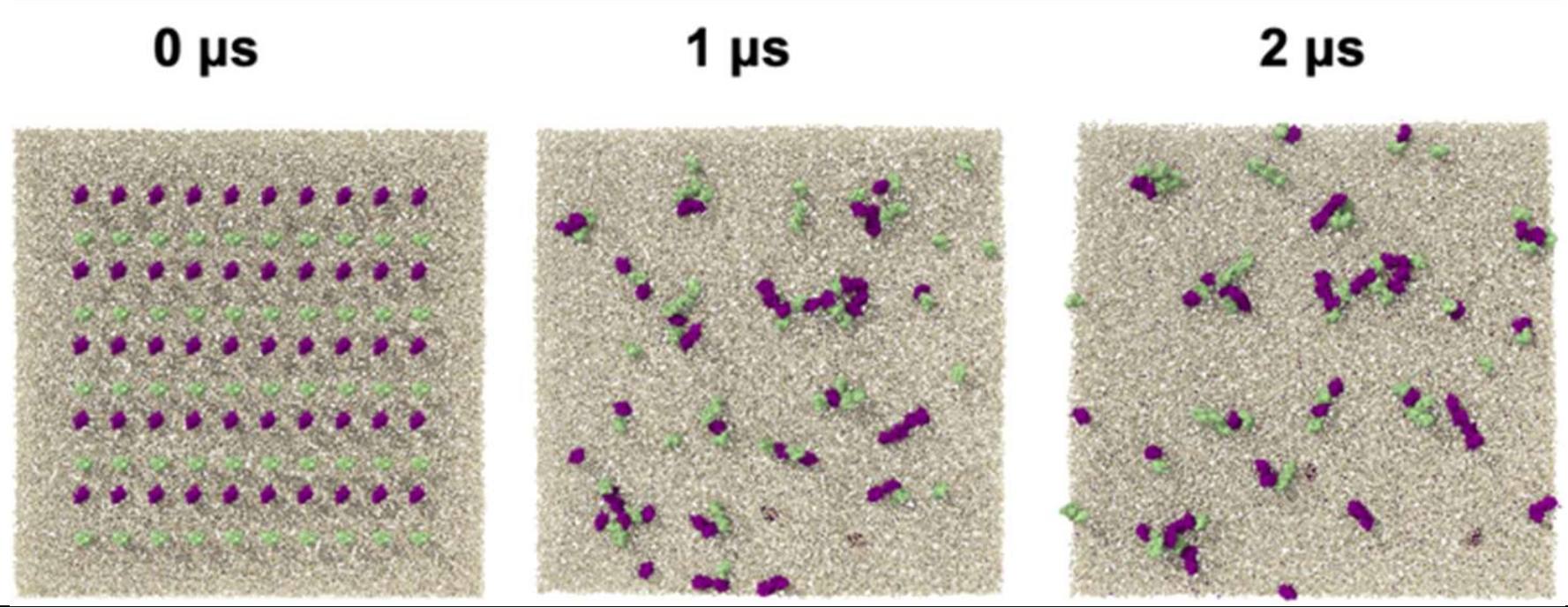


**Figure S15. Hetero clustering of LC3B and GABARAP in mixed simulations.** Representative snapshots from coarse-grained molecular dynamics simulations of a mixed system containing 50 LC3B (purple) and 50 GABARAP (green) molecules at 1.0 and 2.0 µs. The proteins exhibit a tendency to form mixed clusters, with LC3B and GABARAP frequently co-localizing within shared assemblies, suggesting that heterotypic clustering can occur under these simulation conditions.

*R1.2) The authors should provide more than a single simulation for Fig. 4B/C. For consistency with earlier figures, why not use coarse-grained simulations as in Fig. 1? It is not clear what additional insight was gained by switching from coarse-grained to atomistic simulations in this context.*

**Our response: We thank the reviewer for raising this point and will clarify our rationale for using atomistic simulations in Figure 4. While in Figure 1 the primary goal was to investigate large-scale clustering behaviour, in Figure 4 section we aimed at resolving the molecular basis of the dimer–dimer interface and understand how specific mutations perturb these interactions. While CG simulations are highly suitable for studying membrane organization and large-scale protein clustering phenomena, they lack in explicit side-chain representations and therefore cannot adequately capture residue-level packing interactions. We have now clarified this rationale and expanded the description of the simulation strategy in the revised manuscript (Results section Pg 9).**

**Another point raised is with respect to number of replicas. In response, we have now performed additional simulation replicates and included the analysis in the revised manuscript in Figure 4.**

*R1.3) For Fig. S2 (kymographs), quantification is needed, rather than a single example being shown. For example, speed and run length could be quantified.*

**Our response: Following the reviewer's recommendation, we have now included additional experimental replicates and quantitative analyses of the speed and run length from single particle tracking of the autophagosome motility data. In the revised manuscript, corresponding quantifications and statistical analyses are now provided in Supplementary Figure S33 and described in the revised Results section (Pg 11).**

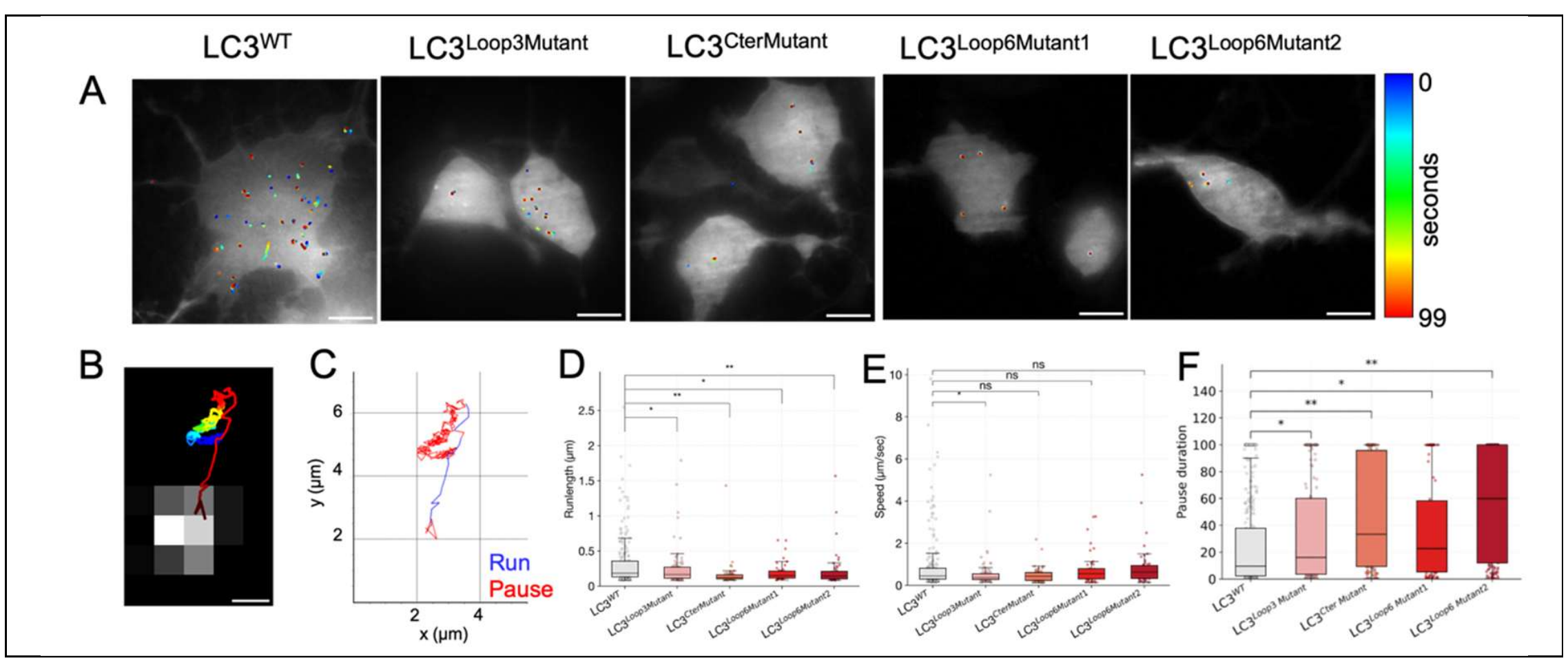

**Figure S33. Single particle tracking analysis reveals altered autophagosome motility across LC3B mutants**. A) Representative single-particle tracking trajectories (colored lines) of **E**GFP–LC3B–positive autophagosomes in Neuro2A cells. Scale bar: 10 µm. Tracks are color-coded by time (T). B) Representative temporal color-coded trajectory of **E**GFP–LC3B$^{WT}$ positive autophagosome. Scale: **2**00nm. C) Representative color-coded **E**GFP–LC3B–positive autophagosome trajectory shows direct run phases in blue and confined pause phases in red. D) Distribution of run lengths for directed trajectories in wild-type and mutant LC3B. E) Distribution of speed of run phases (direct trajectories) for the wild-type and mutant LC3. F) Distribution of pause duration for all trajectories for the wildtype and mutant LC3**.**Data are shown from 18 cells (LC3$^{WT}$), 22 cells (LC3$^{Loop3Mutant}$), 18 cells (LC3$^{CterMutant}$), 23 cells (LC3$^{Loop6Mutant1}$), and 21 cells (LC3$^{Loop6Mutant2}$) pooled from three independent experiments.

*R1.4) It is not entirely clear why the LC3B mutants behave as dominant negatives. While this issue is not disqualifying, it is the main result that does not align with naïve expectations and would benefit from additional discussion or clarification.*

**Our response: We thank the reviewer for raising this important point. Since LC3-mediated self-assembly appears to be important for generating locally concentrated interaction platforms, the incorporation of clustering-defective mutants into these assemblies likely disrupts the spatial organization and collective behavior of endogenous LC3 molecules. Our present understanding is, therefore, that these mutants might interfere with the formation and higher-order organization of LC3 wildtype nanoclusters on autophagosomal membranes. In this context, even partial incorporation of mutant LC3 into nascent nanoclusters could reduce cluster compactness, alter receptor engagement, and impair cargo sequestration efficiency, thereby producing a dominant-negative effect despite the presence of other endogenous Atg8 family members.**

**We agree that the dominant-negative behavior of the LC3B mutants merits discussion for the readers. In the revised manuscript, we have expanded the discussion to better clarify our current interpretation of this phenomenon (Discussion section, Pg 14).**

*R1.5) The authors consider membrane thickness effects (Fig. 1E), but do not address membrane curvature. Do LC3B nanoclusters influence curvature, or does curvature affect cluster formation? The inner and outer membranes of the autophagosome are likely to present distinct physical environments that could differentially impact*

**Our Response: We thank the reviewer for this interesting suggestion and analysis. This is indeed an important question, particularly given the central role of LC3 in autophagosome biogenesis and membrane organization. As recommended, we computed membrane curvature-related parameters across all three simulation replicas.**

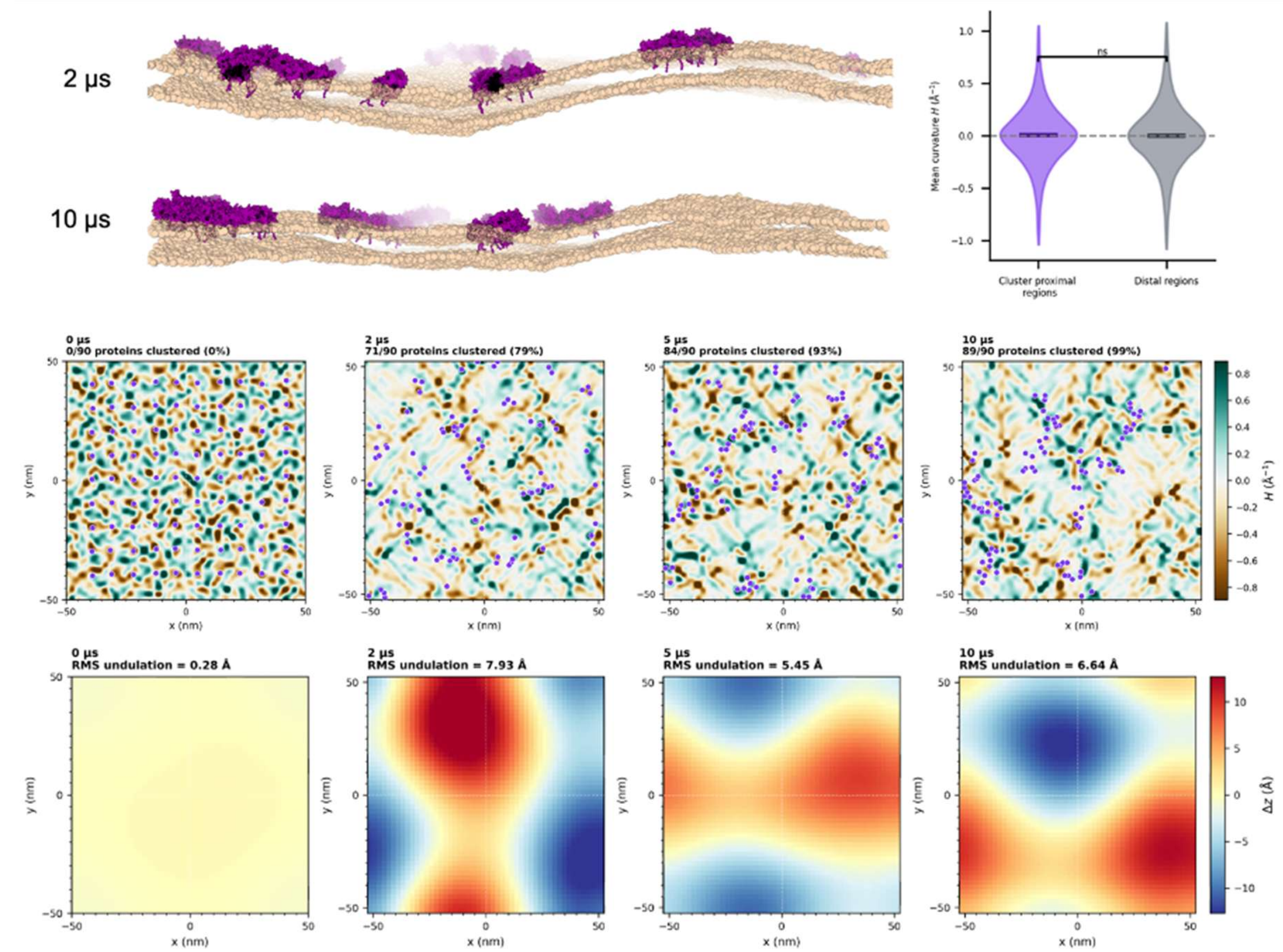


**Figure S3: LC3B clustering is curvature-independent locally, but membrane shows global undulations.** Top: Representative coarse-grained snapshots showing LC3B (magenta) on the membrane illustrating subtle membrane curvature. Top right: Distribution of local mean curvature (H) in cluster-proximal and distal regions shows no significant difference (cluster: 0.00185 ± 0.32 $Å^{-1}$; non-cluster: −0.00329 ± 0.32 $Å^{-1}$; Mann–Whitney p = 0.3065, n.s.), indicating absence of curvature enrichment at cluster sites. Middle row: Spatial maps of mean curvature (H) at different time points with LC3B centers of mass overlaid (purple).

**Overall, the analyses presented in Supplementary Figure S3 suggest that LC3 self-assembly and membrane undulations emerge concurrently but at distinct spatial scales. As simulations progress, collective accumulation of LC3 assemblies is accompanied by broader membrane undulations across the bilayer surface. However, LC3 clustering itself is not restricted to pre-curved membrane regions and instead forms robustly throughout the membrane through local oligomeric interactions. Consistently, the curvature distributions for cluster-proximal and distal membrane regions were highly similar, indicating that LC3 nanoclusters do not preferentially partition into regions of distinct local curvature. Together, these observations support a multiscale organizational model in which nanoscale LC3 assemblies arise locally through transient protein–protein interactions, while larger membrane undulations emerge as a collective mesoscale property of increasing membrane-associated LC3 organization.**

**We also agree that the inner and outer autophagosomal membranes may present distinct physical environments. However, both our simulations and current STORM imaging do not**

**resolve these membranes separately, as the intermembrane spacing (~10–30 nm) lies below the effective resolution of our approach. Thus, our analysis primarily reflects LC3 organization at the accessible membrane surface and resolving potential differences between inner and outer membrane-associated LC3 remains an important direction for future work.**

*Minor points from Reviewer #1*

*The heterogeneity among interface contacts is reminiscent of ubiquitin-ubiquitin interactions (PMID: 37225996) and could be discussed.*

**Our response: Thanks for this interesting observation. The heterogeneity within distinct regions observed in LC3B dimers indeed has parallels with the ubiquitin–ubiquitin interactions reported to drive FAM134B receptor nanoclustering during ER-phagy (González et al., 2023). In this study, trans-ubiquitin contacts were distributed across the entire protein surface without a dominant interface yet collectively enabled functional membrane remodelling. We have now included the discussion around this theme in the revised manuscript on (Pg 13).**

**LC3B appears to occupy an intermediate position along this specificity spectrum: although Loop6 acts as a primary interaction hub, the four binding modes we identify featuring contributions from α1–α2, Loop6–α4, and C-terminal regions highlight a degree of interface degeneracy.**

*Line 218 / Fig. S14: It is not clear where the SMVSVS motif maps onto the sequence logo.*

**Our response: We have updated the Figure S22 (previously Figure S14) to mark the SMVSVS motif in black box.**

*Line 265: "Our positive control is wild-type LC3-eGFP..." should be corrected to "eGFP-LC3."*

**Our response: We apologise for this typo. The same has now been corrected in the manuscript.**

*Line 402: WIPI proteins do not contain FYVE domains; this should be corrected.*
**Our response: We apologise for this typo. The same has been now corrected in the manuscript.**

**Referee #2:**

*Fatihi et al. report that LC3B forms nanoclusters that are important for autophagy. The*

*strength of the paper is in the MD simulations and in the general concept, which makes sense. I have a great many questions about the experimental details and interpretations.*

**Our response: We thank the reviewer for the careful evaluation of our manuscript and for the constructive questions regarding the experimental analysis. We address each comment in detail below:**

*R2.1) LC3B density: If LC3B is spaced 8 nm apart, that works out to 64 nm2 per LC3, or 1000 LC3D per island. How does this fit with other numbers in the literature?*

**Our Response: We thank the reviewer for this important observation regarding the effective LC3 surface density in the simulations. As correctly estimated by the reviewer, an average intermolecular spacing of ~8 nm corresponds to an approximate projected membrane area occupancy of ~64 nm² per LC3 molecule. For the simulated membrane patch (~100 × 100 nm²), this yields an initial configuration containing ~90 LC3 molecules, corresponding to a local surface density of ~9 × $10^{-3}$ molecules/nm². Importantly, however, the initial spacing was not intended to represent a quantitatively fixed physiological stoichiometry of LC3 molecules on autophagosomal membranes. Rather, it was selected as a computationally tractable starting condition that permits sufficient lateral diffusion, membrane exploration, and spontaneous self-assembly within accessible coarse-grained simulation timescales. At substantially lower initial densities, spontaneous cluster formation was not observed within the accessible simulation window due to diffusion-limited encounter frequencies. Thus, the chosen density represents an effective mesoscale sampling regime designed to probe whether weak membrane-associated LC3–LC3 interactions are intrinsically sufficient to drive local oligomerization.**

**Geng et al. estimated approximately 272 Atg8 molecules at the phagophore assembly site in yeast using calibrated fluorescence microscopy (J Cell Biol, 2008). Although this measurement does not directly provide a surface density, it indicates that hundreds of Atg8 molecules can accumulate within individual autophagic structures. Given the uncertainty in the size, geometry, and local organization of these assemblies, it remains difficult to directly translate such measurements into intermolecular spacings for comparison with simulations.**

**We also note that experimentally measured LC3 surface densities on autophagosomes remain difficult to estimate precisely and are expected to vary dynamically during phagophore expansion and maturation. Our STORM analyses further indicate that LC3 assemblies exhibit a broad size distribution, suggesting that local enrichment of LC3 can occur heterogeneously on membranes. Therefore, the simulations were not intended to quantitatively reconstruct absolute physiological LC3 copy numbers, but rather to capture the underlying physical principles governing membrane-associated LC3 self-organization. We have now clarified this rationale in the revised manuscript Discussion (Pg 14).**

*R2.2) LC3 self association. The authors state there are 43 solved structures showing an empty pocket. Many of these are crystallographic? The authors should check if any of them are occupied in self-associating lattice contacts. If not, why not?*

**Our response: We thank the reviewer for this insightful observation. To address this point, we systematically analyzed crystal packing contacts across 69 unique PDB structures representing all six human Atg8-family homologs together with yeast Atg8. For each structure, we identified residues involved in intermolecular crystal contacts and quantified their frequency across the dataset. Interestingly, residues encompassing Loop 6 (L6), helix α4, and the C-terminal region (residues ~84–101 and 115–120) emerge as the most recurrently engaged surface in crystal packing interactions. Importantly, when structures are analyzed separately by homolog, the same enrichment at the L6–α4–CT surface is consistently observed across LC3A, LC3B, LC3C, GABARAP, GABARAPL1, GABARAPL2, and yeast Atg8, indicating that this feature is conserved throughout the Atg8 family rather than arising from a particular crystal form or homolog.**

**Because this interaction hotspot was consistently observed across all human ATG8 homologs and yeast Atg8, we strongly suggest that this is an intrinsic structural propensity for weak intermolecular association. Together with the simulation and imaging analyses, these observations support a model in which the L6-associated surface contributes to membrane-associated nanoscale LC3 organization. These new results are described in Results section (Pg 9) and a new supplementary Figure S25 has been added.**

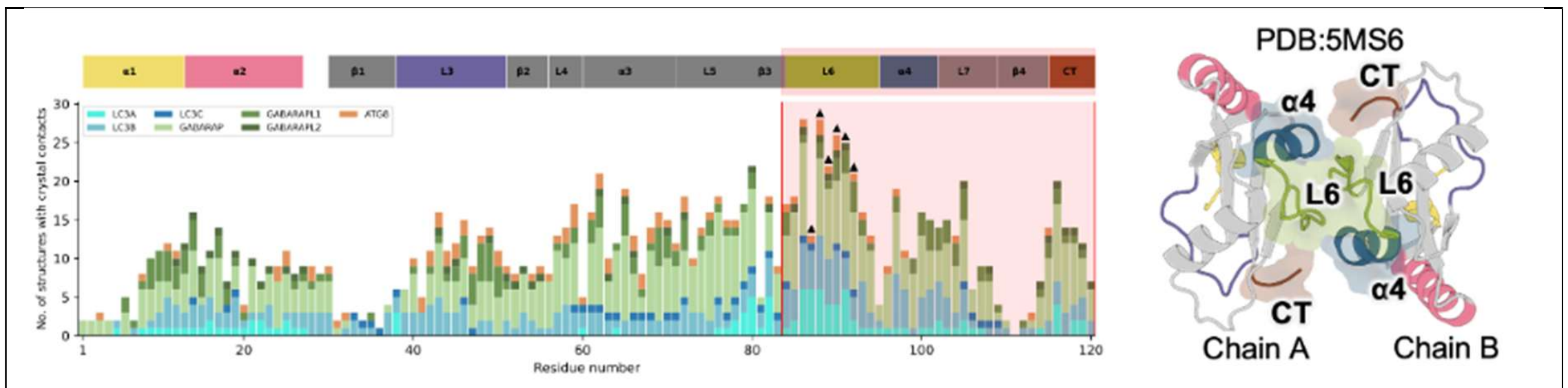


**Figure S25: Crystal packing reveals a recurrent LC3 self-association interface.** Percentage of crystal structures in which each residue participates in lattice contacts across 69 Atg8-family structures. Residues within Loop6 and helix α4 show the highest contribution to crystal packing. Secondary structure elements are annotated above the plot. The shaded region highlights the L6–α4–C-terminal (CT) interface (residues ~84–101 and 115–120). The black triangle marks the SMVSVS motif. Representative crystal structure PDB: 5MS6 illustrating Loop6 positioned at the dimer interface, consistent with its recurrent engagement in lattice contacts across structures (right).

*R2.3) LC3B is overexpressed in these studies on top of wild-type LC3B. The implications of a potential dominant negative mechanism should be discussed. Transfection: show if there is*

*equal expression and compare to endogenous levels. Are there changes in LC3A/B, GABARAPs?*

Our response: **We thank the reviewer for raising this important point regarding potential dominant-negative effects arising from overexpression of LC3B mutants in the presence of endogenous LC3 proteins. To address this, we examined the expression levels of endogenous LC3B and GABARAP following transfection with the different GFP-LC3 variants (Figure below). As shown, endogenous LC3B and GABARAP levels remain largely unchanged across all mutant conditions, indicating that expression of the GFP-LC3 variants does not substantially perturb the steady-state levels of endogenous Atg8 family proteins. However, marginal reduction in endogenous LC3 levels was observed in the Loop3 mutant condition. We additionally confirmed comparable expression of the transfected GFP-LC3 constructs across conditions. These observations support the interpretation that the observed phenotypes are not driven by major alterations in endogenous LC3/GABARAP abundance.**

**However, we also agree with the reviewer that dominant negative effect via over-expression is possible. In the revised manuscript, we have discussed the same on Pg 1, and results section has also been updated with additional experiment described on Pg 10. A new Supplementary Figure is added as Figure S30. In particular, we have now expanded the Discussion section to address this point and to clarify how clustering-defective mutants may influence higher-order LC3 assembly behavior in the presence of endogenous LC3 proteins.**

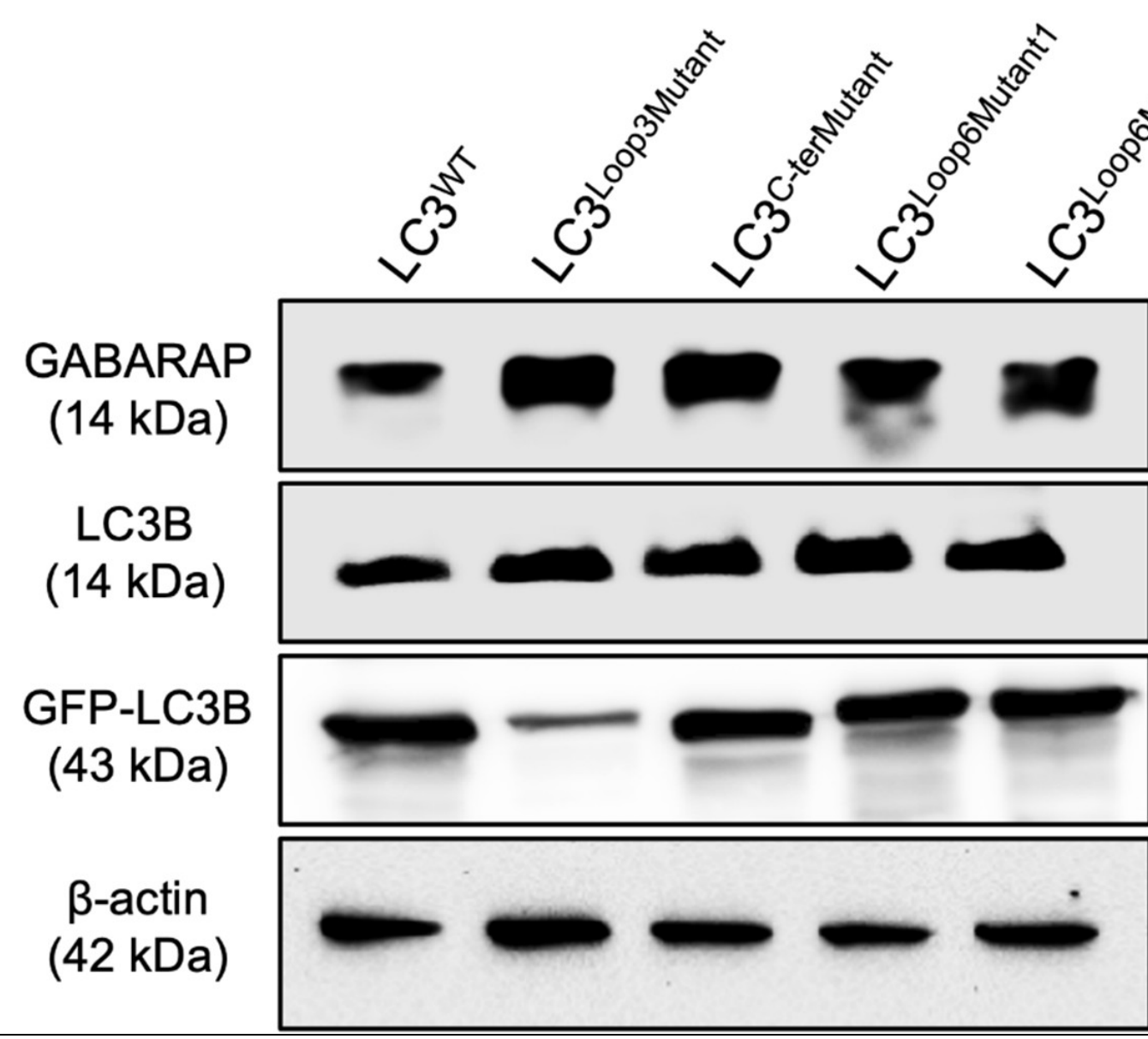


**Figure S30:** Immunoblot depicting the endogenous GABARAP and LC3B levels in host cells following transfection of different GFP-LC3 variants. GABARAP and LC3 levels remained unchanged across all mutant variants.

*R2.4) STORM visualizes clusters of 1000 or more LC3B on a scale of 150-200 nm, which is far more massive than the clusters simulated in the MD. Yet the term cluster is used as if these are the same thing. They are actually completely different. The authors should use clear terminology to separate these things. Then they need to explain how the two different scales are related to each other.*

**Our response: We have explained this above as was also pointed by the editor as a critical point. We would like to emphasise that we agree with this change and have made modificaitons throughout the manuscript.**

**To clarify that the large LC3 assemblies referred to by the reviewer do not represent the predominant population observed in our STORM data. Moreover, our data do not directly support the existence of discrete clusters containing ~1000 LC3 molecules, and we apologize if the original presentation led to this interpretation. While STORM allows us to quantify the spatial dimensions of LC3-positive assemblies, determining the exact number of LC3 molecules within a given structure remains challenging because of uncertainties associated with fluorophore blinking, labeling efficiency, and molecular packing density.**

**Our Gaussian distribution analysis of STORM-resolved LC3 assemblies (Figure 2F) shows that the majority of LC3-positive structures are centered around ~150–300 nm. We therefore consider these assemblies to represent the dominant organizational state of LC3 on autophagosomal membranes. Larger assemblies are observed only infrequently, with a small fraction of structures extending to sizes approaching ~1500 nm. We interpret these rare, larger assemblies as higher-order organizational states that may arise on a subset of unusually large or mature autophagosomes rather than representing the typical LC3 architecture within cells.**

**We apologize for the lack of clarity in the original presentation and have revised the Results section, Discussion, and figure legends (Pg 14) to more clearly distinguish between the nanoscale LC3 oligomers observed in simulations and the larger supramolecular LC3 assemblies detected by STORM imaging, and to explain how these organizational states are related across scales.**

*R2.5) This paper reminds me of one by Rod MacKinnon in PNAS, Nov. 2025, on higher order transient structures. (HOTS). The MacKinnon paper is much deeper. I don't expect the authors to do everything MacKinnon did, but do please cite the paper and compare the ideas. This paper is a good example of clarity in discussing nanoclusters of a few molecules as opposed to larger mesoscale entities.*

**Our response: We thank the reviewer for highlighting this very relevant and insightful study by Zhang and MacKinnon (PNAS 2025) on Higher-Order Transient Structures (HOTS). In our**

**study, this was indeed a central focus i.e., to identify weak LC3–LC3 interaction interfaces underlying membrane-associated nanoscale organization and to assign possible structural determinants governing these transient assemblies. At the same time, we agree with the reviewer, the distinction between smaller nanoscale LC3 assemblies captured in simulations and the larger supramolecular membrane-associated assemblies observed by STORM imaging was not sufficiently articulated in the original manuscript. We have now clarified and discussed these multiscale organizational features in the revised Discussion section.**

**However, there are also important differences in both biological context and scale. In our study, the coarse-grained MD simulations primarily capture the formation of small nanoscale LC3 assemblies driven by local protein–protein interactions on membranes, whereas STORM imaging additionally reveals larger supramolecular LC3 assemblies at the autophagosomal surface. Following the reviewer's suggestion, we have now clarified the terminology throughout the manuscript to distinguish small "nanoclusters" from larger "supramolecular assemblies" observed experimentally.**

**We have also expanded the Discussion section to better relate our findings to the HOTS and dynamic connectivity framework proposed by MacKinnon and colleagues, while emphasizing the distinct membrane-remodeling and autophagy-specific context of LC3 organization.**

*R2.6) Check grammar throughout, as there are frequent missing articles and wrong tenses. Font sizes in figures are almost unreadably small in some cases (mutant labels in Fig. 5, for ex.).*

**Our response: We apologise for grammar, but we have used AI now for grammar correction.**

*R2.7) In Fig. 5 the phenotypes vary in ways that are hard to understand. Loop3 and Cter have no difference in number of clusters/autophagosome and less area/LC3B, while Loop6 mut 1 and 2 have fewer clusters/autophagosome and more area/LC3B. Why are the effects different on mesoscale clustering? Since the effects are so different, why are the effects on function the same? How does breaking the L6 interface lead to the single blob like clusters described? The rationalization offered is that there are also multivalent interactions with lipids. What if all the effects are actually mediated by changes in lipid co-clustering?*

**Our response: We appreciate reviewer's perspective regarding mutant phenotypes and will take this opportunity to explain further.**

**Firstly, $LC3B^{Loop3}$ and $LC3B^{C\text{-}terminalMutant}$, which exhibited smaller membrane-associated assemblies compared to $LC3B^{WT}$, were designed to probe the contribution of peripheral interface regions to higher-order LC3 assembly. Cluster analysis confirmed that Loop 6 is the**

**dominant interaction hub, exhibiting a 2.5–4 times higher mean inter-protein contact frequency (41.4%) compared to Loop 3 (~10.7%) and C-terminal (~19.4%) regions (Figure 3B). Consistent with their peripheral roles, $LC3^{Loop3Mutant}$ and $LC3^{CterMutant}$ mutants retained partial clustering capacity, producing assemblies that were smaller but not abolished, resulting in only a modest change in the overall clustering pattern. In contrast, the Loop 6 mutants retained membrane tethering ability but largely abolished protein–protein interactions, confirming that Loop 6 is the primary driver of LC3 self-association. Consequently, $LC3^{Loop6Mutants}$ proteins failed to form higher-order assemblies and instead displayed a significantly expanded, diffuse membrane footprint compared to $LC3B^{WT}$, consistent with impaired clustering on the membrane.**

**Secondly, we agree with the reviewer that the mesoscale phenotypes are not identical and, importantly, neither are all functional readouts. While autophagosome motility assays show broadly similar defects across the $LC3^{Loop3Mutant,}$ $LC3^{CterMutant}$ and $LC3^{Loop6Mutants}$ other functional measurements reveal clear differences in the severity of impairment. For example, in the bacterial degradation assay, the $LC3^{CterMutant}$ retain approximately 15% SPN colocalization compared to ~30% for $LC3B^{WT}$, whereas the Loop 6 mutants show only ~5–6% colocalization. Thus, the functional consequences scale with the degree of disruption of the assembly network, with L6 mutations producing a substantially stronger defect than C-terminal mutations. We speculate that autophagosome motility may require a relatively precise supramolecular organization of LC3 assemblies, such that disruption of either interface is sufficient to compromise this process and produce a similar phenotypic outcome. In contrast, cargo degradation appears more sensitive to the overall integrity of the LC3 interaction network, allowing differences between partially disrupted (C-terminal) and severely disrupted (Loop 6) assemblies to become apparent. We have revised the text to clarify that the mutants do not exhibit identical functional phenotypes and that different autophagy-associated processes may have distinct structural requirements for LC3 organization.**

**Thirdly, we thank the reviewer for raising the possibility that the observed phenotypes arise from altered lipid co-clustering. To address the co-clustering effect, we performed sequential two-colour STORM imaging of PI(3)P and $LC3B^{Loop6Mutant2}$. In contrast to LC3B-WT, where LC3 clusters show substantial overlap with PI(3)P-enriched nanodomains marked by the FYVE probe, the spatial relationship between $LC3B^{Loop6Mutant2}$ and PI(3)P appears markedly altered. Most $LC3B^{Loop6Mutant2}$ assemblies do not colocalize with PI(3)P-enriched lipid clusters, and the extent of overlap is substantially reduced compared to WT. These observations suggest that LC3 mesoscale assembly and PI(3)P organization are closely coupled. While the data support a role for protein–lipid co-clustering in organizing autophagosomal membranes, the precise mechanistic relationship between LC3 self-association and PI(3)P nanodomain formation remains unclear and will require further**

**investigation. In the revised manuscript, we have updated the new results as Supplementary figure (Figure S31) and updated Results are available on Pg 10. An extensive discussion is also included in the revised manuscript on Pg 15.**

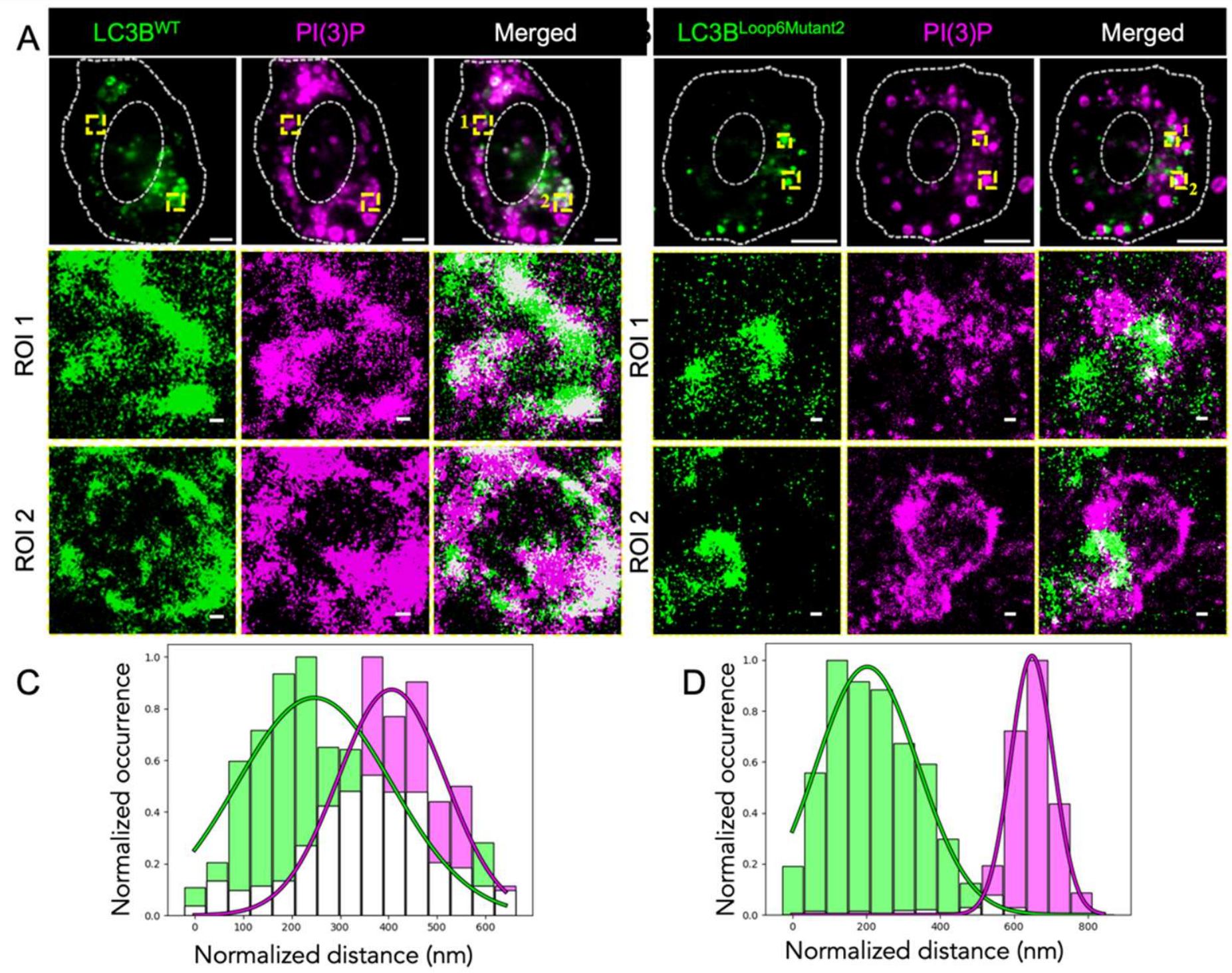


**Figure S31: Super-resolution imaging reveals diminished association of the LC3B Loop 6 mutant with PI3P-positive vesicles**. Sequential two-colour 2D STORM imaging of starved BS-C-1 cells overexpressing the mCherry-tagged 2×FYVE domain of HRS together with either EGFP-LC3B$^{WT}$ or EGFP-LC3B$^{Loop6Mutant2}$, followed by immunostaining against EGFP and mCherry. (A,B) Representative STORM images of cells expressing EGFP-LC3B$^{WT}$ (A) or EGFP-LC3B$^{Loop6Mutant2}$ (B). Insets highlight representative regions showing the overlap between autophagosomes and PI3P-positive vesicles. (C,D) Representative localization histograms depicting the overlap between 2×FYVE-HRS and EGFP-LC3B$^{WT}$ (C) or EGFP-LC3B$^{Loop6Mutant2}$ (D). Scale bars: 5 µm; inset scale bars: 200 nm.

*R2.8) All mutants have less track displacement and less FYCO1 colocalization. FYCO1 binding- the authors should confirm FYCO1 LIR binding to LC3 is unaffected using purified proteins.*

**Our Response: We thank the reviewer for this important suggestion. To address this, we performed GFP pull-down assays to examine FYCO1 association with the different LC3 mutants (Figure below). As observed, the mutants did not abolish FYCO1 binding albeit subtle differences in association were detected. These results suggest that the reduced motility phenotype is unlikely to arise primarily from loss of FYCO1–LC3 binding affinity. At the molecular level, this observation is consistent with our structural analyses (Figure 3F), which show that the LIR-binding pocket is structurally distinct from the clustering interface. Thus, the clustering-interface mutants predominantly affect higher-order LC3 organization and membrane-associated assembly properties rather than directly perturbing LIR-mediated FYCO1 recognition.**

**In modified manuscript, we have now updated the Results section (Pg 11) and new supplementary figure (S34) to include these critical results.**

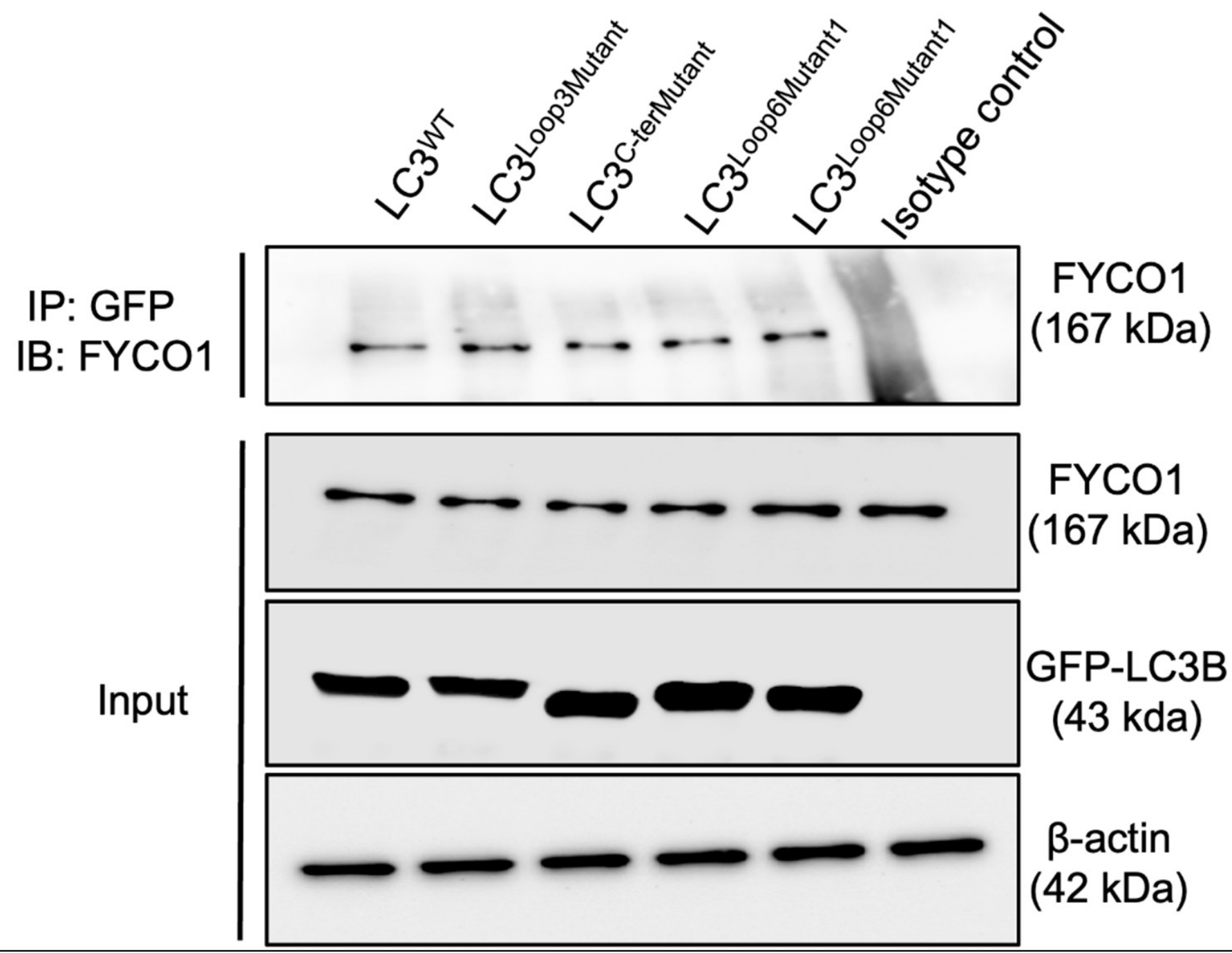


**Figure S34: Immunoblot demonstrating the interaction of FYCO1 with different variant of GFP-LC3.** Host cells were transfected with GFP-LC3 variants for 36 h and lysates obtained were immuno-precipitated with anti-GFP Ab, followed by immunoblotting with anti-FYCO1 Ab.

*R2.9) Fig. S20 show large unexplained differences in conjugation efficiency. Methods for Fig. S20 are not explained. Was autophagy induced, to drive LC3-II formation? L6 M1 has lower conjugation but other mutants have higher. The differences don't correlate with the phenotypes but are in some cases large, especially for the C term mutant (which is not surprising given its location). More thought should be given to the possiblility that the phenotypes relate to conjugation efficiency instead of nanoclustering. The authors should show conjugation is equal under the conditions of the experiments in Fig 5 and Fig 6.*

**Our Response: We thank the reviewer for pointing this out. To clarify, Fig. S20 (now Figure S29) was included primarily to confirm that all LC3 mutants retain the ability to undergo lipidation and generate the LC3-II species. The experiments were performed under the same cellular conditions used throughout the study. We agree with the reviewer that modest differences in LC3-I/LC3-II partitioning are observed among some mutants.**

**To further address this point, we had additionally examined interactions of the mutants with core autophagy conjugation machinery components, including ATG3, ATG4B, and ATG7 (previous Fig. S23 now Figure S37). As shown, the mutants largely retain interactions with the autophagic machinery (with the exception of the C-terminal mutant), supporting the interpretation that the observed phenotypes primarily arise from altered LC3 organization. Notably, the C-terminal mutant behaves differently from the other mutants, which is not unexpected given that this region lies in close proximity to the lipidation and conjugation interface (as also pointed by the reviewer). Accordingly, this mutant shows comparatively altered interaction patterns with the conjugation machinery and was therefore interpreted separately from the clustering-interface mutants in our analyses. In contrast, the L3 and L6 mutants retain interactions with the conjugation machinery while still exhibiting altered clustering and motility phenotypes, supporting the conclusion that these effects are linked more directly to LC3 organization and nanoscale assembly behaviour rather than a major defect in conjugation efficiency. We have now expanded the results section to clarify these observations (Pg 12).**

*R2.10) Analysis of movies 10-14- there are many static and dynamic objects. It was not clear to me how some of the static objects in the mutant analyses were chosen for analysis. The apparent mutational loss of function could be an artifact of biased selection of objects for analysis, if I understand what the authors did.*

**Our response: We thank the reviewer for pointing this out. To clarify, the Movies 10–14 show representative live-cell TIRF movies of autophagosomes in Neuro-2A cells. The arrows highlight examples of highly mobile autophagosomes observed in wild-type cells, but not in mutant-expressing cells, and do not represent objects selected for tracking analysis. All the detectable autophagosomes from cells were selected for kyomograph analysis. The corresponding kymographs are presented in Figure 32. Briefly, kymographs provide a two-dimensional representation of spatial position as a function of time and serve as an unbiased qualitative visualization of autophagosome motility without requiring manual particle selection. In these plots, diagonal traces correspond to mobile autophagosomes, whereas vertical traces correspond to stationary autophagosomes.**

**However, following the suggestion from Reviewer 1 (Comment R1.3), we performed a quantitative single-particle tracking analysis of autophagosomes in Neuro-2A cells. Single-particle trajectories of autophagosomes were obtained using a custom-developed, semi-automated particle-tracking software, followed by quantification of motility parameters using a custom-written MATLAB script as described previously (**Mohan, et al. ***Journal of Cell Biology*** **218.2 (2019): 632-643). The details of the tracking procedure have been included in the Methods section (Pg 21) of the revised manuscript. Using this approach, we quantified autophagosome motility parameters, including the speed, run length and pause duration of the mobile population, and present these data in Figure S33. We have also revised the**

**Results section on Pg 11 of manuscript to clearly describe the updated findings.**

*R2.11) It is very interesting that PI3P and LC3B clusters coincide. This seems to be one of the stronger conclusions of the paper but the implications are not as analyzed and discussed as thoroughly as they could be.*

**Our response: We thank the reviewer for highlighting the importance of lipid interactions and agree that we had previously understated this aspect of the study. In response, we have substantially expanded our analysis and added as a separate section titled "LC3B clusters preferentially associate with PI(3)P lipids".**

**To directly investigate the relationship between LC3 clustering and PI3P-rich membrane domains, we additionally performed a new set of coarse-grained molecular dynamics simulations incorporating PI3P directly into the membrane composition (directly compares with experimental lipid type). Specifically, we simulated multiple membrane-bound LC3B molecules on a heterogeneous lipid bilayer containing 60% DOPC, 20% DOPE, 5% DOPS, 10% POPI, and 5% PI3P. Over 10 μs of simulation, PI3P lipids accumulated preferentially around LC3 assemblies and formed locally enriched membrane domains beneath LC3 clusters. Notably, PI3P exhibited spatial enrichment patterns similar to those observed for PI lipids, consistent with the shared inositol headgroup chemistry of these phosphoinositides. These simulations independently support our super-resolution imaging observations and reinforce the conclusion that phosphoinositide lipids, including PI3P, contribute to the organization of LC3 assemblies on autophagosomal membranes. We have incorporated these new results into the Results and Discussion sections and added a new supplementary figure (Figure S17) presenting the PI(3)P simulation analyses.**

*R2.12)* line 403. Wrong. WIPI proteins do not contain FYVE "motifs".
**Our response: We apologise to the reviewer. The typo has now been corrected.**

*R2.13) In the end, I am not clear about what the nanoclusters are actually doing. The discussion should be more forthright and specific about limitations. For ex, discussion, line 414, "For instance, in the selective autophagy of specific 414 organelles or protein aggregates, it may guide the autophagic machinery to the exact location 415 where it is needed." What does "it" refer to? I assume this refers to the nanocluster. How do the authors imagine that a nanocluster can guide the autophagic machinery to the exact location where it is needed, in a way that individual or randomly distributed molecules could not?*

**Our response: We thank the reviewer for this important conceptual point. Let us clarify that we do not propose that nanoclusters simply "guide" autophagy machinery spatially. Rather, our interpretation is that nanoscale LC3 assemblies may generate locally enriched**

**membrane reaction hubs that enhance multivalent interactions with cargo receptors, adaptors, and membrane remodelling proteins. Compared to randomly distributed individual molecules, such higher-order organization could increase local avidity, stabilize cargo-associated membrane domains, and facilitate coordinated recruitment of downstream autophagy machinery during selective autophagy. We also explicitly acknowledge that the precise mechanistic consequences of these assemblies remain to be established experimentally.**

**For instance, during selective autophagy of specific organelles or protein aggregates, such nanoscale organization may help spatially concentrate and coordinate the autophagic machinery precisely at sites requiring targeted degradation. We agree that the earlier wording was insufficiently precise regarding the potential functional role of LC3 nanoclusters. In the revised Discussion, we now clarify that as major discussion Point on Pg 16.**

Referee #3:

*In this study, the authors highlight the functional importance of LC3B nanoclustering in autophagy. Combining molecular dynamics simulations with super-resolution microscopy, they report that LC3B self-assembles into ~150 nm "islands" on autophagosome membranes with a preference for PI3P. They further identify a hydrophobic-polar clustering motif; mutations in this region disrupt LC3B clustering, weaken FYCO1 association, and, upon loss of LC3B nanoclustering, significantly compromise autophagic entrapment of Streptococcus pneumoniae, resulting in elevated intracellular bacterial survival. This is an interesting study for a broad audience; however, the manuscript needs a few clarifications to support its claims, particularly regarding the role of LC3B in the late maturation stage.*

**Our response: We thank the reviewer for the thoughtful evaluation of our study and for the constructive and insightful comments. We have carefully addressed each point raised and provide detailed responses below.**

R3.1*) The manuscript states that LC3 nanoclusters appear as early as WIPI2-positive phagophores and persist through LAMP2-positive autolysosomes, implying that clusters accompany autophagosomes throughout maturation. This claim should be supported by quantitative co-localization (and ideally temporal analysis) with additional autophagy markers across stages (e.g., initiation/elongation, closure, fusion), not limited to WIPI2 and*

*LAMP2.*

**Our response: As suggested by the reviewer, we have expanded our analysis to include quantitative colocalization of LC3 with additional autophagy markers representing distinct stages of autophagosome biogenesis and maturation, including ATG13 (initiation) and ATG16L1 (elongation). We performed dual-color TIRF imaging which revealed substantial colocalization of LC3-positive vesicles with all four markers (previously including WIPI2 and LAMP2). Importantly, corresponding dual-color STORM imaging demonstrated that LC3 forms nanoscale membrane-associated clusters through all temporal stages of autophagy. The new results are shown in figure below and are now added in the manuscript as Supplementary Figure S11.**

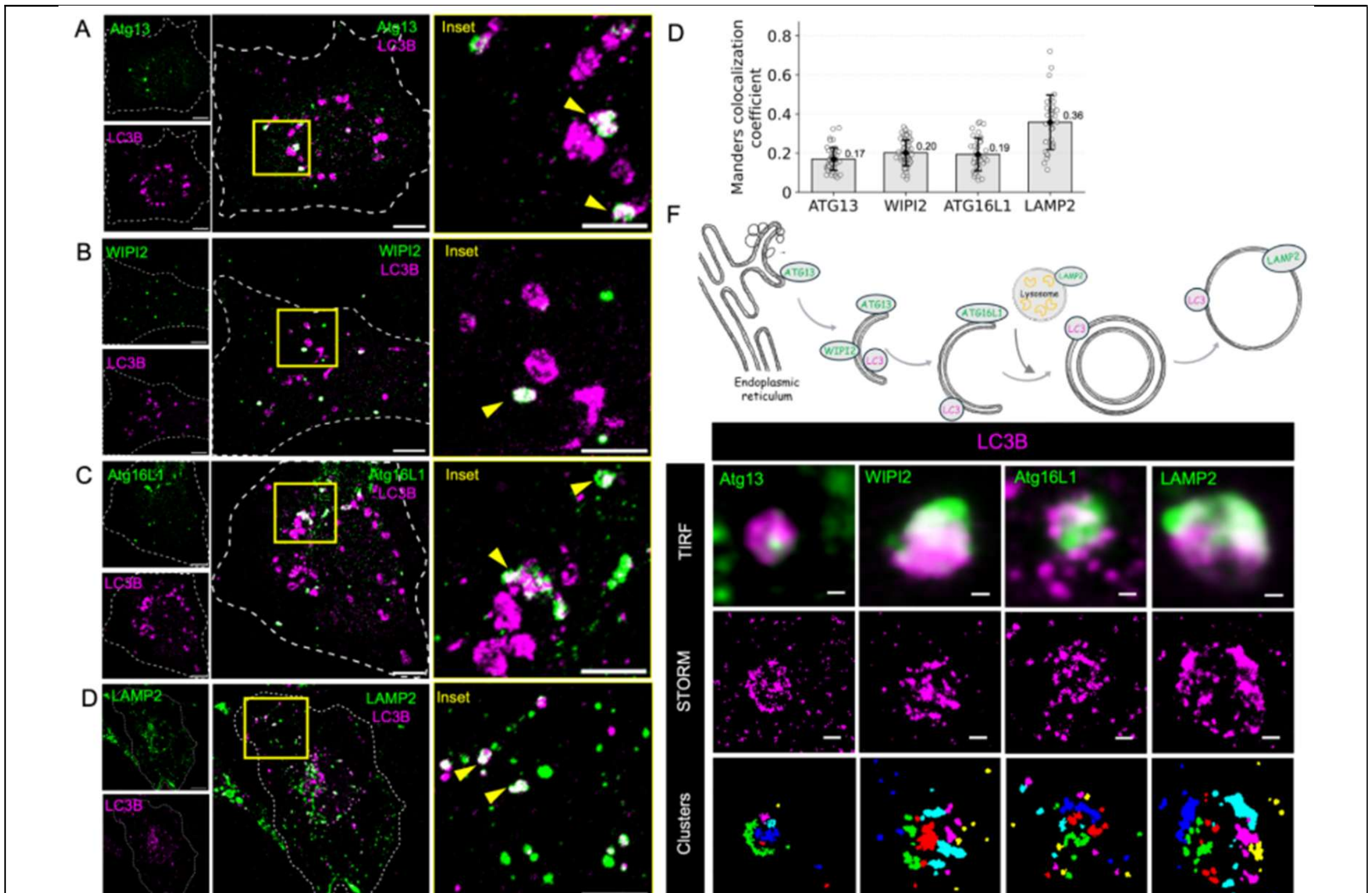


**Figure S11. LC3 forms nanoclusters on ATG13-, WIPI2-, ATG16L1-, and LAMP2-positive vesicles across early to late stages of autophagosome maturation.** (A–D) Representative dual-color confocal images of LC3B (magenta) with ATG13, WIPI2, ATG16L1, or LAMP2 (green). Yellow boxes indicate enlarged regions with arrowheads highlighting double-positive vesicles. (E) Manders colocalization coefficients quantify colocalization of LC3 with ATG13, WIPI2, ATG16L1, and LAMP2. Data are shown as mean ± SD from 51 cells (ATG13), 55 cells (WIPI2), 37 cells (ATG16L1), and 35 cells (LAMP2), pooled from two independent experiments. (F) Dual-color TIRF images of LC3 (magenta) with ATG13, WIPI2, ATG16L1, or LAMP2 (green), with corresponding STORM images showing the spatial organization of LC3 nanoclusters on marker-positive vesicles. Scale bars: 10 µm (full images), 5 µm (zoomed insets) for (A–D), and 500 nm for (F).

R3.*2). Figure 2G: It is unclear whether the large circular structures are bona fide autophagosomes. Please include three-color imaging with a well-established*

*autophagosomal membrane marker to confirm that the structures are autophagosomes. Sometimes a larger lipid droplet may appear, which may contain PI(3)P or nonspecific binding. It looks very different compared to Figure 2A.*

**Our Response: As the cross-commenting by Reviewer 1, we think that LC3 is a well-established marker of the autophagy process. However, to remove any further doubts regarding lipid droplets which may contain PI3P, we performed additional experiments.**

**We performed additional three-colour confocal imaging of endogenous LC3B, ATG13, and mCherry–2×FYVE. Importantly, we clearly identified vesicular structures that were positive for ATG13, LC3B, and mCherry–2×FYVE, confirming that the PI(3)P-enriched LC3-positive structures correspond to bona fide autophagic compartments. These additional data independently validate the autophagosomal identity of the structures analysed in Figure 2G.**

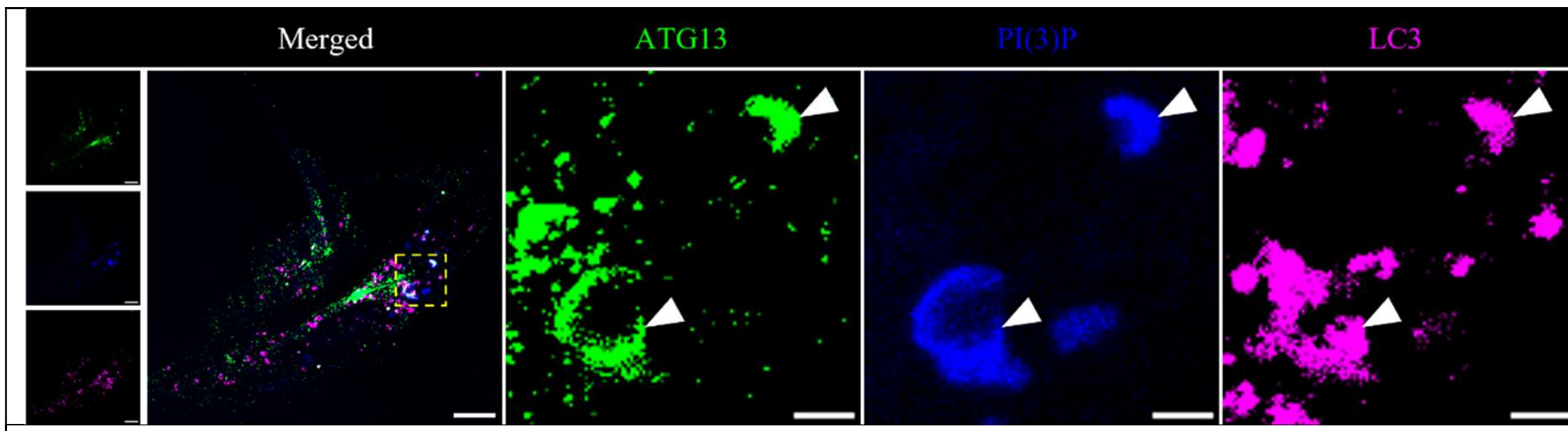


**Figure for Reviewer Response only:** Three-color confocal imaging of starved BS-C-1 cells overexpressed with 2X FYVE domain of HRS protein tagged with mCherry (blue) immuno-stained for endogenous ATG13 (green) and endogenous LC3B (magenta). Arrowheads indicate individual autophagosome positive for ATG13, PI(3)P and LC3B. Scale bar: 10 µm; Zoomed inset: 2 µm

R3.*3). The authors propose that LC3 clusters maintain a relatively constant size while increasing in number. If the intended interpretation is that LC3B recruitment to autophagosomes is progressive, further evidence is needed. An alternative explanation is that larger structures may be lower-density than the initial cluster and/or dissipate over time, leading to sparser distributions; this possibility should be tested and discusse*d.

**Our response:** **We thank the reviewer for this relevant suggestion and agree that there are two potential mechanisms that could explain why LC3 appear clusters on autophagosomes: (1) initial large, high-density LC3 structures on autophagosomes dissipate over time into smaller, sparser clusters, or (2) LC3 is progressively recruited to the autophagosome, forming new clusters over time.**

**To test the first possibility, we examined whether larger LC3 clusters have a higher LC3 density than smaller ones. We quantified the number of localizations within each cluster and divided it by the cluster area, and plotted this LC3 localization density against LC3**

**cluster size. We observed that cluster density remains largely independent of cluster size (r = -0.15), suggesting it is unlikely that initial high-density clusters dissipate over time into smaller, sparser ones.**

**Instead, we found a positive linear correlation (r = 0.62) between the total number of LC3 localizations on each autophagosome and the autophagosome size, confirming that more LC3 accumulates as the autophagosome grows in size. Notably, the LC3 cluster number showed a strong linear correlation with autophagosome size (r = 0.76), whereas cluster size showed a much weaker correlation (r = 0.36). Based on these data, we speculate that as the autophagosome matures, newly recruited LC3 organizes by progressively increasing the number of small clusters rather than expanding the size of existing ones.**

**We understand that this is an important point for the manuscript and we have now included these analyses in the revised manuscript as Figure S10 and also included in the main results section on Pg 6.**

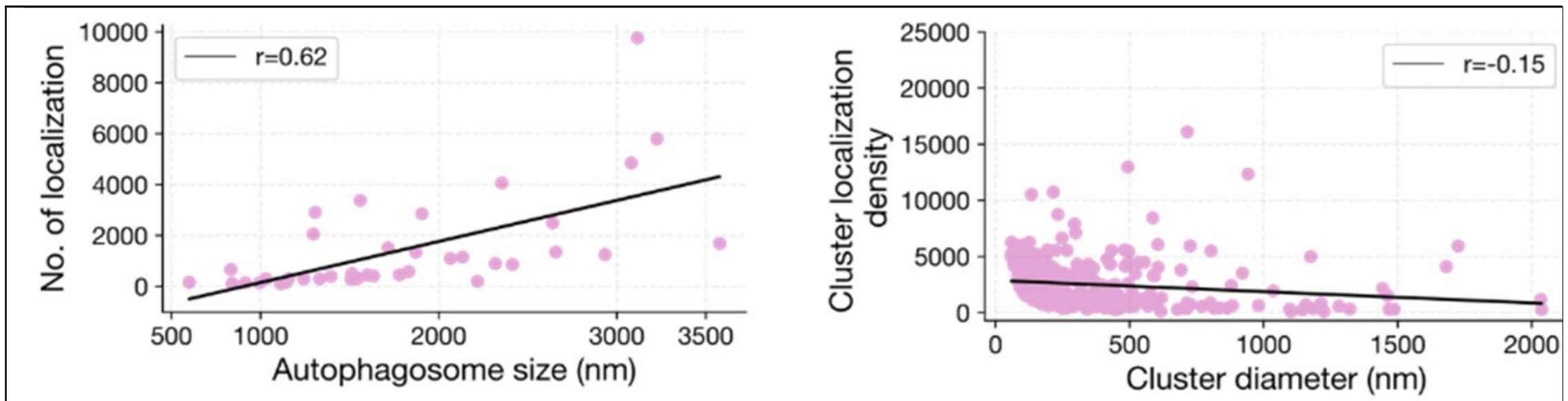


**Figure S10. LC3 is progressively recruited as the autophagosome grows.** (A) A scatter plot for the total number of LC3 localizations per autophagosome shows a positive correlation with autophagosome size, consistent with progressive LC3 recruitment during autophagosome growth. (B) A scatter plot between LC3 nanocluster localization density and LC3 cluster size shows a poor correlation.

*R3.4). Please include autophagy flux data using WB for all the LC3B mutants.*

**Our Response: To address the reviewer's suggestion, we performed additional autophagy flux analysis for the LC3 clustering mutants using p62 turnover as a biochemical readout in the presence and absence of Bafilomycin A1. Overall, the mutants exhibited broadly comparable p62 accumulation profiles relative to WT LC3B, indicating that bulk autophagic flux is largely preserved despite the pronounced nanoscale organizational defects observed in imaging experiments. While Loop6 mutants showed modestly elevated p62 accumulation, the overall changes remained subtle, suggesting that disruption of LC3 nanoclustering does not cause a complete blockade of basal autophagic flux. These observations are consistent with our interpretation that LC3 self-organization primarily contributes to higher-order spatial and cargo-associated functions of autophagy rather than globally impairing**

**autophagosome turnover. These results are now included as Figure S36 in the revised manuscript.**

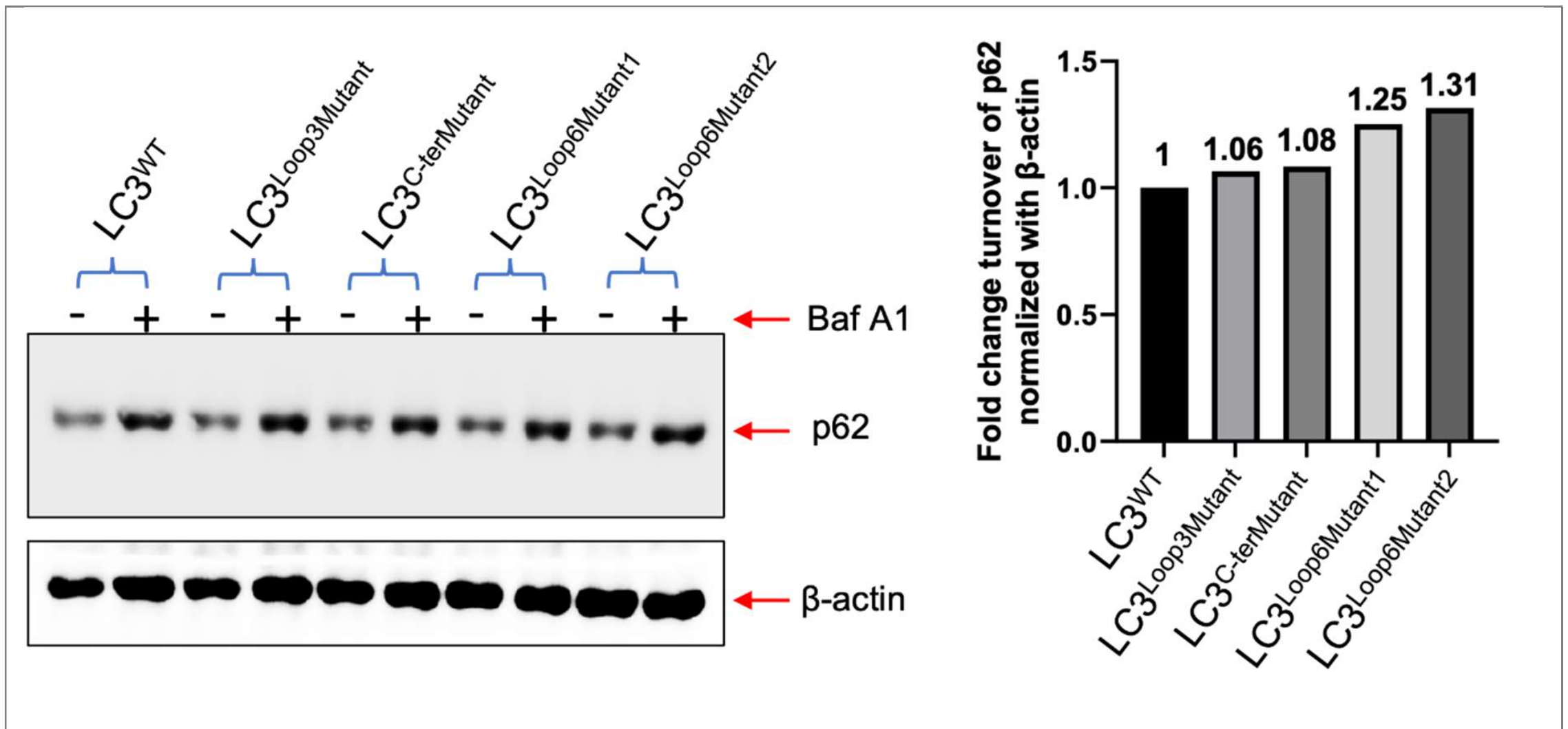


**Figure S36:** Immunoblot demonstrating autophagy flux as p62 accumulation as a readout. p62 turnover was assessed in cells treated with or without Baf A1 (25 nM) for 8 h. Immunoblotting of the cell lysate was performed with anti-p62 Ab.

R3.*5). Figure 5: Please use consistent zoom/magnification (and scale bars) across all panels to enable direct visual comparison.*

**Our response: We thank the reviewer for this suggestion. We have revised Figure 5 to maintain consistent magnification, zoom, and scale bars across all representative images, allowing direct visual comparison across panels and conditions.**

R3.*6). References: The references are not up to date; there are studies in which other groups observed LC3B clustering that need to be cited. (e.g., Science Advances 9 (39), eadh4094),*

**Our response: We thank the reviewer for highlighting this important and relevant study. The suggested reference (Banerjee et al., *Science Advances*, 2023) has now been incorporated into the revised manuscript, Discussion section (Pg 14). In this work, the authors employed quantitative PALM imaging to reveal the nanoscale organization of ULK1 structures and highlighted its association with other autophagy markers, including LC3, and their role in autophagosome maturation. We again thank the reviewer for pointing out this study, which is now included in the manuscript.**

R3.*7). Statistics/replicates: Several figures require error bars, statistical tests, and clear reporting of n (including biological replicates). Please specify the number of independent experiments and the statistical methods used in the figure caption.*

**Our response: We have now added details regarding the error bars, statistical tests, number of cells analyzed, and the number of independent experiments in the corresponding figure legends. The figure legends for Figures 2, 5, 6, S9, S10, S11, S33, and S35 now explicitly include information on the biological and technical replicates used for each experiment. In addition, detailed statistical information for each experiment has been provided separately as a CSV file.**

Dear Dr. Thukral

Thank you for the submission of your revised manuscript to EMBO reports. We have now received the full set of referee reports that is copied below.

As you will see, all referees are very positive about the study and request only minor changes to clarify text and figures.

From the editorial side, there are also a few things that we need before we can proceed with the official acceptance of your study.

- Please remove the figures from the manuscript and place the figure legends after the References at the end.

- Data and code availability needs to be changed to Data Availability. Please remove the statement "Additional information needed to reanalyze the data reported in this paper can be obtained from the Lead Contact upon request." This section should exclusively refer to the data deposited at public repositories.

- Please provide a 'Disclosure and competing interests statement'. For more information see https://link.springer.com/journal/44319/submission-guidelines#conflictsofinterest

- The references need to be alphabetical, not numerical; et al needs to be used after 10 author names; DOIs should only be used for preprints and datasets that have not been published yet.

- Please provide a complete author checklist, which you can download from our author guidelines. Please insert information in the checklist that is also reflected in the manuscript. The completed author checklist will also be part of the Review Process File.

- The information on funding provided in the manuscript text and the online submission system must be congruent. We note that Prime Ministers Research Fellowship (PMRF) is missing as a separate entry in the system.

- Please provide callous for Movies EV10-16 in the text.

- Appendix: please provide the Appendix file as PDF instead of the word file. The title page should only state "Appendix to Ms Title" and a table of contents listing each item and its page number. The correct nomenclature should be Appendix Figure S1, etc. and Appendix Table S1, etc. Please update this nomenclature in the file and in the manuscript text callouts. Also here, some of the figures appear to have rather low resolutin, e.g., Appendix Figure S2, S4., S18, S37 etc Please carefully check all figures for resolution.
- Moreover, I noticed that you have 37 Appendix figures, some of which show only one Westernblot. You might want to consider reducing the number of figures by merging some of them.

- Movies: please remove the legends from the Appendix file and provide eachin a separate text file; then each movie should be zipped together with its legend so that we have 16 zip folders Movie EV1 - Movie EV16.

- Appendix Tables S1 and S2 could be part of the Reagents and Tools table (see next point).

- The Methods section will include a Reagents and Tools Table (listing key reagents, experimental models, software and relevant equipment and including their sources and relevant identifiers). You can download a template (.docx) for the Reagents and Tools in our author guidelines. Please fill it and upload it as separate file, file type Reagents table.

- Materials and Methods should be Methods

-) During our routine image checks, we noticed the following:

- Figure 1C, Replica 3 at 1 msec looks similar to LC3B, 1 msec in Figure S12
- Figure 1C, Replica 1 and Replica 3 at 5 msec look very similar to each other and to LC3B, 2 msec in Figure S12.
Please carefully check these images and the composition of these figure panels and clarify the potential reuse.

-) Please address the following points in the figure legends and please highlight the changes:
- Please provide the exact p values in the legends of figures 5B,C,E,G; 6B,C. [Here, I note that you have supplied a separate sheet for all exact p-values, which is fine but please refer to it in the figure legends]
- Please note that the box plots need to be defined in terms of minima, maxima, centre, bounds of box and whiskers, and percentile in the legends of figures 2J; 3F; 5B,C,E; Figure S35.
- Please note that information related to n is missing in the legends of figure 3F.
- Please note that the white arrows are not defined in the legend of figure 6A. This needs to be rectified.

- Looking at Figure 1 without zooming in, the panels appear to have rather low resolution and appear 'fuzzy". The same is true for Figure 3, B, F, Figure 4C, Figure 5G, C, E, G, Figure 6B, C

- We do a spot-check of the supplied source data and it seems that the source data images for Figure 1C do not match with those shown in the figure. SIM3 images match Replica 2. SIM2 images match Replica 1 but the image at 5ms seems not to correspond.
- For Figure 2B, it appears as if the source data files specified "XZ" actually correspond to "YZ" in the figure an vice versa. Please make sure everything matches between source data and figure panels.

- The .xls files that you provided as source data files called "ALL FIGURES all p values" and "All Figures N values" - if this is indeed meant to be supplied as source data, then each file would need to be in its corresponding figure folder. I would suggest to upload these as Datasets instead (Dataset EV1 and EV2) and to refer to these either in the figure legends or the methods, as appropriate. Datasets need a legend in a separate tab of the .xls file.

- The file in the source data folder for Figure 4 is not clearly labeled and it remains unclear to which of the panels it belongs.

- The source data need to be upldd as one zip folder per figure, please.

- You state in the point-by-point response that you used AI for grammar correction. Please specify the use of AI for grammar checking and detail the tool employed in the methods section.

- Your response to the referees contains unpublished data in form of reviewer figures (page 23). We publish the referee reports and your response to these alongside the paper as Peer Review File. Please let us know in case you want to remove any of the reviewer figures. Please note that all data in the Peer Review File counts as published and that you would have to cite this document were your to re-use these in future publications.

- Please describe your findings in the abstract in present tense.

- Finally, EMBO Reports papers are accompanied online by
A) a short (1-2 sentences) summary of the findings and their significance,
B) 2-3 bullet points highlighting key results and
C) a schematic summary figure that provides a sketch of the major findings (not a data image).
Please provide the summary figure as a separate file in PNG or JPG format at a size of 550x300 pixels (width x height). Please note that the size is rather small and that text needs to be readable at the final size. Please send us this information along with the revised manuscript.

I look forward to seeing a new revised version of your manuscript as soon as possible.

Kind regards,

Martina Rembold, PhD
Senior Editor
EMBO reports

=================

Referee #1:

The authors have satisfactorily addressed my concerns.

Referee #2:

The authors have done a very impressive job with this revision. This is an outstanding contribution to the autophagy field and puts the role and function of ATG8 proteins into a new and deeper perspective.

Referee #3:

The authors addressed my comments and provided additional data. I have some comments that need to be addressed before its publication.

Figure 2
For Fig. 2A, it would be helpful to include the cell boundary, similar to what is shown in Fig. 2G. For Fig. 2D, the presentation would be more informative if error bars were included based on the three replicates. If feasible, presenting the data on a per-cell basis may provide an even more meaningful comparison. The same suggestion applies to Fig. 2F.
For Fig. 2G, the main image and the inset show the same field at different magnifications. I do not understand why this is needed.
Fig. 2J appears considerably larger than the other panels. It may be more appropriate to present it as an inset within Fig. 2I.
Figure 5
In Fig. 5D, the images for the LC3 C-terminal mutant and LC3 Loop6 mutant 1 appear different from those of the wild type and the other mutants, particularly in terms of expression level or image appearance. For the tracking analysis, cells with comparable expression levels should ideally be analyzed. If expression levels vary substantially among constructs, this should be clearly stated in the Methods and considered when interpreting the results.
If all images were acquired at the same magnification, it may not be necessary to include a scale bar in every panel. A single clearly defined scale bar may improve the overall appearance, although there is no strict requirement.
The tracking analysis and the conclusions drawn from it are currently unclear and would benefit from more coherent presentation. The authors conclude that FYCO1-LC3 colocalization decreases in the mutants; however, it is not clear how the average speed remains unchanged. If the run length is reduced, pause duration is increased, and speed remains unchanged, does this indicate more spatially confined or multidirectional movement rather than reduced motor velocity?
Minor comments
In Fig. 6, the inset image does not contain a scale bar.
It would also be good to add a paragraph describing the limitations of the study.

Please clarify whether the images were acquired using TIRF or HILO illumination. If HILO was used, it would be helpful to report the illumination angle.

**Point-by-point Response to Editor's and Reviewers' Comments**

In the following, we address the comments (in *italics*) point by point and highlight changes made in this version of revised manuscript (in red).

**Title:**" LC3 forms functional nanoclusters"
**Manuscript #**: EMBOR-2025-63271V2
**Authors**: Fatihi et. al.,

**Editor summary:** *As you will see, all referees are very positive about the study and request only minor changes to clarify text and figures. From the editorial side, there are also a few things that we need before we can proceed with the official acceptance of your study.*

**Our response: We are delighted to know that all three referees acknowledged our efforts during revision and have shared their positive responses. The minor changes proposed by reviewer #3 have been fully addressed. Additionally, the editorial comments have also been completed, and point-by-point response is mentioned below.**

*- Please remove the figures from the manuscript and place the figure legends after the References at the end.*

**Our response: As required, the figure legends are placed after the References.**

*- Data and code availability needs to be changed to Data Availability. Please remove the statement "Additional information needed to reanalyze the data reported in this paper can be obtained from the Lead Contact upon request." This section should exclusively refer to the data deposited at public repositories.*

**Our response: As required, the statement in the 'Data Availability' has now been removed.**

*- Please provide a 'Disclosure and competing interests statement'. For more information see* https://link.springer.com/journal/44319/submission-guidelines#conflictsofinterest

**Our response: We thank the editor for this suggestion. A Disclosure and Competing Interests statement has now been included in the manuscript under the Ethics Declarations section: *The authors declare no competing interests.***

*- The references need to be alphabetical, not numerical; et al needs to be used after 10 author names; DOIs should only be used for preprints and datasets that have not been published yet.*

**Our response: We have revised the reference list accordingly.**

- Please provide a complete author checklist, which you can download from our author guidelines. Please insert information in the checklist that is also reflected in the manuscript. The completed author checklist will also be part of the Review Process File.
**Our response: A fully completed author checklist has been uploaded as a part of the Review Process File.**

- The information on funding provided in the manuscript text and the online submission system must be congruent. We note that Prime Ministers Research Fellowship (PMRF) is missing as a separate entry in the system.
**Our response: We thank the editor for this suggestion. We attempted to add the Prime Minister's Research Fellowship (PMRF) as a separate funding entry in the online submission system. However, the submission portal does not accept or display PMRF as a funding source. As PMRF is a national fellowship awarded to doctoral students, rather than a research grant, we have acknowledged it in the Acknowledgements section of the manuscript.**

*- Please provide callous for Movies EV10-16 in the text.*
**Our Response: As required by the editor, the callouts have been added to the results section.**

*- Appendix: please provide the Appendix file as PDF instead of the word file. The title page should only state "Appendix to Ms Title" and a table of contents listing each item and its page number. The correct nomenclature should be Appendix Figure S1, etc. and Appendix Table S1, etc. Please update this nomenclature in the file and in the manuscript text callouts. Also here, some of the figures appear to have rather low resolutin, e.g., Appendix Figure S2, S4, S18, S37 etc Please carefully check all figures for resolution.*

**Our Response: As required, the earlier submitted S.I document has now been aligned with required format from EMBO as Appendix file. It includes the Table of figures, corrected nomenclature, and the same has been updated in the manuscript text callouts. Additionally, Appendix Figure S2, S4, S18, and S37 have been updated for resolution.**

- Moreover, I noticed that you have 37 Appendix figures, some of which show only one Westernblot. You might want to consider reducing the number of figures by merging some of them.

**Our response: We thank the reviewer for the suggestions. As suggested, we have now merged several appendix figures, reducing the number of figures from 37 to 24. The details include:**

- **Appendix Figure S2 combines former Figures S2 + S3**
- **Appendix Figure S4 combines former Figures S5 + S6 + S7**
- **Appendix Figure S11 combines former Figures S18 + S19**
- **Appendix Figure S18 combines former Figures S26 + S27 + S28**
- **Appendix Figure S19 combines former Figures S29 + S30**
- **Appendix Figure S21 combines former Figures S32 + S33**
- **Appendix Figure S22 combines former Figures S34 + S35**
- **New Appendix Figure S6 for per-cell box plots plotted from Figure 2D/2F data**
- **Figures S11, S12-S14 and S17 are now Expanded view figures 1-3.**

*- Movies: please remove the legends from the Appendix file and provide each in a separate text file; then each movie should be zipped together with its legend so that we have 16 zip folders Movie EV1 - Movie EV16.*

**Our response: We have revised the movie files from Movie EV1- Movie EV16, as requested.**

*- Appendix Tables S1 and S2 could be part of the Reagents and Tools table (see next point).*

**Our response: This is a good suggestion. The tables have been removed from Appendix and have been updated in the Reagents and Tools table.**

*- The Methods section will include a Reagents and Tools Table (listing key reagents, experimental models, software and relevant equipment and including their sources and relevant identifiers). You can download a template (.docx) for the Reagents and Tools in our author guidelines. Please fill it and upload it as separate file, file type Reagents table.*

**Our response: In aligned with required files, we have provided the Reagents and Tools Table.**

*- Materials and Methods should be Methods*

**Our response: We have revised the title.**

*-) During our routine image checks, we noticed the following:*
*- Figure 1C, Replica 3 at 1 msec looks similar to LC3B, 1 msec in Figure S12*

*Please carefully check these images and the composition of these figure panels and clarify the potential reuse.*

*Figure 1C, Replica 3 at 1 msec looks similar to LC3B, 1 msec in Figure S12*

**Our response: The observation is correct. The image shown for the 1 μs time point was intentionally reused in both figures. Figure 1C presents representative snapshots of the wild-type LC3B simulation, while Figure S12 compares the corresponding wild-type LC3B simulation with those of other LC3 homologs. Since the same wild-type simulation is being referenced, the 1 μs snapshot is intentionally identical in both figures. To avoid any ambiguity, we have revised the Figure S12 legend to explicitly state that the 1 μs LC3B snapshot is the same image as that shown in Figure 1C.**

*- Figure 1C, Replica 1 and Replica 3 at 5 msec look very similar to each other and to LC3B, 2 msec in Figure S12.*

**We thank the editor for identifying this issue. There are two separate points:**

1. **Figure 1C (Replica 1 and Replica 3 at 5 μs): This was an inadvertent error during figure assembly, and we sincerely apologize. The duplicated panel has now been replaced with the correct representative snapshot in the revised Figure 1C.**
2. **Figure S12 (LC3B, 2 μs): The apparent similarity arises because the conformations at 2 μs are highly similar. To avoid any ambiguity, we have replaced the representative image with a snapshot from 2.002 μs, which is clearly distinguishable while remaining representative of the simulation.**

*-) Please address the following points in the figure legends and please highlight the changes:*
*- Please provide the exact p values in the legends of figures 5B,C,E,G; 6B,C. [Here, I note that you have supplied a separate sheet for all exact p-values, which is fine but please refer to it in the figure legends]*

**Our response: As suggested, we have updated the figure legends with exact p-values.**

*- Please note that the box plots need to be defined in terms of minima, maxima, centre, bounds of box and whiskers, and percentile in the legends of figures 2J; 3F; 5B,C,E; Figure S35.*

**Our response: As suggested, we have updated the figure legends to include a complete description of the box plots elements.**

*- Please note that information related to n is missing in the legends of figure 3F.*

**Our response: We thank the reviewer for pointing this out. The Figure 3F legend has been updated to include the value of *n***

*- Please note that the white arrows are not defined in the legend of figure 6A. This needs to be rectified.*
**Our response: As suggested, the figure legend has been updated to indicate that the white arrows mark the regions of interest (ROIs) shown in the corresponding magnified insets.**

*- Looking at Figure 1 without zooming in, the panels appear to have rather low resolution and appear 'fuzzy". The same is true for Figure 3, B, F, Figure 4C, Figure 5G, C, E, G, Figure 6B, C*

**Our response: We have comprehensively worked on the Figure resolution and now the modified figure panels have been improved to enhance clarity.**

*- We do a spot-check of the supplied source data and it seems that the source data images for Figure 1C do not match with those shown in the figure. SIM3 images match Replica 2. SIM2 images match Replica 1 but the image at 5ms seems not to correspond.*

**Our response: We apologise for this inadvertent error. All the files related to Figure 1C now correspond to appropriate source data files.**

*- For Figure 2B, it appears as if the source data files specified "XZ" actually correspond to "YZ" in the figure an vice versa. Please make sure everything matches between source data and figure panels.*

**Our response: Thank you for pointing out this error. The labels have been corrected and updated in the source data files.**

*- The .xls files that you provided as source data files called "ALL FIGURES all p values" and "All Figures N values" - if this is indeed meant to be supplied as source data, then each file would need to be in its corresponding figure folder. I would suggest to upload these as Datasets instead (Dataset EV1 and EV2) and to refer to these either in the figure legends or the methods, as appropriate. Datasets need a legend in a separate tab of the .xls file.*

**Our response: Thank you for the suggestion. We have now provided the exact *P* values and sample size (*N*) information as Dataset EV1 and Dataset EV2, respectively. These datasets are referred to in the Methods section.) information as Dataset EV1 and Dataset EV2, respectively. These datasets are referred to in the Methods section.**

*- The file in the source data folder for Figure 4 is not clearly labeled and it remains unclear to which of the panels it belongs.*

**Our response: The source data file for Figure 4 has been renamed to match the corresponding figure panel.**

*- The source data need to be upldd as one zip folder per figure, please.*

**Our response: As requested, the source data have been organized and uploaded as one ZIP folder per figure.**

*- You state in the point-by-point response that you used AI for grammar correction. Please specify the use of AI for grammar checking and detail the tool employed in the methods section.*

**Our response: As requested, we have now specified our use of AI for grammar correction in the Methods section. The relevant statement reads:**

**“Artificial Intelligence tools were used to assist with grammar and language correction. All content was subsequently reviewed and verified by the authors, who take full responsibility for the final text.”**

*- Your response to the referees contains unpublished data in form of reviewer figures (page 23). We publish the referee reports and your response to these alongside the paper as Peer Review File. Please let us know in case you want to remove any of the reviewer figures. Please note that all data in the Peer Review File counts as published and that you would have to cite this document were your to re-use these in future publications.*
**Our response: We confirm that we are glad for the reviewer figures to be included in the published Peer Review File.**

*- Please describe your findings in the* **abst***ract in present tense.*
**Our response: As suggested, we have rephrased the abstract so that all findings are now described in the present tense. The single exception is the methods statement referring to mutant generation, which we have retained in the past tense ("generated"), as is conventional for describing methodology.**

**- Finally, EMBO Reports papers are accompanied online by A) a short (1-2 sentences) summary of the findings and their significance, B) 2-3 bullet points highlighting key results and**

**Our response:**

**Summary:**

**Combining super-resolution microscopy with molecular dynamics simulations, we show that LC3B self-assembles through a defined clustering interface. This organization is essential for autophagosome motility and the clearance of bacterial cargo.**

**Key results**

- **LC3 self-assembles into ~150 nm nanoclusters that preferentially localize to PI(3)P-enriched regions of the autophagosome membrane.**
- **Clustering is mediated by a specific interface involving loop 6 and helix α4, defined by an alternating hydrophobic–polar motif.**

- **Disrupting LC3 clustering impairs FYCO1-dependent autophagosome motility and bacterial cargo clearance, increasing intracellular bacterial survival.**

*C) a schematic summary figure that provides a sketch of the major findings (not a data image).*
*Please provide the summary figure as a separate file in PNG or JPG format at a size of 550x300 pixels (width x height). Please note that the size is rather small and that text needs to be readable at the final size. Please send us this information along with the revised manuscript.*

**Graphical Abstract**

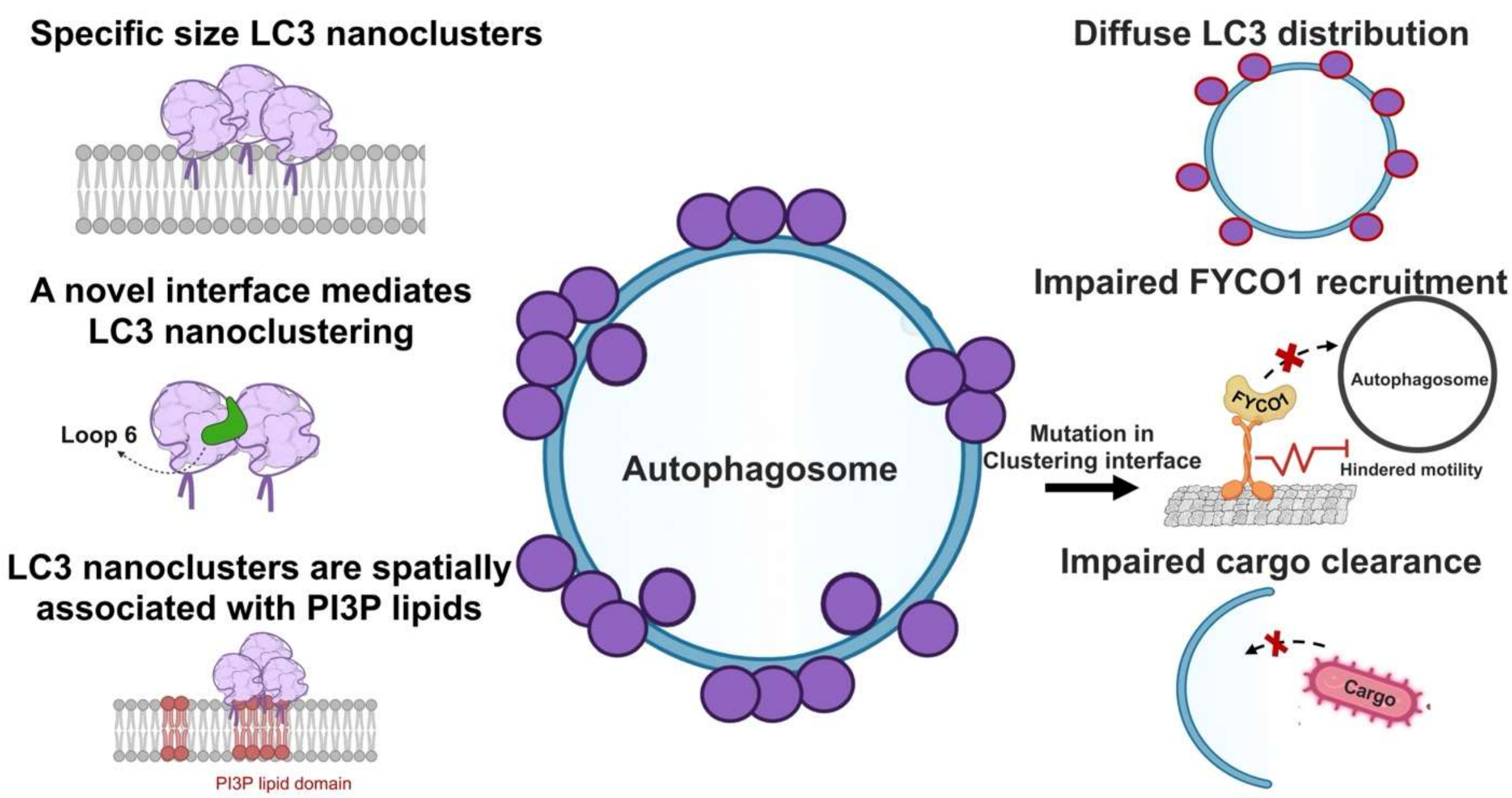


**Referee #3:**

**The authors addressed my comments and provided additional data. I have some comments that need to be addressed before its publication.**
**Our response: We thank you for your time and effort in improving our manuscript. The comments in this round have been addressed and are detailed point by point below.**

*Figure2*

-For Fig. 2A, it would be helpful to include the cell boundary, similar to what is shown in Fig. 2G.

**Our Response: We thank the reviewer for pointing out this change. As per the suggestion, we have included a cell boundary for Figure 2A, like other figures.**

*-For Fig. 2D, the presentation would be more informative if error bars were included based on the three replicates. If feasible, presenting the data on a per-cell basis may provide an even more meaningful comparison. The same suggestion applies to Fig. 2F.*

**Our Response: We thank the reviewer for this constructive suggestion to show the distribution and error bars across our biological replicates. As suggested, we have replotted the data from Figure 2D and Figure 2F in a cell-averaged format (showing the median and SD from cell-wise distributions) and have added these graphs to the manuscript as a new Appendix Figure S6 (also pasted here for your reference).**

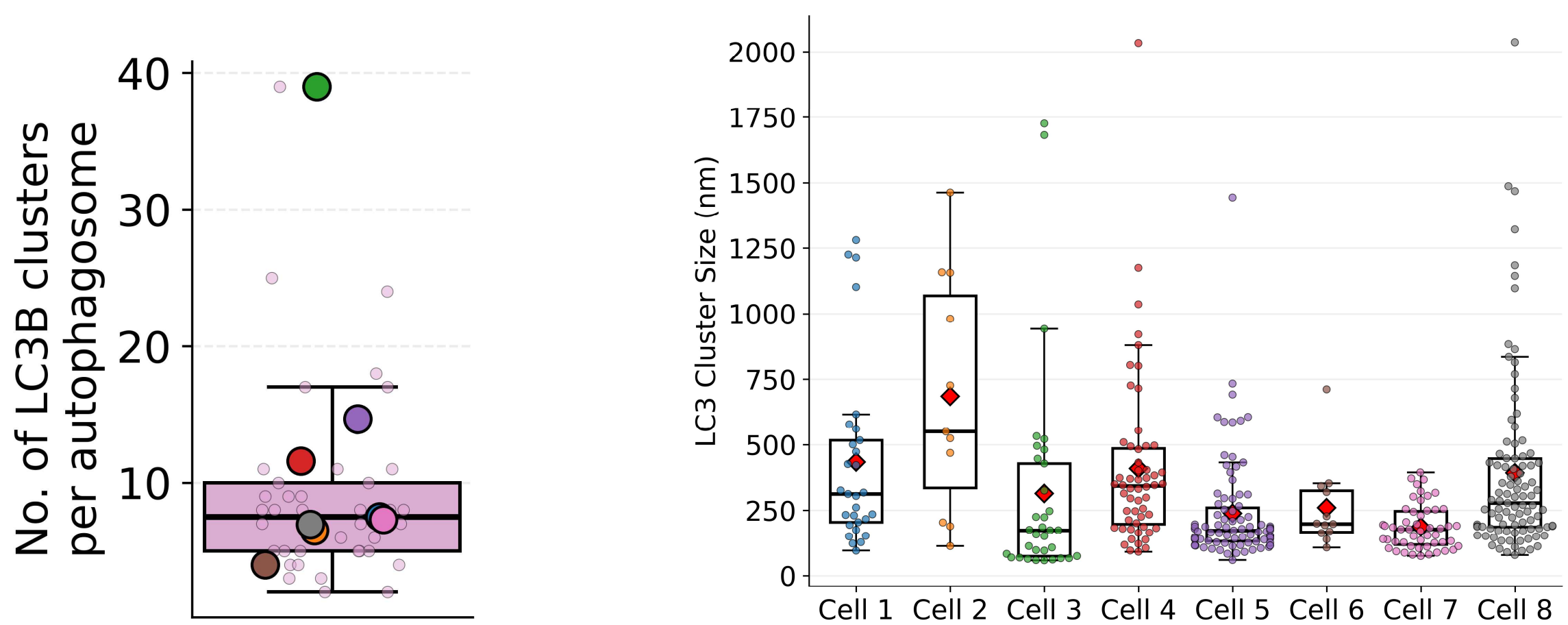


Appendix Figure S6: A) Average number of LC3B nanoclusters per autophagosome across N = 8 cells. Data replotted from Figure 2D as Box plots. Large circles show individual cell means, overlaid on single-autophagosome data. Box plots represent the median, interquartile range, and SD across all cells. B) Data replotted from Figure 2F as Box plots showing the size distribution of the LC3B cluster size across 8 individual cells. Red diamonds mark the cell mean, highlighting single-vesicle size variation within individual cells.

-For Fig. 2G, the main image and the inset show the same field at different magnifications. I do not understand why this is needed.

**Our Response: We apologize for the confusion with the inset. To clarify, we have now labelled the inset as Total Internal Reflection Fluorescence (TIRF), which corresponds to the diffraction-limited image of the same field of view as the STORM full image, and the inset of Figure 2G has been labelled accordingly.**

*-Fig. 2J appears considerably larger than the other panels. It may be more appropriate to present it as an inset within Fig. 2I.*

**Our Response: We thank the reviewer for this suggestion. We did consider presenting Fig. 2J as an inset within Fig. 2I. However, we found that this arrangement disrupts the visual balance of the figure and reduces the legibility of the data in panel 2J. We have therefore retained the original layout.**

*Figure5*

*-In Fig. 5D, the images for the LC3 C-terminal mutant and LC3 Loop6 mutant 1 appear different from those of the wild type and the other mutants, particularly in terms of expression level or image appearance. For the tracking analysis, cells with comparable expression levels should ideally be analyzed. If expression levels vary substantially among constructs, this should be clearly stated in the Methods and considered when interpreting the results. If all images were acquired at the same magnification, it may not be necessary to include a scale bar in every panel. A single clearly defined scale bar may improve overall appearance, although there is no strict requirement.*

**Our Response: We agree with the reviewer that there may be differences in expression levels. We carefully selected cells with comparable expression levels for our analyses. However, small differences in expression cannot be entirely ruled out. Following the reviewer's suggestion, we have added the following statement to the manuscript on Page no. 22: "We aimed to select cells with a comparable level of LC3 expression. However, we cannot rule out the possibility of variable construct expression contributing to differences in motility parameters."**

*-The tracking analysis and the conclusions drawn from it are currently unclear and would benefit from more coherent presentation. The authors conclude that FYCO1-LC3 colocalization decreases in the mutants; however, it is not clear how the average speed remains unchanged. If the run length is reduced, pause duration is increased, and speed remains unchanged, does this indicate more spatially confined or multidirectional movement rather than reduced motor velocity?*

**Our Response: We thank the reviewer for this insightful comment. As correctly pointed, the reduced run length and increased pause duration alongside unchanged speed reflect a disruption of directed, motor-driven transport i.e., more confined/diffusive movement rather than reduced motor velocity. Speed remains unchanged because it is computed exclusively within active (directed) segments**

**and is therefore independent of pause frequency and duration. We have clarified this in the manuscript (Page 23):**

**"Run length was defined as the net displacement of each active (directed) segment, pause duration as the cumulative time spent in confined/diffusive phases ($R < 0.7$), and speed as the instantaneous velocity averaged over active transport phases only. As speed was computed exclusively within directed segments, it is independent of the frequency and duration of pauses."**

**Minor comments**

*-In Fig. 6, the inset image does not contain a scale bar.*

**Our Response: We thank the reviewer for pointing out this error. We have now updated Figure 6 to include a scale bar on all insets.**

*It would also be good to add a paragraph describing the limitations of the study.*

**Our Response: We thank the reviewer for this suggestion. The following text has been added in the manuscript highlighting the same.**

**"Our study also has certain limitations. First, although our simulations identify the Loop6 interface, the precise stoichiometry and kinetics of nanocluster assembly remain to be established, as these depend on the topology of the clusters on the inner and outer membranes of the autophagosome. Second, we cannot fully exclude a contribution of endogenous LC3/GABARAP family members to the observed phenotypes. Finally, while we observe a strong association between LC3 nanoclusters and PI3P-enriched membrane domains, the broader involvement of the autophagic machinery in organizing these PI3P–LC3 clusters, and whether other factors spatially co-cluster within these domains, remain to be examined."**

*Please clarify whether the images were acquired using TIRF or HILO illumination. If HILO was used, it would be helpful to report the illumination angle.*

**Our Response: All live-cell and STORM imaging datasets (including the single-particle tracking) were acquired using Total Internal Reflection Fluorescence (TIRF) illumination. We have updated the Materials and Methods section on Page No. 22 to explicitly state that TIRF illumination was utilized.**

Dr. Lipi Thukral
CSIR Institute of Genomics and Integrative Biology, New Delhi, India
Systems Biology group
CSIR-IGIB
Mathura Road
New Delhi, Delhi 110020
India

Dear Lipi,

Thank you for sending the updated manuscript file and synopsis image. I have uploaded both files to your submission. With this I am very pleased to accept your manuscript for publication in the next available issue of EMBO reports. Thank you for your contribution to our journal and congratulations to a great paper!

Your manuscript will be processed for publication by EMBO Press. It will be copy edited and you will receive page proofs prior to publication. Please note that you will be contacted by Springer Nature Author Services to complete licensing and payment information.

You may qualify for financial assistance for your publication charges - either via a Springer Nature fully open access agreement or an EMBO initiative. Check your eligibility: https://link.springer.com/journal/44319/how-to-publish-with-us

Should you be planning a Press Release on your article, please notify the production team via this form in order to coordinate publication and release dates: https://forms.cloud.microsoft/e/eH4A8JVP6g

If you have any questions, please do not hesitate to contact the Editorial Office. Thank you for your contribution to EMBO Reports.

Kind regards,

Martina

Martina Rembold, PhD
Senior Editor
EMBO reports

------------------------------------------------

>>> Please note that it is EMBO Reports policy for the transcript of the editorial process (containing referee reports and your response letter) to be published as an online supplement to each paper. If you do NOT want this, you will need to inform the Editorial Office via email immediately. More information is available here: https://link.springer.com/partners/embo-press/editorial-policies#Peer%20review

## EMBO Press Author Checklist

| | |
|---|---|
| Corresponding Author Name: Lipi Thukral and Nitin Mohan | |
| Journal Submitted to: EMBO reports | |
| Manuscript Number: EMBOR-2025-63271V2 | |

**USEFUL LINKS FOR COMPLETING THIS FORM**
The EMBO Journal - Author Guidelines
EMBO Reports - Author Guidelines
Molecular Systems Biology - Author Guidelines
EMBO Molecular Medicine - Author Guidelines

**Reporting Checklist for Life Science Articles (updated January**
This checklist is adapted from Materials Design Analysis Reporting (MDAR) Checklist for Authors. MDAR establishes a minimum set of requirements in transparent reporting in the life sciences (see Statement of Task: 10.31222/osf.io/9sm4x). Please follow the journal's guidelines in preparing your manuscript.
**Please note that a copy of this checklist will be published alongside your article.**

**Abridged guidelines for figures**
**1. Data**
The data shown in figures should satisfy the following conditions:
- the data were obtained and processed according to the field's best practice and are presented to reflect the results of the experiments in an accurate and unbiased manner.
- ideally, figure panels should include only measurements that are directly comparable to each other and obtained with the same assay.
- plots include clearly labeled error bars for independent experiments and sample sizes. Unless justified, error bars should not be shown for technical
- if n<5, the individual data points from each experiment should be plotted. Any statistical test employed should be justified.
- Source Data should be included to report the data underlying figures according to the guidelines set out in the authorship guidelines on Data

**2. Captions**
Each figure caption should contain the following information, for each panel where they are relevant:
- a specification of the experimental system investigated (eg cell line, species name).
- the assay(s) and method(s) used to carry out the reported observations and measurements.
- an explicit mention of the biological and chemical entity(ies) that are being measured.
- an explicit mention of the biological and chemical entity(ies) that are altered/varied/perturbed in a controlled manner.
- the exact sample size (n) for each experimental group/condition, given as a number, not a range;
- a description of the sample collection allowing the reader to understand whether the samples represent technical or biological replicates (including how many animals, litters, cultures, etc.).
- a statement of how many times the experiment shown was independently replicated in the laboratory.
- definitions of statistical methods and measures:
  - common tests, such as t-test (please specify whether paired vs. unpaired), simple $\chi 2$ tests, Wilcoxon and Mann-Whitney tests, can be unambiguously identified by name only, but more complex techniques should be described in the methods section;
  - are tests one-sided or two-sided?
  - are there adjustments for multiple comparisons?
  - exact statistical test results, e.g., P values = x but not P values < x;
  - definition of 'center values' as median or average;
  - definition of error bars as s.d. or s.e.m.

**Please complete ALL of the questions below.**
**Select "Not Applicable" only when the requested information is not relevant for your study.**

**Materials**

| Newly Created Materials | Information included in the manuscript? | In which section is the information available? (Reagents and Tools Table, Materials and Methods, Figures, Data Availability Section) |
|---|---|---|
| New materials and reagents need to be available; do any restrictions apply? | Yes | Reagents and Tools Table |

| Antibodies | Information included in the manuscript? | In which section is the information available? (Reagents and Tools Table, Materials and Methods, Figures, Data Availability Section) |
|---|---|---|
| For antibodies provide the following information:<br>- Commercial antibodies: RRID (if possible) or supplier name, catalogue number and or/clone number<br>- Non-commercial: RRID or citation | Yes | Reagents and Tools Table; Materials and Methods |

| DNA and RNA sequences | Information included in the manuscript? | In which section is the information available? (Reagents and Tools Table, Materials and Methods, Figures, Data Availability Section) |
|---|---|---|
| Short novel DNA or RNA including primers, probes: provide the sequences. | Not Applicable | |

| Cell materials | Information included in the manuscript? | In which section is the information available? (Reagents and Tools Table, Materials and Methods, Figures, Data Availability Section) |
|---|---|---|
| **Cell lines: Provide species information, strain. Provide accession number in repository OR supplier name, catalog number, clone number, and/OR RRID.** | Yes | Materials and Methods |
| **Primary cultures: Provide species, strain, sex of origin, genetic modification status.** | Not Applicable | |
| Report if the cell lines were recently authenticated (e.g., by STR profiling) and tested for mycoplasma contamination. | Not Applicable | |

| Experimental animals | Information included in the manuscript? | In which section is the information available? (Reagents and Tools Table, Materials and Methods, Figures, Data Availability Section) |
|---|---|---|
| **Laboratory animals or Model organisms: Provide species, strain, sex, age, genetic modification status. Provide accession number in repository OR supplier name, catalog number, clone number, OR RRID.** | Not Applicable | |
| **Animal observed in or captured from the field: Provide species, sex, and age where possible.** | Not Applicable | |
| Please detail housing and husbandry conditions. | Not Applicable | |

| Plants and microbes | Information included in the manuscript? | In which section is the information available? (Reagents and Tools Table, Materials and Methods, Figures, Data Availability Section) |
|---|---|---|
| **Plants: provide species and strain, ecotype and cultivar where relevant, unique accession number if available, and source (including location for collected wild specimens).** | Not Applicable | |
| **Microbes: provide species and strain, unique accession number if available, and source.** | Yes | Materials and Methods |

| Human research participants | Information included in the manuscript? | In which section is the information available? (Reagents and Tools Table, Materials and Methods, Figures, Data Availability Section) |
|---|---|---|
| If collected and within the bounds of privacy constraints report on age, sex and gender or ethnicity for all study participants. | Not Applicable | |

| Core facilities | Information included in the manuscript? | In which section is the information available? (Reagents and Tools Table, Materials and Methods, Figures, Data Availability Section) |
|---|---|---|
| If your work benefited from core facilities, was their service mentioned in the acknowledgments section? | Yes | Acknowledgements |

## Design

| Study protocol | Information included in the manuscript? | In which section is the information available? (Reagents and Tools Table, Materials and Methods, Figures, Data Availability Section) |
| --- | --- | --- |
| If study protocol has been pre-registered, provide DOI in the manuscript. For clinical trials, provide the trial registration number OR cite DOI. | Not Applicable | |
| Report the clinical trial registration number (at ClinicalTrials.gov or equivalent), where applicable. | Not Applicable | |

| Laboratory protocol | Information included in the manuscript? | In which section is the information available? (Reagents and Tools Table, Materials and Methods, Figures, Data Availability Section) |
| --- | --- | --- |
| Provide DOI OR other citation details if external detailed step-by-step protocols are available. | Not Applicable | |

| Experimental study design and statistics | Information included in the manuscript? | In which section is the information available? (Reagents and Tools Table, Materials and Methods, Figures, Data Availability Section) |
| --- | --- | --- |
| Include a statement about sample size estimate even if no statistical methods were used. | Not Applicable | |
| Were any steps taken to minimize the effects of subjective bias when allocating animals/samples to treatment (e.g. randomization procedure)? If yes, have they been described? | Not Applicable | |
| Include a statement about blinding even if no blinding was done. | Yes | Materials and Methods |
| Describe inclusion/exclusion criteria if samples or animals were excluded from the analysis. Were the criteria pre-established?<br><br>If sample or data points were omitted from analysis, report if this was due to attrition or intentional exclusion and provide justification. | Not Applicable | |
| For every figure, are statistical tests justified as appropriate? Do the data meet the assumptions of the tests (e.g., normal distribution)? Describe any methods used to assess it. Is there an estimate of variation within each group of data? Is the variance similar between the groups that are being statistically compared? | Yes | Figure Legends; Materials and Methods |

| Sample definition and in-laboratory replication | Information included in the manuscript? | In which section is the information available? (Reagents and Tools Table, Materials and Methods, Figures, Data Availability Section) |
| --- | --- | --- |
| In the figure legends: state number of times the experiment was replicated in laboratory. | Yes | Figure Legends |
| In the figure legends: define whether data describe technical or biological replicates. | Yes | Figure Legends |

## Ethics

| Ethics | Information included in the manuscript? | In which section is the information available? (Reagents and Tools Table, Materials and Methods, Figures, Data Availability Section) |
| --- | --- | --- |
| Studies involving human participants: State details of authority granting ethics approval (IRB or equivalent committee(s), provide reference number for approval. | Not Applicable | |
| Studies involving human participants: Include a statement confirming that informed consent was obtained from all subjects and that the experiments conformed to the principles set out in the WMA Declaration of Helsinki and the Department of Health and Human Services Belmont Report. | Not Applicable | |
| Studies involving human participants: For publication of patient photos, include a statement confirming that consent to publish was obtained. | Not Applicable | |
| Studies involving experimental animals: State details of authority granting ethics approval (IRB or equivalent committee(s), provide reference number for approval. Include a statement of compliance with ethical regulations. | Not Applicable | |
| Studies involving specimen and field samples: State if relevant permits obtained, provide details of authority approving study; if none were required, explain why. | Not Applicable | |

| Dual Use Research of Concern (DURC) | Information included in the manuscript? | In which section is the information available? (Reagents and Tools Table, Materials and Methods, Figures, Data Availability Section) |
| --- | --- | --- |
| Could your study fall under dual use research restrictions? Please check biosecurity documents and list of select agents and toxins (CDC): https://www.selectagents.gov/sat/list.htm | Not Applicable | |
| If you used a select agent, is the security level of the lab appropriate and reported in the manuscript? | Not Applicable | |
| If a study is subject to dual use research of concern regulations, is the name of the authority granting approval and reference number for the regulatory approval provided in the manuscript? | Not Applicable | |

## Reporting

The MDAR framework recommends adoption of discipline-specific guidelines, established and endorsed through community initiatives. Journals have their own policy about requiring specific guidelines and recommendations to complement MDAR.

| Adherence to community standards | Information included in the manuscript? | In which section is the information available? (Reagents and Tools Table, Materials and Methods, Figures, Data Availability Section) |
| --- | --- | --- |
| State if relevant guidelines or checklists (e.g., ICMJE, MIBBI, ARRIVE, PRISMA) have been followed or provided. | Not Applicable | |
| For tumor marker prognostic studies, we recommend that you follow the REMARK reporting guidelines (see link list at top right). See author guidelines, under 'Reporting Guidelines'. Please confirm you have followed these guidelines. | Not Applicable | |
| For phase II and III randomized controlled trials, please refer to the CONSORT flow diagram (see link list at top right) and submit the CONSORT checklist (see link list at top right) with your submission. See author guidelines, under 'Reporting Guidelines'. Please confirm you have submitted this list. | Not Applicable | |

## Data Availability

| Data availability | Information included in the manuscript? | In which section is the information available? (Reagents and Tools Table, Materials and Methods, Figures, Data Availability Section) |
| --- | --- | --- |
| Have primary datasets been deposited according to the journal's guidelines (see 'Data Deposition' section) and the respective accession numbers provided in the Data Availability Section? | Yes | Data Availability Section |
| Were human clinical and genomic datasets deposited in a public access-controlled repository in accordance to ethical obligations to the patients and to the applicable consent agreement? | Not Applicable | |
| Are computational models that are central and integral to a study available without restrictions in a machine-readable form? Were the relevant accession numbers or links provided? | Yes | Data Availability Section |
| If publicly available data were reused, provide the respective data citations in the reference list. | Not Applicable | |
