# Supplementary material for: LC3 forms functional nanoclusters on autophagosomes
Source: EMBO Rep. 2026 Sep 25;27(21):6139–65. doi: 10.1038/s44319-026-00931-0 (PMC13743770; doi:10.1038/s44319-026-00931-0)
Supplement: Supplementary file 5 [file 44319_2026_931_MOESM11_ESM.zip › Movie_EV7/Movie EV7.docx]

**Movie EV7**

The movie shows TIRF movie of cells showing GFP-LC3**^CterMutant^** positive autophagosomes captured at 100 msec exposure time. The playback speed is 10x real-time. Scale 10 μm.
