# Supplementary material for: LC3 forms functional nanoclusters on autophagosomes
Source: EMBO Rep. 2026 Sep 25;27(21):6139–65. doi: 10.1038/s44319-026-00931-0 (PMC13743770; doi:10.1038/s44319-026-00931-0)
Supplement: Supplementary file 11 [file 44319_2026_931_MOESM17_ESM.zip › Movie_EV16/Movie EV16.docx]

**Movie EV16**

The movie shows TIRF movie of representative temporal color-coded trajectory of EGFP–LC3B^WT^ positive autophagosome shown in Fig S33 B. The playback speed is 10x real-time. Scale 200 nm
