# Supplementary material for: LC3 forms functional nanoclusters on autophagosomes
Source: EMBO Rep. 2026 Sep 25;27(21):6139–65. doi: 10.1038/s44319-026-00931-0 (PMC13743770; doi:10.1038/s44319-026-00931-0)
Supplement: Supplementary file 13 [file 44319_2026_931_MOESM19_ESM.zip › Movie_EV13/Movie EV13.docx]

**Movie EV13**

The movie shows TIRF movie of Neuro-2a cells showing GFP- LC3**^Loop6Mutant1^** positive autophagosomes captured at 100 msec exposure time. The playback speed is 60x real-time. Scale 5 μm.
