# Supplementary material for: LC3 forms functional nanoclusters on autophagosomes
Source: EMBO Rep. 2026 Sep 25;27(21):6139–65. doi: 10.1038/s44319-026-00931-0 (PMC13743770; doi:10.1038/s44319-026-00931-0)
Supplement: Supplementary file 21 [file 44319_2026_931_MOESM5_ESM.zip › Movie_EV1/Movie EV1.docx]

**Movie EV1**

Time evolution of LC3B cluster formation observed in the 10µs coarse-grained molecular simulation trajectory.
