# Supplementary material for: LC3 forms functional nanoclusters on autophagosomes
Source: EMBO Rep. 2026 Sep 25;27(21):6139–65. doi: 10.1038/s44319-026-00931-0 (PMC13743770; doi:10.1038/s44319-026-00931-0)
Supplement: Supplementary file 24 [file 44319_2026_931_MOESM8_ESM.zip › Movie_EV4/Movie EV4.docx]

**Movie EV4**

The movie shows ROI 3 from a 3D STORM image of endogenous LC3 in the XY and YZ plane rotations.
